# *B*–N/*B*–H Transborylation: borane-catalysed nitrile hydroboration

**DOI:** 10.3762/bjoc.18.138

**Published:** 2022-09-26

**Authors:** Filip Meger, Alexander C W Kwok, Franziska Gilch, Dominic R Willcox, Alex J Hendy, Kieran Nicholson, Andrew D Bage, Thomas Langer, Thomas A Hunt, Stephen P Thomas

**Affiliations:** 1 EaStCHEM School of Chemistry, University of Edinburgh, Edinburgh, EH9 3FJ, United Kingdom; 2 Pharmaceutical Technology & Development, Chemical Development U.K., AstraZeneca, Macclesfield, SK10 2NA, United Kingdom; 3 Medicinal Chemistry, Early Oncology, AstraZeneca, Cambridge, CB4 0WG, United Kingdom

**Keywords:** boron, catalysis, hydroboration, nitrile, transborylation

## Abstract

The reduction of nitriles to primary amines is a useful transformation in organic synthesis, however, it often relies upon stoichiometric reagents or transition-metal catalysis. Herein, a borane-catalysed hydroboration of nitriles to give primary amines is reported. Good yields (48–95%) and chemoselectivity (e.g., ester, nitro, sulfone) were observed. DFT calculations and mechanistic studies support the proposal of a double *B*–N/*B*–H transborylation mechanism.

## Introduction

**Scheme 1:**
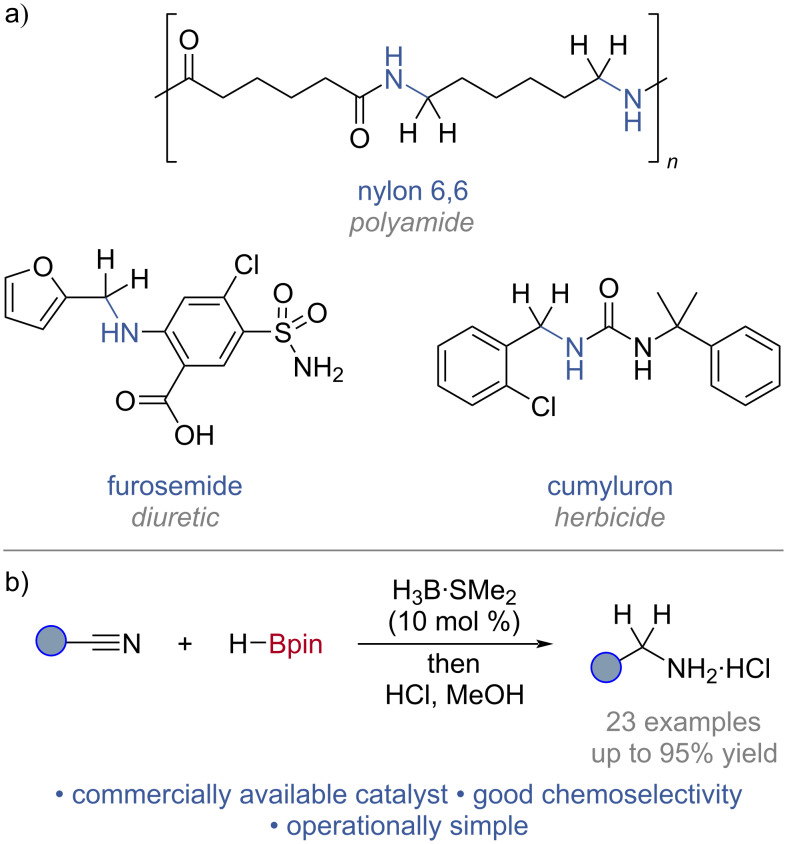
a) Derivatives of primary amines in materials chemistry, pharmaceuticals, and agrochemicals; b) this work: borane-catalysed nitrile hydroboration.

Primary amines are prevalent throughout organic synthesis, finding regular application in materials chemistry, pharmaceuticals, and agrochemicals (Scheme 1a) [1–3]. Traditionally, the reduction of nitriles to primary amines relied on stoichiometric hydride reagents [4]. Current catalytic methods for nitrile reduction, hydrogenation [5–6] or hydroboration [7–8], generally rely on metal catalysts, designer ligands, forcing reaction conditions (such as elevated temperatures and pressures) or lack extensive functional group tolerance. In particular, catalysed nitrile hydroboration strategies are still underdeveloped compared with hydrogenation, but offer a nascent alternative to this established field. The pursuit of sustainable chemical transformations has driven research into the development of new catalytic methodologies, specifically those that use Earth-abundant and low toxicity elements, as an alternative to transition-metal catalysis [9–10]. Using metal-free catalytic systems, including boron-based catalysts, circumvents some of the issues associated with traditional metal catalysis, including trace metal contamination [11–12] and volatility of supply.

Whilst there are several examples of main-group catalysed nitrile hydroboration reactions [13–26], currently, there are only two reported metal-free catalytic strategies [27]. Both of these used non-commercially available catalysts prepared over multiple steps, the first an N-heterocyclic olefin [22], and the second a bicyclic (alkyl)(amino)carbine [23]. Both displayed limited tolerance to reducible functionalities. NaHBEt_3_ is the only known boron-based catalyst for nitrile hydroboration [24]. Developed from the stoichiometric reactivity of [HBEt_3_]^−^, this method displayed good reactivity but limited chemoselectivity. Like [HBEt_3_]^−^, boranes are stoichiometric reducing agents, but are considered to be milder than [HBEt_3_]^−^ [4]. Catalysis enabled by *B*–N/*B*–H transborylation (a σ-bond metathesis turnover method) has been used for borane-catalysed reductions of N-heteroarenes [28–29], and the borane-catalysed cyanation of enones [30]. Applying *B*–N/*B*–H transborylation to the hydroboration of nitriles would enable the development of a borane-catalysed hydroboration of nitriles with broad chemoselectivity, raise the standing of metal-free catalysis in nitrile hydroboration, and increase user accessibility by using commercially available catalysts and turnover reagents (Scheme 1b).

## Results and Discussion

Investigations began by screening a combination of commercially available (H_3_B·SMe_2_, [H-*B*-9-BBN]_2_, H_3_B·THF) and prepared borane catalysts (dicyclohexylborane) in the hydroboration of heptanenitrile with HBpin as the turnover reagent (see Supporting Information File 1 for details). All boranes were catalytically active, with H_3_B·SMe_2_ displaying the highest activity. As [H-*B*-9-BBN]_2_ shows limited stoichiometric activity in aromatic nitrile reduction compared with H_3_B·SMe_2_ [31], H_3_B·SMe_2_ was used as the catalyst for this transformation [32]. Reducing the catalyst loading from 10 mol % to 5 mol % resulted in a marginal decrease in yield (92% to 80%, see Supporting Information File 1) and decreasing the loading further (1 mol %) resulted in a significant reduction in the yield (<5%, see Supporting Information File 1). In line with the principles of sustainable synthesis [33], the reaction can be performed in the absence of solvent and the reaction temperature can be adjusted to maximise efficiency for yield or chemoselectivity. Reaction of the turnover reagent, HBpin, in the absence of catalyst gave no observable reduction (see Supporting Information File 1).

**Scheme 2:**
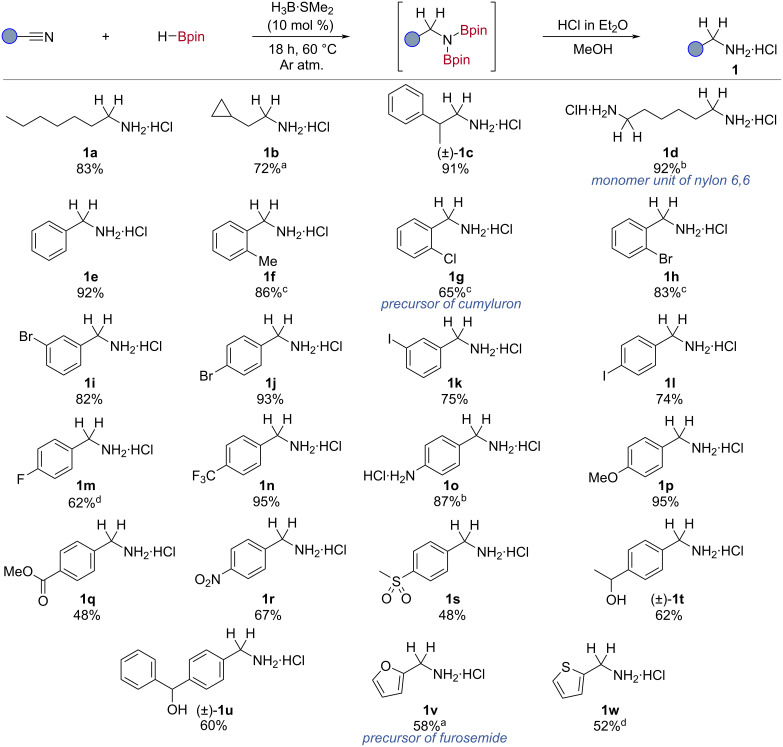
Substrate scope of borane-catalysed nitrile hydroboration with HBpin. Conditions: nitrile (0.50 mmol), HBpin (3.5 equiv), H_3_B·SMe_2_ (10 mol %), Ar atm., 60 °C, 18 h, unless otherwise noted. Isolated yields are reported. ^a^1.0 mmol scale. ^b^HBpin (5.5 equiv). ^c^80 °C. ^d^40 °C.

The optimised conditions were applied to a substrate scope, where the hydroboration products were converted in situ to the corresponding amine hydrochloride salt which can be isolated without the use of column chromatography (Scheme 2). The *N*,*N*-diborylamines have also been used directly in subsequent transformations [34–37]. Heptanenitrile was reduced to 1-heptylamine hydrochloride (**1a**) in good yield (83%). This catalytic protocol was applied to the reduction of other aliphatic nitriles to their corresponding amine hydrochloride salts, such as cyclopropyl-bearing (**1b**, 72%) and branched aliphatic nitriles (**1c**, 91%). Hexane-1,6-diamine dihydrochloride (**1d**), a monomer of nylon 6,6 [1], was generated in excellent yield (92%).

The reaction was successfully applied to the hydroboration of aryl nitriles; benzonitrile was reduced to benzylamine hydrochloride (**1e**, 92%) in good yield. The reaction tolerated substitution in the *ortho*- (**1f**, 86%, **1g**, 65%, **1h**, 83%), *meta*- (**1i**, 82%, **1k**, 75%), and *para*- (**1j**, 93%, **1l**, 74%) positions. An increased temperature of 80 °C was required for the efficient hydroboration for *ortho*-substituted aryl nitriles (**1f**, **1g**, **1h**), due to increased steric encumbrance close to the nitrile bond. The chloro-, bromo-, and iodo-substituted arenes provide a functional handle for further derivatisation. 2-Chlorobenzylamine hydrochloride, a precursor to the herbicide cumyluron, was isolated in good yield (**1g**, 65%) [38]. Additionally, fluoro-substituted nitriles were also reduced with good efficiency (**1m**, 62%).

Both electron-withdrawing (**1n**, 95%) and electron-donating (**1o**, 87%, **1p**, 95%) groups were tolerated under the reaction conditions. The presence of a primary amine in the substrate was tolerated under the reaction conditions (**1o**), undergoing efficient hydroboration to give the dihydrochloride salt (87%), although an increase in HBpin equivalents (5.5 equiv) was required due to dehydrocoupling of the turnover reagent, HBpin, with the amine [39].

A range of nitriles containing functional groups were tested in catalysis to investigate the chemoselectivity of the reaction. Several reducible functionalities were tolerated by the catalytic protocol, including ester (**1q**, 48%), nitro (**1r**, 67%), and sulfone (**1s**, 48%). The ^1^H NMR spectra of the crude reaction mixtures displayed no reduction of these functional groups. Such functional group tolerance was not exhibited by the other non-metal and boron-based catalytic nitrile hydroboration systems [22–24,27]. It should be noted that the borohydride-catalysed reductions generally proceed at lower reaction temperatures using 5 mol % NaBHEt_3_ [24]. Esters undergo stoichiometric reduction by H_3_B·SMe_2_ (see Supporting Information File 1) [32]; therefore, chemoselective nitrile hydroboration highlights the benefit of this catalytic method over stoichiometric reduction. However, chemoselectivity was not observed for ketone bearing substrates, resulting in the reduction of both the carbonyl and nitrile functionalities, although the resulting hydrochloride salts could be isolated in good yields (**1t**, 62%, **1u**, 60%). This is comparable to the stoichiometric reactivity of boranes [32].

Nitriles containing heterocycles underwent hydroboration in moderate yield (52–58%), with furan and thiophene groups tolerated (**1v**, **1w**). Furan-2-ylmethanamine hydrochloride **1v** can be converted into furosemide, a diuretic on the WHO list of essential medicines with over 28 million prescriptions per annum, in one step [40].

**Scheme 3:**
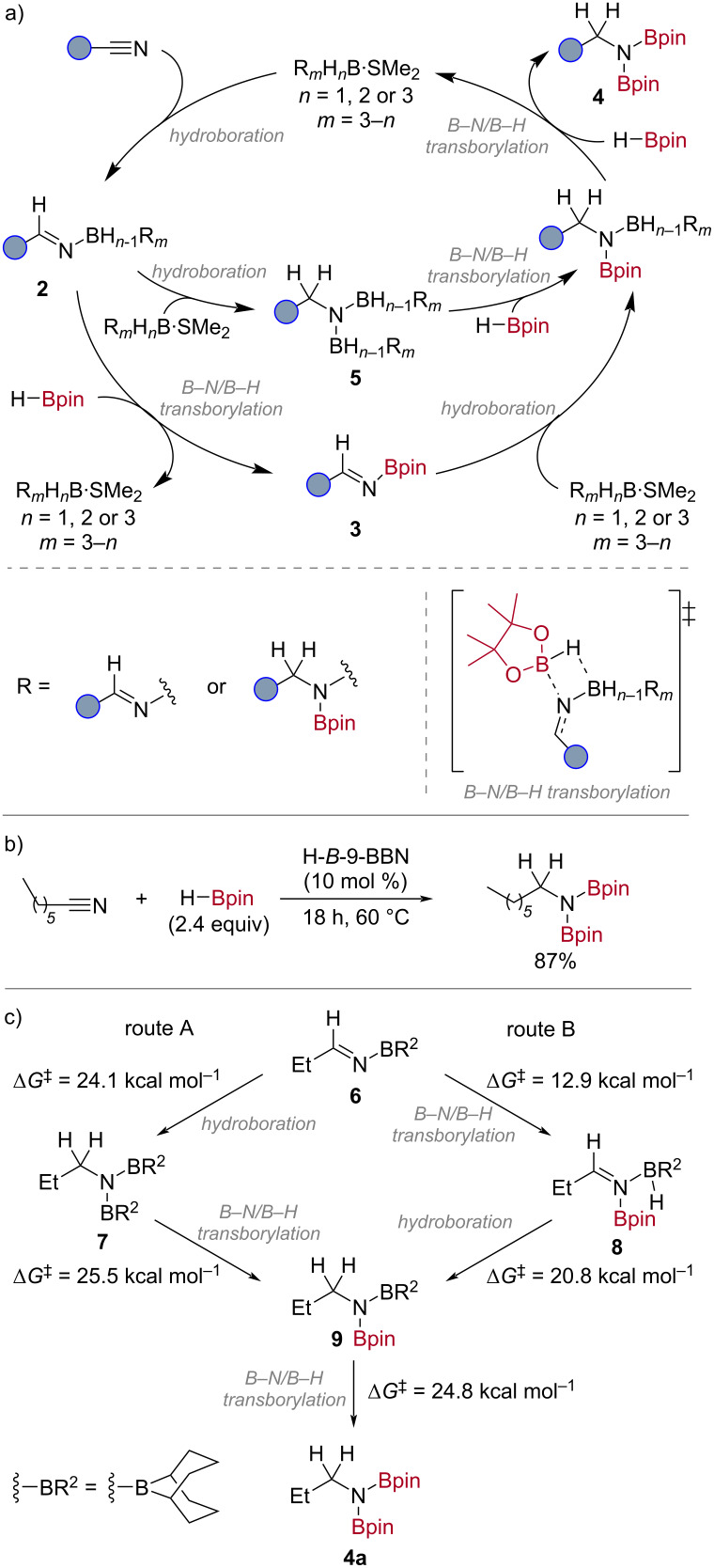
a) Proposed mechanism; b) H-*B*-9-BBN-catalysed heptanenitrile hydroboration (yield determined by ^1^H NMR spectroscopy using 1,3,5-trimethoxybenzene as an internal standard); c) key steps in the DFT-computed free energies (energies calculated at M06-2X/6-311++G(d,p) on M06-2X/6-31+G(d,p)-optimised structures).

The reaction is proposed to proceed through a hydroboration and *B*–N/*B*–H transborylation mechanism, supported by computational analyses (Scheme 3a). The nitrile undergoes hydroboration with H_3_B·SMe_2_ to form an *N*-borylimine species **2**. This can undergo *B*–N/*B*–H transborylation with HBpin to give the *N*-Bpin imine **3**, followed by a second hydroboration and *B*–N/*B*–H transborylation to give the *N*,*N*-bis-Bpin amine product **4**. Alternatively, the imido-boryl intermediate **2** undergoes a second hydroboration to form the *N*,*N*-bis-borylamine **5** prior to *B*–N/*B*–H transborylation.

Given the multiple mechanistic pathways for the reaction, DFT analysis [M06-2X/6-311++G(d,p)] [41–42] was used to differentiate between these two pathways. 9-Borabicyclo[3.3.1]nonane ([H-*B*-9-BBN]_2_) was chosen as the catalyst for the calculations due to its comparable activity to H_3_B·SMe_2_ (Scheme 3b) and the reduced number of reactive B–H sites to reduce complexity. Following hydroboration of the nitrile by H-*B*-9-BBN to give the *N*-B-9-BBN imine **6**, the reaction can proceed by two routes to give the *N*,*N*-bis-Bpin amine **4a**: A) a second hydroboration by H-*B*-9-BBN to give *N*,*N*-bis-*B*-9-BBN amine **7** and two *B*–N/*B*–H transborylation reactions with HBpin; B) *B*–N/*B*–H transborylation with HBpin to give *N*-Bpin imine **8** and a further hydroboration by H-*B*-9-BBN and *B*–N/*B*–H transborylation (Scheme 3c). Route A was found to have a highest energy barrier of 25.5 kcal mol^−1^ for the *B*–N/*B*–H transborylation step (**7** to **9**). In contrast, the highest energy barrier in route B was found to be the second *B*–N/*B*–H transborylation step (**9** to **4a**), which is also present in route A as the two routes converge, with a barrier of 24.8 kcal mol^−1^. The similarity in these energy barriers and the reaction temperature of 60 °C means both routes are viable under the reaction conditions. It should be noted that treatment of a nitrile with excess H-*B*-9-BBN has been shown to form an N-*B*-9-BBN iminyl H-*B*-9-BBN adduct [43], suggesting route B may be favoured. Experimental calculation of the reaction activation energy supported the proposed routes (see Supporting Information File 1 for details).

## Conclusion

In summary, a borane-catalysed hydroboration of nitriles to give primary amines has been developed, transforming the previously stoichiometric reagent H_3_B·SMe_2_ into a catalyst. *B*-N/*B*–H transborylation is proposed to serve as the key turnover step in catalysis, supported by computational mechanistic studies. This approach uses both a commercially-available catalyst and turnover reagent, providing good user accessibility, and displays comparable chemoselectivity to current state-of-the-art catalysed hydroboration methods [7].

## Author Contributions

F. M., A. C. W. K., F. G., A. J. H., K. N., and A. D. B. completed all practical laboratory work. D. R. W. completed all computational analysis. A. D. B., K. N., D. R. W., and S. P. T. conceived the reactions and wrote the manuscript. S. P. T., T. A. H., and T. L. advised investigations.

## Supporting Information

File 1 Experimental details, characterisation data, and copies of NMR spectra.
