# Supplementary material for: B–N/B–H Transborylation: borane-catalysed nitrile hydroboration
Source: Beilstein J Org Chem. 2022 Sep 26;18:1332–7. doi: 10.3762/bjoc.18.138 (PMC9531558; doi:10.3762/bjoc.18.138)
Supplement: File 1 — Experimental details, characterisation data, and copies of NMR spectra. [file Beilstein_J_Org_Chem-18-1332-s001.pdf]

BEILSTEIN JOURNAL OF ORGANIC CHEMISTRY

Supporting Information

for

B–N/*B*–H Transborylation: borane-catalysed nitrile hydroboration

Filip Meger, Alexander C. W. Kwok, Franziska Gilch, Dominic R. Willcox, Alex J. Hendy, Kieran Nicholson, Andrew D. Bage, Thomas Langer, Thomas A. Hunt and Stephen P. Thomas

Beilstein J. Org. Chem. **2022,** *18,* 1332–1337. doi:10.3762/bjoc.18.138

Experimental details, characterisation data, and copies of NMR spectra

License and Terms: This is a supporting information file under the terms of the Creative Commons Attribution License (https://creativecommons.org/licenses/by/4.0). Please note that the reuse, redistribution and reproduction in particular requires that the author(s) and source are credited and that individual graphics may be subject to special legal provisions.
The license is subject to the *Beilstein Journal of Organic Chemistry* terms and conditions: (https://www.beilstein-journals.org/bjoc/terms)

Table of contents

S1 General experimental S1

S2 Reaction optimisation S2

S3 General procedure for the hydroboration of nitriles S3

S4 Preparation and characterisation of hydroboration products S4

S5 Stoichiometric reduction of methyl 4-cyanobenzoate S27

S6 Kinetic analysis S28

S6.1 Experimental details S28

S6.2 Reaction profile over varied temperatures and Arrhenius plot S28

S6.3 Arrhenius calculation S28

S7 Computational details S29

Figure S1. DFT-Computed free energies of nitrile hydroboration. S30

Figure S2. DFT-Computed free energies of imine hydroboration. S30

Figure S3. DFT-Computed free energies of transborylation from mixed bis-borylamine IM7. .... S31

Cartesian coordinates S32

S8 NMR Spectra S103

S9 References S128

S1 General experimental

Reaction setup: All reactions were performed in oven (180 °C) and/or flamed-dried glassware under an atmosphere of anhydrous argon or nitrogen, unless otherwise indicated. All air and moisture-sensitive reactions were carried out using standard vacuum line and Schlenk techniques, or in a glovebox with a purified argon atmosphere. All glassware was cleaned using base (KOH, iPrOH) and acid (HCl_{aq}) baths. All reported reaction temperatures correspond to external oil bath temperatures. Room temperature (rt) was approximately 20 °C.

NMR Spectroscopy: ^{1}H, ^{13}C, and ^{19}F NMR spectra were recorded on Bruker Avance III 400 and 500 MHz or Bruker Avance I 600 MHz spectrometers. Chemical shifts are reported in parts per million (ppm). ^{1}H NMR spectra were referenced to the residual proteosolvent peak (methanol: 3.31 ppm; H_2O: 4.79 ppm; DMSO: 2.50 ppm). ^{13}C NMR spectra were referenced to the solvent peak (methanol-d_4: 49.00 ppm; DMSO-d_6: 39.52 ppm) and are ^{1}H decoupled, unless stated. Multiplicities are indicated by br. (broad), s (singlet), d (doublet), t (triplet), q (quartet), p (pentet), sext. (sextet), sept. (septet), m (multiplet), app. (apparent). Coupling constants, J, are reported in hertz and rounded to the nearest 0.1 Hz.

Infrared spectroscopy: Infrared (IR) spectra were recorded on a Perkin-Elmer Spectrum One FT-IR or Shimadzu IRAffinity-1 spectrometer. Peaks are reported in cm^{-1} with indicated relative intensities: s (strong, 0–33% T), m (medium, 34–66% T), w (weak, 67–100% T), and br. (broad).

Mass spectrometry: Mass spectrometry (MS) was performed by the University of Edinburgh, School of Chemistry, Mass Spectrometry Laboratory. High resolution mass spectra were recorded on a VG autospec, or Thermo/Finnigan MAT 900 mass spectrometer. Electron Impact (EI^+) spectrometry was performed at 70 eV using methane as the carrier gas, with either a double focusing sector field (DFSF) or time-of-flight (TOF) mass analyser. Electrospray ionization (ESI^+) spectra were performed using a time-of-flight (TOF) mass analyser. Data are reported in the form of m/z (intensity relative to the base peak = 100).

Solvents: All solvents for air and moisture-sensitive techniques were obtained from an anhydrous solvent system (Innovative Technology). Reaction solvent tetrahydrofuran (THF, Fisher, HPLC grade) was dried by percolation through two columns packed with neutral alumina under a positive pressure of argon. Solvents for filtration, transfers, and recrystallisation were ethyl acetate (EtOAc, Fisher, ACS grade), hexane (Optima), methanol (MeOH, ACS grade), and petroleum ether (40–60 °C, ACS grade).

Chemicals: All reagents were purchased from Sigma Aldrich, Alfa Aesar, Acros organics, Tokyo Chemical Industries UK, Fluorochem, Fisher Scientific, and Apollo Scientific, or synthesised within the laboratory.

S2 Reaction optimisation

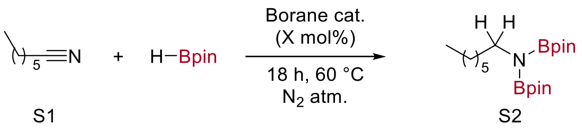

Heptanenitrile (**S1**, 69 µL, 0.50 mmol), pinacolborane, and a borane catalyst were reacted under a N_2 atmosphere. The reactions were quenched with a solution of 1,3,5-trimethoxybenzene in dichloromethane (0.10 M, 0.50 mL, 0.050 mmol) and the yield was determined by ^{1}H NMR spectroscopy, using 1,3,5-trimethoxybenzene as an internal standard, performing the reaction in duplicate.

Table S1 Optimisation of reaction conditions.

Entry	Catalyst	Cat. Loading (mol%)	HBpin (Equiv.)	Reaction Time (h)	Temperature (°C)	Yield of **S2** (%)
1	$H_3B·SMe_2$	10	2.4	18	60	92
2	H-*B*-9-BBN (0.5 M THF)	10	2.4	18	60	87
3	$HBCy_2$	10	2.4	18	80	66
4	$H_3B·THF$ (1.0 M THF)	10	2.4	18	60	25
5	$H_3B·SMe_2$	1	2.4	18	60	<5
6	$H_3B·SMe_2$	5	2.4	18	60	80
7	$H_3B·SMe_2$	10	2.4	6	60	63
8	$H_3B·SMe_2$	10	2.4	18	40	66
9	$H_3B·SMe_2$	10	2.4	18	20	21
10	$H_3B·SMe_2$	10	2.6	18	40	80
11	$H_3B·SMe_2$	10	3.0	18	40	86
12	$H_3B·SMe_2$	10	3.5	18	40	>95
13	No catalyst	0	3.5	18	80	trace

.

S3 General procedure for the hydroboration of nitriles

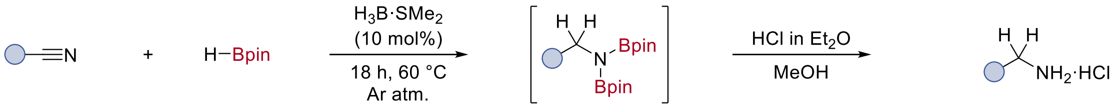

1

Nitrile (0.50 mmol), pinacolborane (0.25 mL, 1.7 mmol), and borane dimethyl sulfide (5 µL, 0.05 mmol) were reacted at 60 °C for 18 hours under an argon atmosphere. The reaction mixture was allowed to cool to room temperature, and hydrogen chloride (2 M in diethyl ether, 4 mL) and methanol (2 mL) were added. The reaction mixture was stirred for 1 hour at room temperature, then concentrated in vacuo. The residue was washed with cold ethyl acetate, hexane, or petroleum ether (2 × 10 mL), filtered, and dried in vacuo to give the amine hydrochloride salt product **1**.

S4 Preparation and characterisation of hydroboration products

Heptan-1-amine hydrochloride (1a)

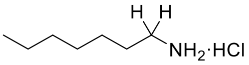

According to the general procedure, heptanenitrile (69 µL, 0.50 mmol), pinacolborane (0.25 mL, 1.7 mmol), and borane dimethyl sulfide (5 µL, 0.05 mmol) were reacted at 60 °C for 18 hours under an argon atmosphere. The reaction was allowed to cool to room temperature, and hydrogen chloride (2 M in diethyl ether, 4 mL) and methanol (2 mL) were added. The reaction was stirred for 1 hour at room temperature then concentrated in vacuo. The residue was washed with cold hexane (2 × 10 mL), filtered, and dried in vacuo to give heptan-1-amine hydrochloride (**1a**, 63 mg, 0.42 mmol, 83%) as a white amorphous solid.

^{1}H NMR (400 MHz, Methanol-*d*4)
2.93 (t, *J* = 7.7 Hz, 2H), 1.68 (p, *J* = 7.2 Hz, 2H), 1.46 – 1.25 (m, 8H), 0.96 – 0.86 (m, 3H).

^{13}C NMR (126 MHz, Methanol-*d*4)
40.8, 32.7, 29.9, 28.5, 27.4, 23.6, 14.4.

Data were in accordance with those previously reported.[1]

2-Cyclopropylethanamine hydrochloride (1b)

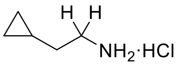

According to a modification of the general procedure, 2-cyclopropylacetonitrile (92 µL, 1.0 mmol), pinacolborane (0.51 mL, 3.5 mmol), and borane dimethyl sulfide (10 µL, 0.10 mmol) were reacted at 60 °C for 18 hours under an argon atmosphere. The reaction was allowed to cool to room temperature, and hydrogen chloride (2 M in diethyl ether, 8 mL) and methanol (4 mL) were added. The reaction was stirred for 1 hour at room temperature then concentrated in vacuo. The residue was washed with cold ethyl acetate (2 × 10 mL), filtered, and dried in vacuo to give 2-cyclopropylethanamine hydrochloride (**1b**, 88 mg, 0.72 mmol, 72%) as a white amorphous solid.

**^{1}H NMR**	(600 MHz, D_2O)
	3.13 (t, *J* = 7.1 Hz, 2H), 1.60 (q, *J* = 7.1 Hz, 2H), 0.82 – 0.73 (m, 1H), 0.58 – 0.53 (m, 2H), 0.18 – 0.14 (m, 2H).
**^{13}C NMR**	(151 MHz, D_2O)
	39.8, 31.4, 7.1, 3.4.

Data were in accordance with those previously reported.[2]

***rac*-2-Phenylpropan-1-amine hydrochloride (1c)**

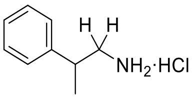

According to the general procedure, 2-phenylpropionitrile (67 µL, 0.50 mmol), pinacolborane (0.25 mL, 1.7 mmol), and borane dimethyl sulfide (5 µL, 0.05 mmol) were reacted at 60 °C for 18 hours under an argon atmosphere. The reaction was allowed to cool to room temperature, and hydrogen chloride (2 M in diethyl ether, 4 mL) and methanol (2 mL) were added. The reaction was stirred for 1 hour at room temperature then concentrated in vacuo. The residue was washed with cold hexane (2 × 10 mL), filtered, and dried in vacuo to give *rac*-2-phenylpropan-1-amine hydrochloride (**1c**, 78 mg, 0.45 mmol, 91%) as a white amorphous solid.

^{1}H NMR (600 MHz, DMSO-d_6)
7.89 (br. s, 3H), 7.36 – 7.32 (m, 2H), 7.29 – 7.23 (m, 3H), 2.98 (s, 2H), 1.24 (d, J = 6.8 Hz, 3H).

^{13}C NMR (151 MHz, DMSO-d_6)
142.9, 128.9, 127.3, 127.2, 45.1, 37.7, 19.5.

Data were in accordance with those previously reported.[3]

Hexane-1,6-diamine dihydrochloride (1d)

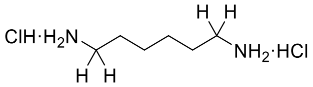

According to the general procedure, adiponitrile (57 µL, 0.50 mmol), pinacolborane (0.40 mL, 2.8 mmol), and borane dimethyl sulfide (5 µL, 0.05 mmol) were reacted at 60 °C for 18 hours under an argon atmosphere. The reaction was allowed to cool to room temperature, and hydrogen chloride (2 M in diethyl ether, 4 mL) and methanol (2 mL) were added. The reaction was stirred for 1 hour at room temperature then concentrated in vacuo. The residue was washed with cold ethyl acetate (2 × 10 mL), filtered, and dried in vacuo to give hexane-1,6-diamine dihydrochloride (**1d**, 87 mg, 0.46 mmol, 92%) as a white amorphous solid.

**^{1}H NMR**	(400 MHz, Methanol-*d*4)
	2.94 (t, *J* = 7.7 Hz, 4H), 1.75 – 1.64 (m, 4H), 1.50 – 1.42 (m, 4H).
**^{13}C NMR**	(151 MHz, Methanol-*d*4)
	40.6, 28.3, 26.9.

Data were in accordance with those previously reported.[4]

Benzylamine hydrochloride (1e)

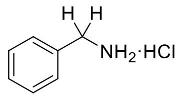

According to the general procedure, benzonitrile (52 µL, 0.50 mmol), pinacolborane (0.25 mL, 1.7 mmol), and borane dimethyl sulfide (5 µL, 0.05 mmol) were reacted at 60 °C for 18 hours under an argon atmosphere. The reaction was allowed to cool to room temperature, and hydrogen chloride (2 M in diethyl ether, 4 mL) and methanol (2 mL) were added. The reaction was stirred for 1 hour at room temperature then concentrated in vacuo. The residue was washed with cold ethyl acetate (2 × 10 mL), filtered, and dried in vacuo to give benzylamine hydrochloride (**1e**, 66 mg, 0.46 mmol, 92%) as a white amorphous solid.

^{1}H NMR (600 MHz, Methanol-d_4)
7.48 – 7.40 (m, 5H), 4.12 (s, 2H).

^{13}C NMR (151 MHz, Methanol-d_4)
134.4, 130.3, 130.2, 130.0, 44.4.

Data were in accordance with those previously reported.[4]

2-Methylbenzylamine hydrochloride (1f)

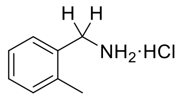

According to a modification of the general procedure, 2-methylbenzonitrile (59 µL, 0.50 mmol), pinacolborane (0.25 mL, 1.7 mmol), and borane dimethyl sulfide (5 µL, 0.05 mmol) were reacted at 80 °C for 18 hours under an argon atmosphere. The reaction was allowed to cool to room temperature, and hydrogen chloride (2 M in diethyl ether, 4 mL) and methanol (2 mL) were added. The reaction was stirred for 1 hour at room temperature then concentrated in vacuo. The residue was washed with cold ethyl acetate (2 × 10 mL), filtered, and dried in vacuo to give 2-methylbenzylamine hydrochloride (**1f**, 67 mg, 0.43 mmol, 86%) as a white amorphous solid.

**^{1}H NMR**	(600 MHz, Methanol-d_4)
	7.38 (d, J = 7.5, 1H), 7.33 – 7.26 (m, 3H), 4.16 (s, 2H), 2.42 (s, 3H).
**^{13}C NMR**	(151 MHz, Methanol-d_4)
	138.2, 132.6, 132.0, 130.4, 130.2, 127.8, 41.6, 19.0.

Data were in accordance with those previously reported.[5]

2-Chlorobenzylamine hydrochloride (1g)

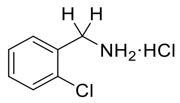

According to a modification of the general procedure, 2-chlorobenzonitrile (69 mg, 0.50 mmol), pinacolborane (0.25 mL, 1.7 mmol), and borane dimethyl sulfide (5 µL, 0.05 mmol) were reacted at 80 °C for 18 hours under an argon atmosphere. The reaction was allowed to cool to room temperature, and hydrogen chloride (2 M in diethyl ether, 4 mL) and methanol (2 mL) were added. The reaction was stirred for 1 hour at room temperature then concentrated in vacuo. The residue was washed with cold ethyl acetate (2 × 10 mL), filtered, and dried in vacuo to give 2-chlorobenzylamine hydrochloride (**1g**, 58 mg, 0.33 mmol, 65%) as a white amorphous solid.

^{1}H NMR (500 MHz, Methanol-d4)
7.58 – 7.51 (m, 2H), 7.44 (pd, J = 7.5, 1.8 Hz, 2H), 4.28 (s, 2H).

^{13}C NMR (126 MHz, Methanol-d_4)
135.3, 132.2, 132.1, 131.1, 129.0, 41.7.
6 resonances are reported rather than the expected 7 due to overlapping peaks

Data were in accordance with those previously reported.[6]

2-Bromobenzylamine hydrochloride (1h)

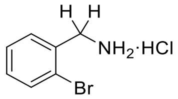

According to a modification of the general procedure, 2-bromobenzonitrile (91 mg, 0.50 mmol), pinacolborane (0.25 mL, 1.7 mmol), and borane dimethyl sulfide (5 µL, 0.05 mmol) were reacted at 80 °C for 18 hours under an argon atmosphere. The reaction was allowed to cool to room temperature, and hydrogen chloride (2 M in diethyl ether, 4 mL) and methanol (2 mL) were added. The reaction was stirred for 1 hour at room temperature then concentrated in vacuo. The residue was washed with cold ethyl acetate (2 × 10 mL), filtered, and dried in vacuo to give 2-bromobenzylamine hydrochloride (**1h**, 92 mg, 0.41 mmol, 83%) as a white amorphous solid.

**^{1}H NMR**	(600 MHz, D_2O)
	7.78 (d, J = 8.1 Hz, 1H), 7.56 (dd, J = 7.7, 1.7 Hz, 1H), 7.50 (tt, J = 7.6, 1.1 Hz, 1H), 7.41 (td, J = 7.7, 1.7 Hz, 1H), 4.38 (s, 2H).
**^{13}C NMR**	(151 MHz, D_2O)
	133.3, 131.9, 131.3, 131.1, 128.4, 123.8, 43.2.

Data were in accordance with those previously reported.[7]

3-Bromobenzylamine hydrochloride (1i)

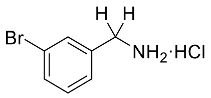

According to the general procedure, 3-bromobenzonitrile (91 mg, 0.50 mmol), pinacolborane (0.25 mL, 1.7 mmol), and borane dimethyl sulfide (5 µL, 0.05 mmol) were reacted at 60 °C for 18 hours under an argon atmosphere. The reaction was allowed to cool to room temperature, and hydrogen chloride (2 M in diethyl ether, 4 mL) and methanol (2 mL) were added. The reaction was stirred for 1 hour at room temperature then concentrated in vacuo. The residue was washed with cold ethyl acetate (2 × 10 mL), filtered, and dried in vacuo to give 3-bromobenzylamine hydrochloride (**1i**, 91 mg, 0.41 mmol, 82%) as a white amorphous solid.

^{1}H NMR (600 MHz, DMSO-d_6)
8.40 (br s, 3H), 7.73 (t, J = 1.9 Hz, 1H), 7.60 – 7.55 (m, 1H), 7.51 – 7.46 (m, 1H), 7.37 (t, J = 7.8 Hz, 1H), 4.02 (s, 2H).

^{13}C NMR (151 MHz, DMSO-d_6)
136.7, 131.9, 131.5, 130.9, 128.3, 121.8, 41.7.

Data were in accordance with those previously reported.[8]

4-Bromobenzylamine hydrochloride (1j)

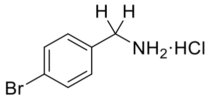

According to the general procedure, 4-bromobenzonitrile (91 mg, 0.50 mmol), pinacolborane (0.25 mL, 1.7 mmol), and borane dimethyl sulfide (5 µL, 0.05 mmol) were reacted at 60 °C for 18 hours under an argon atmosphere. The reaction was allowed to cool to room temperature, and hydrogen chloride (2 M in diethyl ether, 4 mL) and methanol (2 mL) were added. The reaction was stirred for 1 hour at room temperature then concentrated in vacuo. The residue was washed with cold ethyl acetate (2 × 10 mL), filtered, and dried in vacuo to give 4-bromobenzylamine hydrochloride (**1j**, 103 mg, 0.46 mmol, 93%) as a white amorphous solid.

**^{1}H NMR**	(400 MHz, D_2O)
	7.68 (d, J = 8.5 Hz, 2H), 7.40 (d, J = 8.4 Hz, 2H), 4.19 (s, 2H).
**^{13}C NMR**	(101 MHz, D_2O)
	132.2, 131.7, 130.7, 122.7, 42.5.

Data were in accordance with those previously reported.[9]

3-Iodobenzylamine hydrochloride (1k)

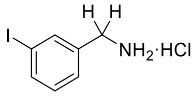

According to the general procedure, 3-iodobenzonitrile (115 mg, 0.50 mmol), pinacolborane (0.25 mL, 1.7 mmol), and borane dimethyl sulfide (5 µL, 0.05 mmol) were reacted at 60 °C for 18 hours under an argon atmosphere. The reaction was allowed to cool to room temperature, and hydrogen chloride (2 M in diethyl ether, 4 mL) and methanol (2 mL) were added. The reaction was stirred for 1 hour at room temperature then concentrated in vacuo. The residue was washed with cold ethyl acetate (2 × 10 mL), filtered, and dried in vacuo to give 3-iodobenzylamine hydrochloride (**1k**, 101 mg, 0.37 mmol, 75%) as a white amorphous solid.

**^{1}H NMR**	(500 MHz, Methanol-d_4)
	7.88 (t, *J* = 1.7 Hz, 1H), 7.79 (dt, *J* = 8.0, 1.3 Hz, 1H), 7.48 (dt, *J* = 7.8, 1.3 Hz, 1H), 7.23 (t, *J* = 7.8 Hz, 1H), 4.09 (s, 2H).
**^{13}C NMR**	(126 MHz, Methanol-d_4)
	139.4, 139.1, 136.8, 132.0, 129.4, 95.3, 43.5.
**IR ν_{max}**	(neat) 3310 (w), 2884 (w), 1595 (w), 1524 (w).
**MS**	(HRMS-ESI$^+$)
	Calculated for C_7H_9IN 233.9774; found 233.9778.

4-Iodobenzylamine hydrochloride (1l)

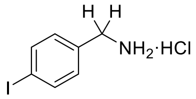

According to the general procedure, 4-iodobenzonitrile (115 mg, 0.50 mmol), pinacolborane (0.25 mL, 1.7 mmol), and borane dimethyl sulfide (5 µL, 0.05 mmol) were reacted at 60 °C for 18 hours under an argon atmosphere. The reaction was allowed to cool to room temperature, and hydrogen chloride (2 M in diethyl ether, 4 mL) and methanol (2 mL) were added. The reaction was stirred for 1 hour at room temperature then concentrated in vacuo. The residue was washed with cold ethyl acetate (2 × 10 mL), filtered, and dried in vacuo to give 4-iodobenzylamine hydrochloride (**1l,** 100 mg, 0.37 mmol, 74%) as a white amorphous solid.

^{1}H NMR (600 MHz, Methanol-d_4)
7.81 (d, J = 8.4 Hz, 2H), 7.24 (d, J = 8.3 Hz, 2H), 4.08 (s, 2H).

^{13}C NMR (151 MHz, Methanol-d_4)
139.5, 134.1, 132.0, 96.8, 43.4.

Data were in accordance with those previously reported.[10]

4-Fluorobenzylamine hydrochloride (1m)

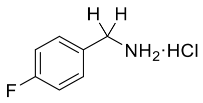

According to a modification of the general procedure, 4-fluorobenzonitrile (61 mg, 0.50 mmol), pinacolborane (0.25 mL, 1.7 mmol), and borane dimethyl sulfide (5 µL, 0.05 mmol) were reacted at 40 °C for 18 hours under an argon atmosphere. The reaction was allowed to cool to room temperature, and hydrogen chloride (2 M in diethyl ether, 4 mL) and methanol (2 mL) were added. The reaction was stirred for 1 hour at room temperature then concentrated in vacuo. The residue was washed with cold ethyl acetate (2 × 10 mL), filtered, and dried in vacuo to give 4-fluorobenzylamine hydrochloride (**1m**, 50 mg, 0.31 mmol, 62%) as a white amorphous solid.

^{1}H NMR (500 MHz, Methanol-*d*4)
7.49 (dd, *J* = 8.7, 5.2 Hz, 2H), 7.19 (t, *J* = 8.7 Hz, 2H), 4.11 (s, 2H).

^{13}C NMR (151 MHz, Methanol-*d*4)
164.6 (d, ^1J = 247.0 Hz), 132.4 (d, ^3J = 8.5 Hz), 130.6 (d, ^4J = 3.5 Hz), 117.0 (d, ^2J = 21.8 Hz), 43.6.

^{19}F NMR (376 MHz, Methanol-*d*4)
−114.6 (tt, *J* = 8.9, 5.2 Hz).

Data were in accordance with those previously reported.[11]

4-(Trifluoromethyl)benzylamine hydrochloride (1n)

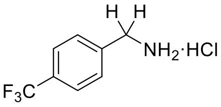

According to the general procedure, 4-(trifluoromethyl)benzonitrile (86 mg, 0.50 mmol), pinacolborane (0.25 mL, 1.7 mmol), and borane dimethyl sulfide (5 µL, 0.05 mmol) were reacted at 60 °C for 18 hours under an argon atmosphere. The reaction was allowed to cool to room temperature, and hydrogen chloride (2 M in diethyl ether, 4 mL) and methanol (2 mL) were added. The reaction was stirred for 1 hour at room temperature then concentrated in vacuo. The residue was washed with cold ethyl acetate (2 × 10 mL), filtered, and dried in vacuo to give 4-(trifluoromethyl)benzylamine hydrochloride (**1n**, 87 mg, 0.47 mmol, 95%) as a white amorphous solid.

^{1}H NMR (600 MHz, D_2O)
7.84 (d, J = 8.1 Hz, 2H), 7.66 (d, J = 8.1 Hz, 2H), 4.31 (s, 2H).

^{13}C NMR (151 MHz, D_2O)
136.7, 130.5 (d, ^2J= 32.2 Hz) 129.3, 126.1 (q, ^3J = 3.9 Hz), 124.0 (q, ^1J = 271.6 Hz), 42.6.

^{19}F NMR (376 MHz, D_2O)
−62.6.

Data were in accordance with those previously reported.[12]

4-Aminobenzylamine dihydrochloride (1o)

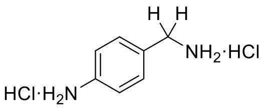

According to a modification of the general procedure, 4-aminobenzonitrile (59 mg, 0.50 mmol), pinacolborane (0.40 mL, 2.75 mmol), and borane dimethyl sulfide (5 µL, 0.05 mmol) were reacted at 60 °C for 18 hours under an argon atmosphere. The reaction was allowed to cool to room temperature, and hydrogen chloride (2 M in diethyl ether, 4 mL) and methanol (2 mL) were added. The reaction was stirred for 1 hour at room temperature then concentrated in vacuo. The residue was washed with cold ethyl acetate (2 × 10 mL), filtered, and dried in vacuo to give 4-aminobenzylamine dihydrochloride (**1o**, 85 mg, 0.44 mmol, 87%) as a yellow amorphous solid.

**^{1}H NMR**	(600 MHz, D_2O)
	7.63 (d, *J* = 8.5 Hz, 2H), 7.50 (d, *J* = 8.6 Hz, 2H), 4.28 (s, 2H).
**^{13}C NMR**	(151 MHz, D_2O)
	133.3, 131.3, 130.6, 123.5, 42.4.

Data were in accordance with those previously reported.[7]

4-Methoxybenzylamine hydrochloride (1p)

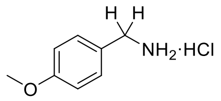

According to the general procedure, 4-methoxybenzonitrile (67 mg, 0.50 mmol), pinacolborane (0.25 mL, 1.7 mmol), and borane dimethyl sulfide (5 µL, 0.05 mmol) were reacted at 60 °C for 18 hours under an argon atmosphere. The reaction was allowed to cool to room temperature, and hydrogen chloride (2 M in diethyl ether, 4 mL) and methanol (2 mL) were added. The reaction was stirred for 1 hour at room temperature then concentrated in vacuo. The residue was washed with cold ethyl acetate (2 × 10 mL), filtered, and dried in vacuo to give 4-methoxybenzylamine hydrochloride (**1p**, 83 mg, 0.48 mmol, 95%) as a white amorphous solid.

**^{1}H NMR**	(500 MHz, D_2O)
	7.44 (d, *J* = 8.7 Hz, 2H), 7.08 (d, *J* = 8.7 Hz, 2H), 4.16 (s, 2H), 3.88 (s, 3H).
**^{13}C NMR**	(126 MHz, D_2O)
	159.4, 130.6, 125.2, 114.6, 55.4, 42.6.

Data were in accordance with those previously reported.[13]

Methyl 4-(aminomethyl)benzoate hydrochloride (1q)

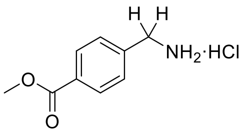

According to a modification of the general procedure, methyl 4-cyanobenzoate (81 mg, 0.50 mmol), pinacolborane (0.25 mL, 1.7 mmol), and borane dimethyl sulfide (5 µL, 0.05 mmol) were reacted at 60 °C for 18 hours under an argon atmosphere. The reaction was allowed to cool to room temperature, and hydrogen chloride (2 M in diethyl ether, 4 mL) and methanol (2 mL) were added. The reaction was stirred for 1 hour at room temperature then concentrated in vacuo. The residue was washed with cold ethyl acetate (5 mL) and petroleum ether (5 mL), filtered, and dried in vacuo to give 4-methyl 4-(aminomethyl)benzoate hydrochloride (**1q**, 48 mg, 0.24 mmol, 48%) as a white amorphous solid.

^{1}H NMR (400 MHz, Methanol-*d*4)
8.08 (d, *J* = 8.4 Hz, 2H), 7.60 (d, *J* = 8.7 Hz, 2H), 4.22 (s, 2H), 3.92 (s, 3H).

^{13}C NMR (126 MHz, Methanol-*d*4)
167.8, 139.5, 132.1, 131.2, 130.1, 52.8, 43.9.

Data were in accordance with those previously reported.[4]

4-Nitrobenzylamine hydrochloride (1r)

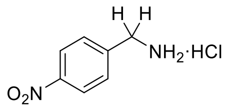

According to the general procedure, 4-nitrobenzonitrile (74 mg, 0.50 mmol), pinacolborane (0.25 mL, 1.7 mmol), and borane dimethyl sulfide (5 µL, 0.05 mmol) were reacted at 60 °C for 18 hours under an argon atmosphere. The reaction was allowed to cool to room temperature, and hydrogen chloride (2 M in diethyl ether, 4 mL) and methanol (2 mL) were added. The reaction was stirred for 1 hour at room temperature then concentrated in vacuo. The residue was washed with cold ethyl acetate (2 × 10 mL), filtered, and dried in vacuo to give 4-nitrobenzylamine hydrochloride (**1r,** 63 mg, 0.33 mmol, 67%) as a white amorphous solid.

^{1}H NMR (500 MHz, Methanol-d_4)
8.31 (d, J = 8.0 Hz, 2H), 7.73 (d, J = 8.6 Hz, 2H), 4.29 (s, 2H).

^{13}C NMR (126 MHz, Methanol-d_4)
149.7, 141.5, 131.2, 125.1, 43.4.

Data were in accordance with those previously reported.[2]

4-(Methylsulfonyl)benzylamine hydrochloride (1s)

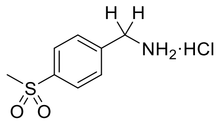

According to the general procedure, 4-(methylsulfonyl)benzonitrile (91 mg, 0.50 mmol), pinacolborane (0.25 mL, 1.7 mmol), and borane dimethyl sulfide (5 µL, 0.05 mmol) were reacted at 60 °C for 18 hours under an argon atmosphere. The reaction was allowed to cool to room temperature, and hydrogen chloride (2 M in diethyl ether, 4 mL) and methanol (2 mL) were added. The reaction was stirred for 1 hour at room temperature then concentrated in vacuo. The residue was washed with cold ethyl acetate (2 × 10 mL), filtered, and dried in vacuo to give 4-(methylsulfonyl)benzylamine hydrochloride (**1s**, 53 mg, 0.24 mmol, 48%) as a pale yellow amorphous solid.

^{1}H NMR (400 MHz, Methanol-d_4)
8.08 – 8.00 (m, 2H), 7.77 – 7.69 (m, 2H), 4.26 (s, 2H), 3.14 (s, 3H).

^{13}C NMR (126 MHz, Methanol-d_4)
142.8, 140.6, 131.0, 129.2, 44.2, 43.7.

Data were in accordance with those previously reported.[14]

rac-4-(1-Hydroxyethyl)benzylamine hydrochloride (1t)

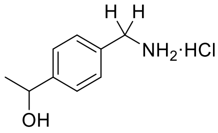

According to the general procedure, 4-acetylbenzonitrile (73 mg, 0.50 mmol), pinacolborane (0.25 mL, 1.7 mmol), and borane dimethyl sulfide (5 µL, 0.05 mmol) were reacted at 60 °C for 18 hours under an argon atmosphere. The reaction was allowed to cool to room temperature, and hydrogen chloride (2 M in diethyl ether, 4 mL) and methanol (2 mL) were added. The reaction was stirred for 1 hour at room temperature then concentrated in vacuo. The residue was washed with cold ethyl acetate (2 × 10 mL), filtered, and dried in vacuo to give *rac*-4-(1-hydroxyethyl)benzylamine hydrochloride (**1t**, 60 mg, 0.32 mmol, 62%) as a white amorphous solid.

^{1}H NMR (400 MHz, D_2O)
7.52 – 7.47 (m, 4H), 4.97 (q, *J* = 6.5 Hz, 1H), 4.21 (s, 2H), 1.51 (d, *J* = 6.5 Hz, 3H).

^{13}C NMR (101 MHz, D_2O)
146.0, 131.9, 129.1, 126.3, 69.4, 42.8, 23.6.

Data were in accordance with those previously reported.[15]

***rac*-4-(1-Hydroxy-1-phenylmethyl)benzylamine hydrochloride (1u)**

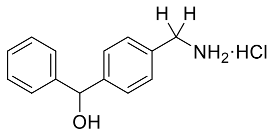

According to a modification of the general procedure, 4-benzoylbenzonitrile (104 mg, 0.50 mmol), pinacolborane (0.25 mL, 1.7 mmol), and borane dimethyl sulfide (5 µL, 0.05 mmol) were reacted at 60 °C for 18 hours under an argon atmosphere. The reaction was allowed to cool to room temperature, and hydrogen chloride (2 M in diethyl ether, 4 mL) and methanol (2 mL) were added. The reaction was stirred for 1 hour at room temperature then concentrated in vacuo. The residue was suspended with ethyl acetate (10 mL), cooled to −78 °C, filtered, and dried in vacuo to give *rac*-4-(1-hydroxy-1-phenylmethyl)benzylamine hydrochloride (**1u**, 75 mg, 0.30 mmol, 60%) as a white amorphous solid.

**^{1}H NMR**	(500 MHz, Methanol-d_4)
	8.31 (br. s, 1H), 7.49 – 7.44 (m, 4H), 7.38 – 7.35 (m, 2H), 7.30 (dd, J = 8.5, 6.8 Hz, 2H), 7.24 – 7.20 (m, 1H), 5.82 (s, 1H), 4.09 (s, 2H).
**^{13}C NMR**	(151 MHz, Methanol-d_4)
	147.2, 145.7, 133.1, 130.0, 129.3, 128.3, 128.3, 127.7, 76.4, 44.0.
**IR ν_{max}**	(neat) 3376 (w), 2963 (w), 2500 (w).
**MS**	(HRMS-ESI$^+$)
	Calculated for $C_{14}H_{16}NO$ 214.1230, found 214.1226.

Furan-2-ylmethanamine hydrochloride (1v)

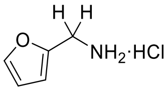

According to the general procedure, 2-furonitrile (88 µL, 1.0 mmol), pinacolborane (0.51 mL, 3.5 mmol), and borane dimethyl sulfide (10 µL, 0.10 mmol) were reacted at 60 °C for 18 hours under an argon atmosphere. The reaction was allowed to cool to room temperature, and hydrogen chloride (2 M in diethyl ether, 4 mL) and methanol (2 mL) were added. The reaction was stirred for 1 hour at room temperature then concentrated in vacuo. The residue was washed with cold ethyl acetate (2 × 10 mL), filtered, and dried in vacuo to give furan-2-ylmethanamine hydrochloride (**1v**, 77 mg, 0.58 mmol, 58%) as a black amorphous solid.

^{1}H NMR (600 MHz, D_2O)
7.63 – 7.61 (m, 1H), 6.60 (d, J = 3.3 Hz, 1H), 6.53 (dd, J = 3.4, 1.8 Hz, 1H), 4.27 (s, 2H).

^{13}C NMR (151 MHz, D_2O)
146.2, 144.4, 110.9, 35.7.
4 resonances are reported instead of the expected 5 due to overlapping peaks

Data were in accordance with those previously reported.[7]

Thiophen-2-ylmethanamine hydrochloride (1w)

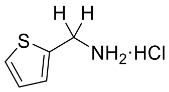

According to a modification of the general procedure, 2-thiophenecarbonitrile (47 µL, 0.50 mmol), pinacolborane (0.25 mL, 1.7 mmol), and borane dimethyl sulfide (5 µL, 0.05 mmol) were reacted at 40 °C for 18 hours under an argon atmosphere. The reaction was allowed to cool to room temperature, and hydrogen chloride (2 M in diethyl ether, 4 mL) and methanol (2 mL) were added. The reaction was stirred for 1 hour at room temperature then concentrated in vacuo. The residue was washed with cold ethyl acetate (2 × 10 mL), filtered, and dried in vacuo to give thiophen-2-ylmethanamine hydrochloride (**1w**, 39 mg, 0.26 mmol, 52%) as a white amorphous solid.

^{1}H NMR (600 MHz, DMSO-d_6)
8.36 (br. s, 3H), 7.57 (dd, J = 5.2, 1.3 Hz, 1H), 7.24 (dd, J = 3.5, 1.2 Hz, 1H), 7.07 (dd, J = 5.1, 3.5 Hz, 1H), 4.23 (s, 2H).

^{13}C NMR (151 MHz, DMSO-d_6)
135.3, 129.2, 127.5, 127.4, 36.9.

Data were in accordance with those previously reported.[16]

S5 Stoichiometric reduction of methyl 4-cyanobenzoate

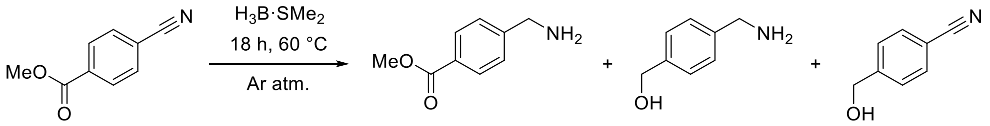

S3 | S4 22% | S5 19% | S6 trace

Borane dimethyl sulfide (0.18 mL, 18 mmol) was added to a stirred solution of methyl 4-cyanobenzoate (81 mg, 0.50 mmol) in THF (3.6 mL) under an argon atmosphere. The reaction mixture was heated at 60 °C for 18 hours. The reaction was allowed to cool to room temperature, then quenched with water (1 mL), followed by a solution of 1,3,5-trimethoxybenzene in diethyl ether (0.10 M, 0.50 mL, 0.050 mmol) and the yield was determined by ^{1}H NMR spectroscopy, using 1,3,5-trimethoxybenzene as an internal standard.

S6 Kinetic analysis

S6.1 Experimental details

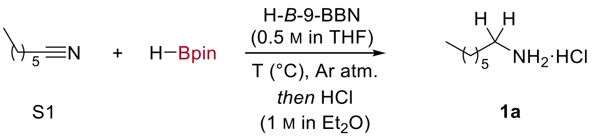

9-Borabicyclo[3.3.1]nonane (0.5 M in THF, 0.20 mL, 0.1 mmol) was added to a stirred solution of heptanenitrile (137 µL, 1.0 mmol) in pinacol borane (0.35 mL, 2.4 mmol) under an argon atmosphere at various temperatures. Aliquots were taken at given time intervals and quenched in hydrogen chloride (1 M in Et_2O). The volatiles were removed and the residue was dissolved in methanol-d_4.

S6.2 Reaction profile over varied temperatures and Arrhenius plot

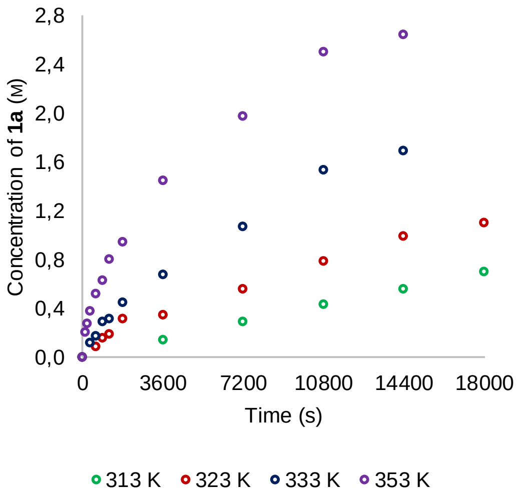

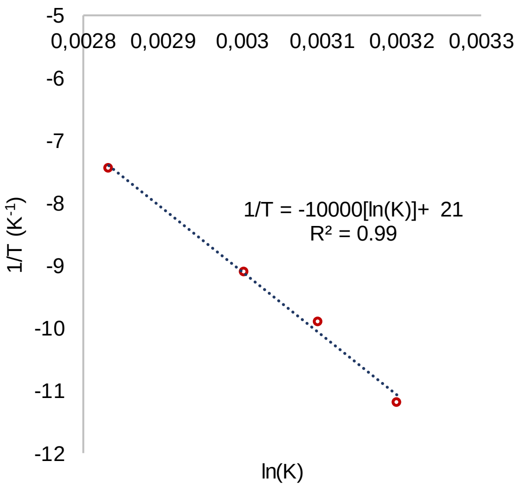

Concentration of **1a** (M) against time (s) at given temperatures (K, Left); Arrhenius Plot (Right)

S6.3 Arrhenius calculation

By quenching aliquots of the reactions with HCl over given time periods, the differentiation of **7**, **9**, and **4a** was not possible (see Scheme 3 in main text). Therefore, this calculation of activation energy should be viewed with this in consideration and is not definitive. By Arrhenius analysis, $\Delta G^{\ddagger}$ was calculated to be 20 kcal mol^{-1}.

$$\Delta G^{\ddagger} = \frac{Rm}{4184}\, kcal\, mol^{-1} = 20\, kcal\, mol^{-1}$$

S7 Computational details

Geometry optimisations and harmonic frequency calculations were performed using the Gaussian16 program package[17] employing the M06-2X density functional[18] in combination with the 6-31+G(d,p) basis set using standard convergence criteria. All geometry optimisations were carried out with no symmetry constraints. Stationary points were characterised as minima or 1st order transition states by analysis of the frequency calculations with zero or one imaginary frequency. Single-point calculations were conducted on the optimised geometries using the M06-2X functional in combination with the higher level 6-311++G(d,p) basis set. Unscaled zero-point vibrational, thermal, and entropic corrections were obtained from frequency calculations at the same level of theory, using standard procedures to obtain free energy corrections at standard conditions (T = 298.15 K, p = 1 atm) and combined with the single point energy. Free energy reaction schemes are presented relative to the free energies of the starting material. Pictures of molecular structures were generated with the ChemCraft[19] program.

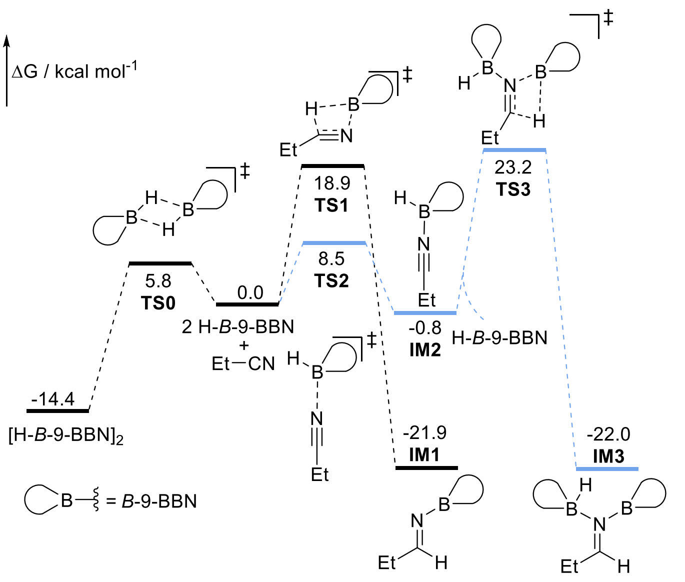

Figure S1. DFT-Computed free energies of nitrile hydroboration.

Black pathway = no pre-coordination of H-*B*-9-BBN. Blue pathway = pre-coordination of H-*B*-9-BBN. (Energies calculated at M06-2X/6-311++G(d,p) on M06-2X/6-31+G(d,p)-optimised structures).

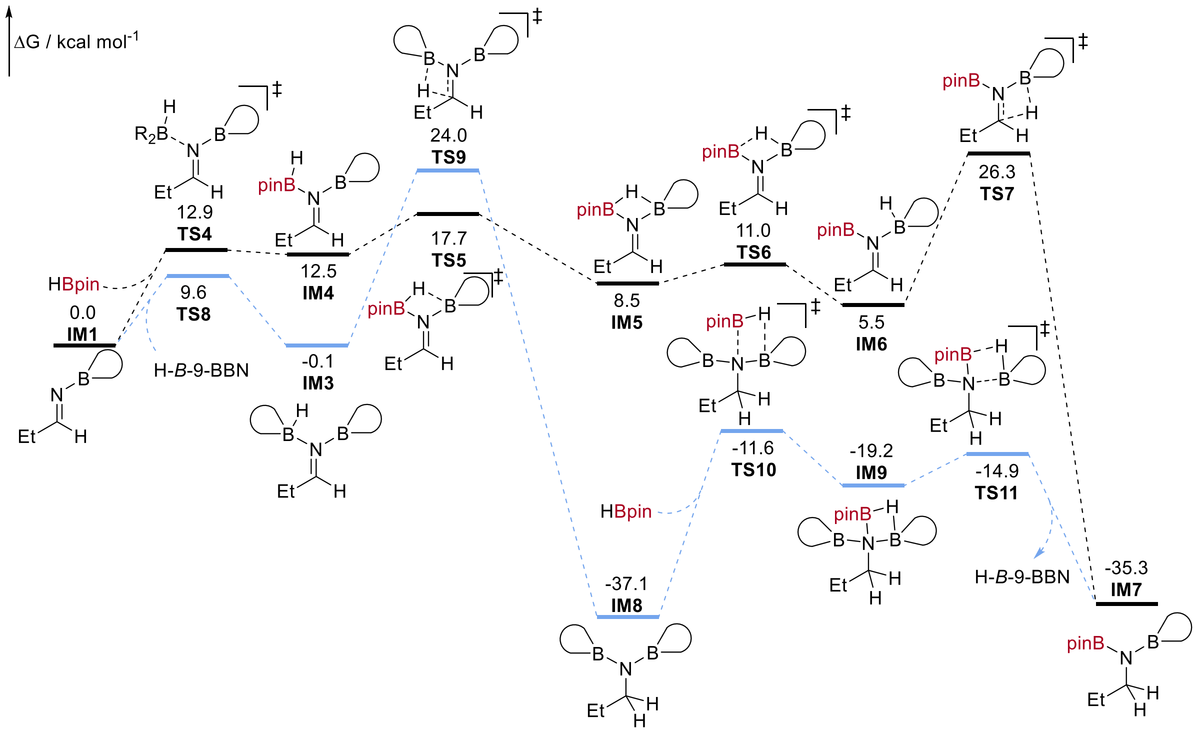

Figure S2. DFT-Computed free energies of imine hydroboration.

Black pathway = coordination of HBpin to imido-*B*-9-BBN IM1. Blue pathway = coordination of H-*B*-9-BBN to IM1. (Energies calculated at M06-2X/6-311++G(d,p) on M06-2X/6-31+G(d,p)-optimised structures).

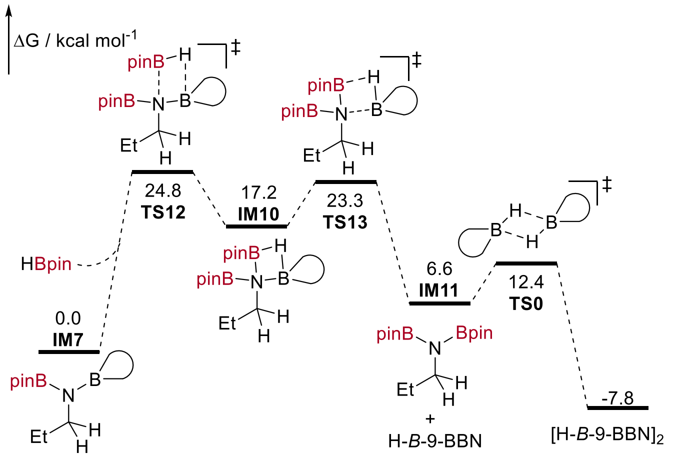

Figure S3. DFT-Computed free energies of transborylation from mixed *bis*-borylamine IM7.

(Energies calculated at M06-2X/6-311++G(d,p) on M06-2X/6-31+G(d,p)-optimised structures).

Cartesian coordinates

HBpin

C	4.807876000	0.808506000	-2.533971000
C	4.546053000	0.858515000	-0.992006000
H	1.498311000	1.568639000	-2.326500000
B	2.636434000	1.305461000	-2.124743000
O	3.100558000	0.841074000	-0.924673000
O	3.618059000	1.435421000	-3.068500000
C	5.001524000	2.168944000	-0.351917000
H	4.608657000	2.215195000	0.666814000
H	6.092626000	2.234204000	-0.310397000
H	4.618921000	3.031232000	-0.906054000
C	5.091299000	-0.327542000	-0.213154000
H	6.179421000	-0.388352000	-0.321449000
H	4.857891000	-0.203960000	0.847519000
H	4.648961000	-1.265237000	-0.553338000
C	4.842660000	-0.615140000	-3.087457000
H	5.749028000	-1.142116000	-2.775537000
H	3.971548000	-1.187889000	-2.755639000
H	4.824136000	-0.566103000	-4.179046000
C	6.027904000	1.586358000	-2.999850000
H	6.936170000	1.185662000	-2.537338000
H	6.125224000	1.493882000	-4.084637000
H	5.943257000	2.645978000	-2.753497000

[H-B-9-BBN]$_2$

C	5.703986000	1.065719000	-4.484687000
C	6.886160000	1.449179000	-3.564544000
C	5.998388000	-0.236529000	-1.766330000
C	4.826898000	-0.599920000	-2.708065000
C	6.593225000	2.616239000	-2.593306000
C	5.715581000	0.949986000	-0.815718000
H	7.719144000	1.757635000	-4.208925000
H	6.205933000	-1.115822000	-1.143469000
B	7.225310000	0.177721000	-2.680597000
C	5.548893000	2.319318000	-1.500744000
C	4.481517000	0.460658000	-3.769860000
H	8.340981000	0.332795000	-1.983989000
H	5.093911000	-1.533938000	-3.223811000
H	3.927107000	-0.824894000	-2.119546000
H	6.076940000	0.333325000	-5.215557000
H	5.382568000	1.939376000	-5.067794000
H	3.887127000	1.257033000	-3.316880000
H	3.827647000	0.002950000	-4.521713000
H	5.610431000	3.102698000	-0.735980000
H	4.543439000	2.402702000	-1.918694000
H	6.555432000	1.014908000	-0.108554000
H	4.823895000	0.741621000	-0.209002000
H	7.540751000	2.885767000	-2.104240000
H	6.279474000	3.505220000	-3.157111000
C	11.190418000	0.098719000	-2.682552000
C	10.018852000	-0.264573000	-3.624228000
C	9.131110000	-1.950297000	-1.826012000
C	10.313366000	-1.566936000	-0.905939000
C	10.301536000	-1.451074000	-4.574897000
C	9.423916000	-3.117343000	-2.797308000
H	9.811318000	0.614750000	-4.247049000

H	8.298146000	-2.258726000	-1.181589000
B	8.791960000	-0.678809000	-2.709919000
C	10.468194000	-2.820439000	-3.889927000
C	11.535819000	-0.961929000	-1.620836000
H	7.676265000	-0.833914000	-3.406486000
H	9.940509000	-0.834540000	-0.175021000
H	10.634771000	-2.440631000	-0.322880000
H	10.923481000	1.032723000	-2.166741000
H	12.090180000	0.323682000	-3.271120000
H	12.130117000	-1.758323000	-2.073904000
H	12.189782000	-0.504299000	-0.869017000
H	10.406567000	-3.603790000	-4.654714000
H	11.473669000	-2.903888000	-3.472042000
H	8.476343000	-3.386805000	-3.286318000
H	9.737659000	-4.006358000	-2.233552000
H	9.461638000	-1.515924000	-5.282012000
H	11.193196000	-1.242737000	-5.181660000

H-*B*-9-BBN

C	5.945802000	1.003259000	-4.397704000
C	7.062175000	1.485767000	-3.446727000
C	6.204403000	-0.177363000	-1.632322000
C	5.091816000	-0.638310000	-2.598380000
C	6.650217000	2.679573000	-2.528348000
C	5.810169000	1.034636000	-0.730014000
H	7.906750000	1.830090000	-4.054345000
H	6.442582000	-1.009000000	-0.959516000
B	7.450102000	0.388153000	-2.397145000
C	5.569667000	2.356921000	-1.480373000
C	4.729480000	0.353875000	-3.716367000
H	8.579903000	0.158635000	-2.072883000
H	5.426623000	-1.575936000	-3.063021000
H	4.185290000	-0.890996000	-2.031299000
H	6.390611000	0.270131000	-5.084662000
H	5.604892000	1.836718000	-5.027239000
H	4.063958000	1.127224000	-3.327297000
H	4.143407000	-0.175363000	-4.477104000
H	5.536811000	3.173290000	-0.749186000
H	4.584617000	2.345600000	-1.950058000
H	6.615375000	1.191006000	0.001825000
H	4.917701000	0.771768000	-0.146539000
H	7.549751000	3.031222000	-2.003610000
H	6.318977000	3.517405000	-3.156435000

TS0

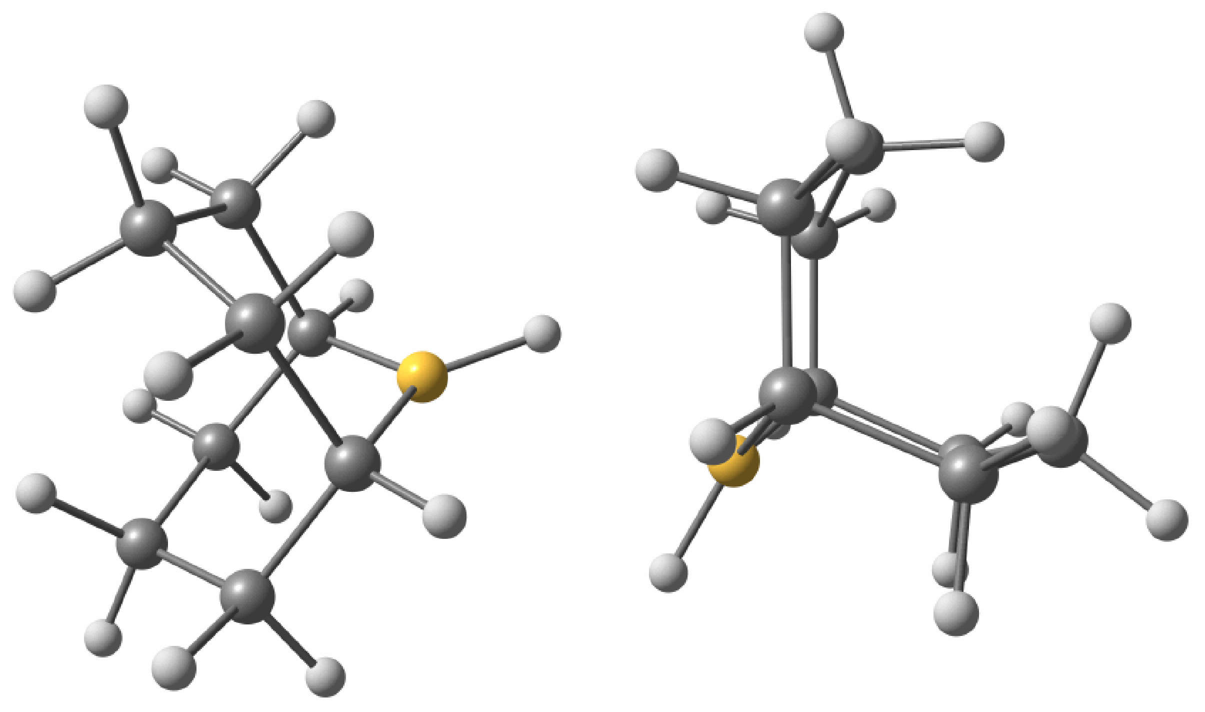

B	-0.699475000	1.004826000	-0.000500000
B	1.747145000	-0.646634000	-0.482595000
H	0.584348000	-0.570135000	-0.782746000
H	0.222485000	1.763739000	0.120427000
C	-1.652746000	1.028928000	-1.248628000
C	-2.748306000	2.032460000	-0.769230000
C	-2.194296000	-0.367318000	-1.615063000
C	-3.430095000	1.662575000	0.560780000
C	-2.726628000	-1.207011000	-0.439501000
C	-1.331725000	0.239146000	1.217383000
C	-2.447231000	1.233960000	1.666891000
C	-1.852083000	-1.157834000	0.825573000
C	2.798948000	-1.271141000	-1.455798000
C	2.294873000	-0.444506000	0.967981000
C	4.158940000	-0.543013000	-1.412941000
C	2.833342000	-2.739153000	-0.917326000
C	3.623483000	0.340930000	0.952473000
C	2.417556000	-1.911125000	1.489265000

C	4.692317000	-0.199746000	-0.010427000
C	3.194688000	-2.874732000	0.572062000
H	-1.192590000	1.466013000	-2.142723000
H	-3.508076000	2.140060000	-1.554856000
H	-2.281403000	3.021419000	-0.659440000
H	-1.374376000	-0.920447000	-2.092831000
H	-2.982562000	-0.277256000	-2.375538000
H	-3.999873000	2.529478000	0.915949000
H	-4.168786000	0.876655000	0.392692000
H	-3.745618000	-0.899146000	-0.194104000
H	-2.811967000	-2.250460000	-0.765807000
H	-0.651870000	0.124499000	2.070502000
H	-1.957511000	2.133989000	2.064081000
H	-3.005335000	0.795908000	2.505273000
H	-0.975183000	-1.801915000	0.668229000
H	-2.408586000	-1.601608000	1.662904000
H	2.464058000	-1.323524000	-2.497639000
H	1.596235000	0.075482000	1.632043000
H	4.043921000	0.393323000	-1.975225000
H	4.914054000	-1.129041000	-1.954681000
H	1.843741000	-3.190326000	-1.081351000
H	3.536481000	-3.323542000	-1.525080000
H	4.041174000	0.385591000	1.967372000
H	3.391052000	1.378475000	0.674798000
H	1.400186000	-2.304609000	1.625768000
H	2.874425000	-1.899223000	2.487927000
H	5.484435000	0.551216000	-0.113281000
H	5.178159000	-1.072296000	0.431692000
H	4.269314000	-2.736954000	0.704169000
H	2.988622000	-3.903493000	0.889924000

EtCN

C	-5.953755000	1.876667000	-0.021267000
C	-4.423920000	1.966661000	-0.067770000
H	-6.288903000	1.482888000	0.940190000
H	-6.326744000	1.220795000	-0.810249000
H	-6.386306000	2.869585000	-0.160628000
C	-3.793131000	0.653796000	0.114961000
H	-4.048607000	2.631358000	0.715605000
H	-4.086267000	2.371420000	-1.026211000
N	-3.315278000	-0.388881000	0.259628000

TS1

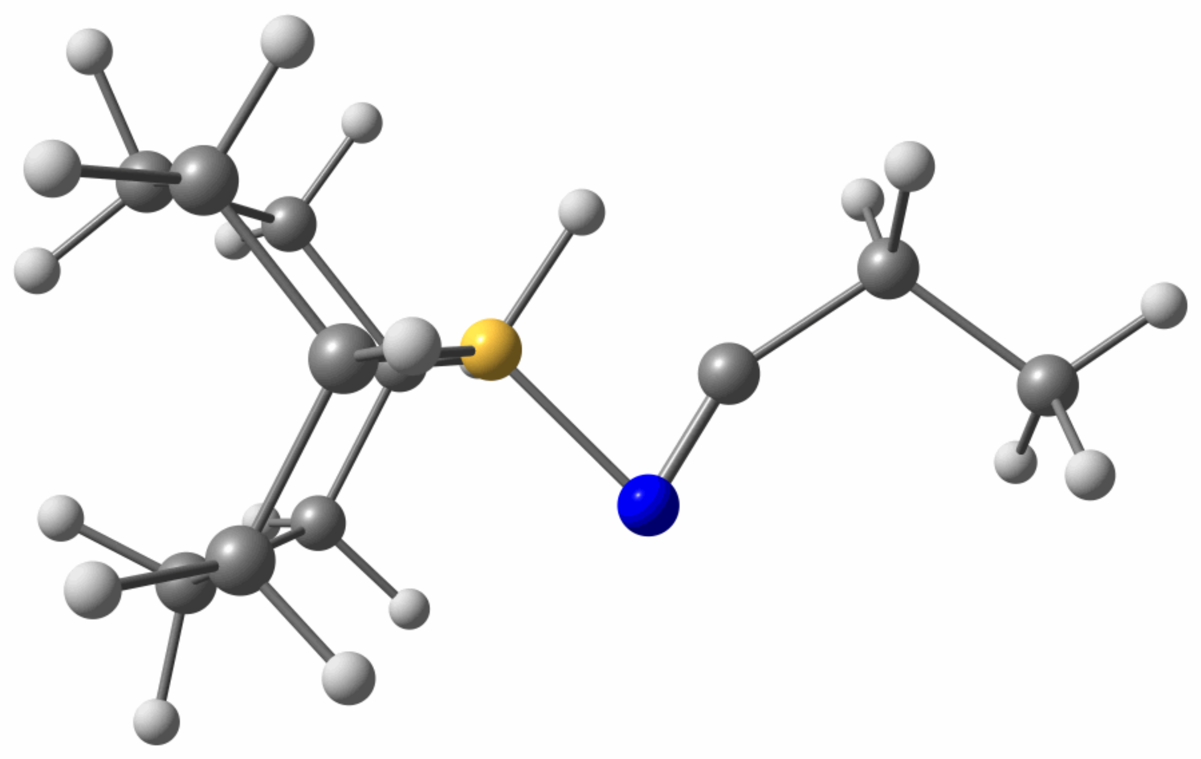

C	5.662667000	-1.794073000	-7.493567000
C	6.631232000	-1.162142000	-6.471005000
C	5.737502000	-2.823451000	-4.636300000
C	4.778305000	-3.435331000	-5.679823000
C	6.034984000	0.016330000	-5.670175000
C	5.154369000	-1.623442000	-3.858212000
H	7.499258000	-0.779850000	-7.027670000
H	5.977658000	-3.608609000	-3.904674000
B	7.023553000	-2.293338000	-5.417256000
C	4.897595000	-0.357221000	-4.699074000
C	4.451609000	-2.530416000	-6.885270000
H	7.912030000	-1.876941000	-4.607568000
H	5.244542000	-4.355601000	-6.055364000
H	3.836757000	-3.738537000	-5.200088000
H	6.238053000	-2.512051000	-8.092542000
H	5.296183000	-1.030008000	-8.193895000
H	3.683806000	-1.805674000	-6.603760000
H	3.992363000	-3.147372000	-7.667357000

H	4.728855000	0.486037000	-4.017924000
H	3.963744000	-0.469442000	-5.254591000
H	5.866038000	-1.369013000	-3.059355000
H	4.218868000	-1.909275000	-3.356822000
H	6.851575000	0.467976000	-5.088296000
H	5.673639000	0.799443000	-6.351648000
H	10.393998000	-2.116726000	-4.898497000
H	11.051254000	-4.081546000	-6.364805000
N	7.853186000	-3.581040000	-6.179395000
C	8.659509000	-3.159280000	-5.409850000
C	9.976028000	-3.115770000	-4.740970000
C	10.893217000	-4.210434000	-5.292260000
H	11.861038000	-4.162457000	-4.789372000
H	9.805552000	-3.230015000	-3.666077000
H	10.462265000	-5.199480000	-5.125006000

IM1

C	-3.986590000	-2.356740000	1.915366000
C	-2.492586000	-2.308460000	1.519103000
C	-2.301957000	0.055810000	2.642381000
C	-3.798454000	-0.024694000	3.023716000
C	-2.212334000	-1.658301000	0.144192000
C	-2.024780000	0.674305000	1.252216000
H	-2.118173000	-3.339028000	1.487489000
H	-1.794762000	0.673451000	3.393828000
B	-1.747438000	-1.429573000	2.608680000
C	-2.521295000	-0.152297000	0.051163000
C	-4.645701000	-0.988884000	2.172513000
H	-3.855936000	-0.346689000	4.072980000
H	-4.249866000	0.976785000	2.986765000
H	-4.066757000	-2.958111000	2.831792000
H	-4.561674000	-2.892930000	1.147326000
H	-4.901107000	-0.516357000	1.221668000
H	-5.603656000	-1.152947000	2.680637000
H	-2.057899000	0.243654000	-0.860596000
H	-3.594787000	-0.002329000	-0.082496000
H	-0.937261000	0.803726000	1.158239000
H	-2.457969000	1.682957000	1.196545000
H	-1.147250000	-1.808493000	-0.082219000
H	-2.768984000	-2.187443000	-0.642106000
N	-0.746120000	-1.893363000	3.414608000
C	0.171443000	-2.318202000	4.148420000
H	-0.073864000	-2.758977000	5.127027000
C	1.639628000	-2.258914000	3.809477000
H	1.752740000	-1.812416000	2.817946000
H	2.023394000	-3.286093000	3.762937000
C	2.420940000	-1.471215000	4.866068000
H	2.082655000	-0.432071000	4.904563000

H	3.490274000	-1.474098000	4.641616000
H	2.285698000	-1.907445000	5.860836000

TS2

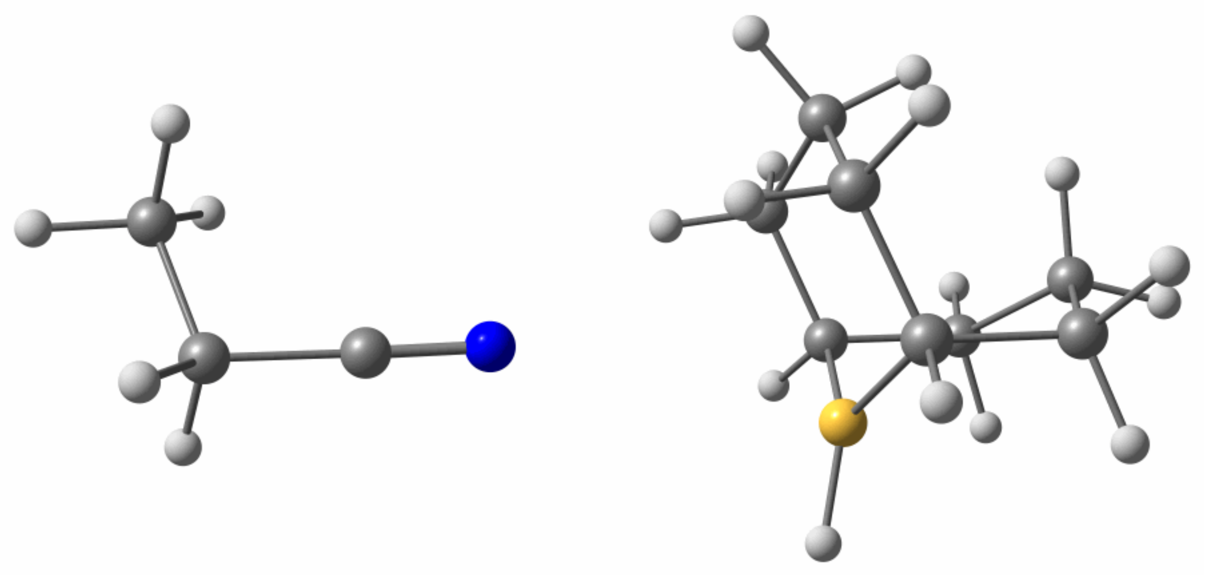

N	-0.954205000	4.686818000	0.669561000
C	-1.251650000	5.721998000	0.250038000
H	-0.676248000	0.952053000	-0.016689000
C	-1.610619000	7.047517000	-0.267784000
H	-2.670873000	7.213817000	-0.056819000
H	-1.495089000	7.024594000	-1.355189000
C	-0.748634000	8.154476000	0.351401000
H	-0.873534000	8.181996000	1.435579000
H	-1.044107000	9.122864000	-0.057319000
H	0.308046000	7.991771000	0.130632000
C	-0.249735000	-0.328584000	2.879063000
C	-0.804517000	1.055146000	2.417316000
C	1.414322000	1.486411000	1.119521000
C	1.941240000	0.093917000	1.588071000
C	-0.463881000	2.224982000	3.364256000
C	1.740410000	2.646159000	2.083774000
H	-1.895378000	0.959890000	2.367678000
H	1.889203000	1.697819000	0.154425000
B	-0.128437000	1.225600000	1.012716000
C	1.026234000	2.599655000	3.446081000
C	1.284754000	-0.453457000	2.868379000

H	1.773336000	-0.627372000	0.775320000
H	3.029811000	0.148151000	1.725358000
H	-0.665472000	-1.101810000	2.217156000
H	-0.631564000	-0.552593000	3.884482000
H	1.705334000	0.044747000	3.743732000
H	1.551726000	-1.511897000	2.975015000
H	1.110287000	3.587395000	3.915600000
H	1.547699000	1.915209000	4.119603000
H	1.454173000	3.576988000	1.579053000
H	2.826160000	2.701065000	2.248129000
H	-1.019141000	3.101817000	3.009575000
H	-0.836176000	2.007258000	4.375560000

IM2

N	-0.725884000	3.440537000	0.455630000
C	-1.020062000	4.522870000	0.209122000
H	-0.684313000	1.278314000	-0.028317000
C	-1.351608000	5.924155000	-0.051768000
H	-2.426777000	6.046468000	0.107340000
H	-1.148301000	6.124551000	-1.107550000
C	-0.542522000	6.857475000	0.860676000
H	-0.746712000	6.643914000	1.911592000
H	-0.818492000	7.892868000	0.653533000
H	0.528103000	6.738778000	0.684892000
C	-0.475289000	0.213708000	2.657515000
C	-0.896881000	1.660241000	2.329033000
C	1.342061000	2.000121000	1.030208000
C	1.747407000	0.551423000	1.368898000
C	-0.440716000	2.702632000	3.367895000
C	1.780804000	3.040113000	2.078931000
H	-1.996925000	1.675285000	2.312847000
H	1.842608000	2.258387000	0.084959000
B	-0.273618000	1.988980000	0.866293000
C	1.079994000	2.934884000	3.446479000
C	1.038783000	-0.062908000	2.591239000
H	1.515649000	-0.065083000	0.489923000
H	2.834927000	0.481260000	1.519595000
H	-0.978105000	-0.443509000	1.935248000
H	-0.843954000	-0.078149000	3.652091000
H	1.518115000	0.285656000	3.509812000
H	1.196519000	-1.148694000	2.578838000
H	1.270286000	3.854639000	4.015181000
H	1.540968000	2.135708000	4.031565000
H	1.582624000	4.042772000	1.666035000
H	2.868779000	2.997222000	2.235325000

H	-0.924160000	3.661681000	3.120467000
H	-0.809833000	2.438104000	4.369750000

TS3

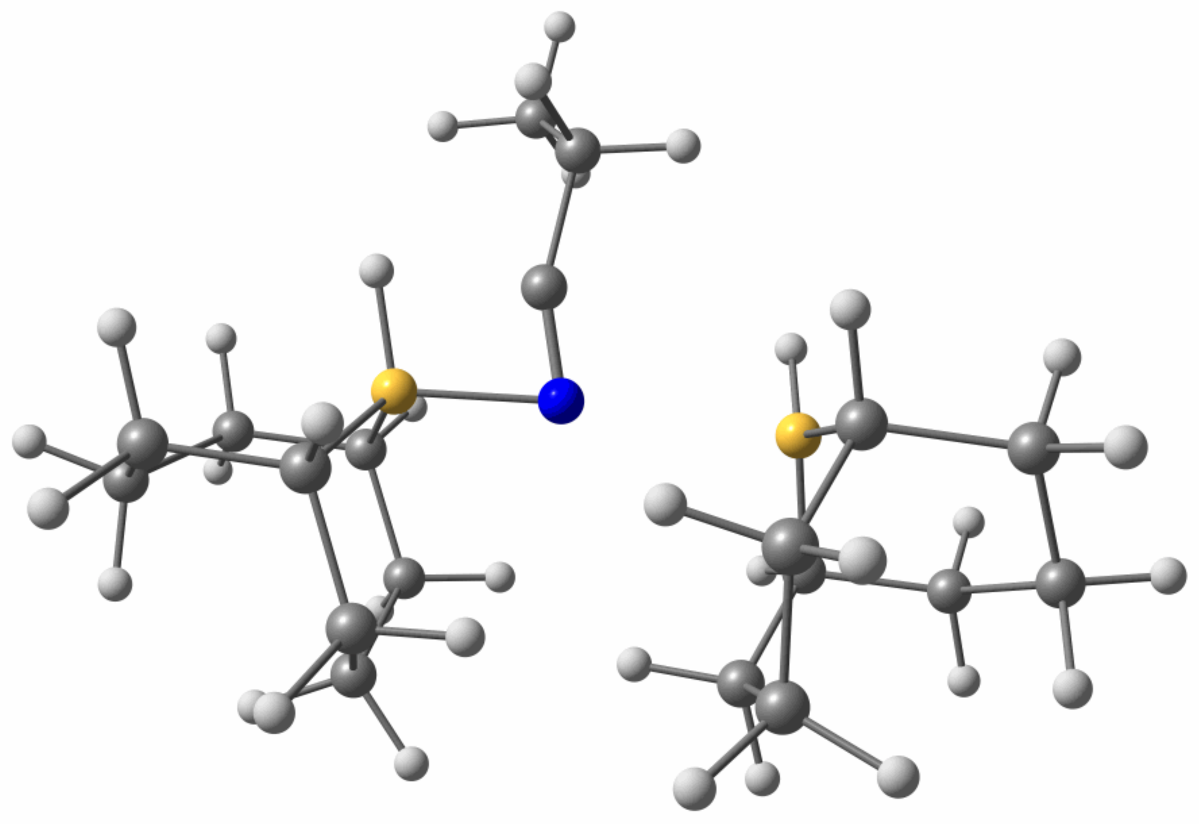

N	-0.324694000	2.710536000	0.094386000
C	-0.671575000	3.271966000	-0.893763000
H	-0.672831000	2.024248000	-2.117288000
C	-1.055713000	4.404511000	-1.753101000
H	-1.164609000	5.256850000	-1.072969000
H	-2.032998000	4.181874000	-2.190657000
C	-0.004546000	4.673387000	-2.832993000
H	0.959101000	4.916873000	-2.380662000
H	-0.323442000	5.514256000	-3.451287000
H	0.122255000	3.797589000	-3.474246000
H	0.265233000	5.189682000	1.070633000
B	-0.180190000	4.434909000	1.893902000
H	-2.365348000	4.823085000	1.506230000
H	1.771975000	3.658595000	2.677750000
H	-2.306888000	2.446651000	1.811947000
H	0.428603000	1.684646000	2.588981000

C	-1.700228000	4.444342000	2.294914000
C	0.724347000	3.763634000	2.984438000
H	1.200099000	5.737584000	3.753842000
H	-1.469191000	6.485049000	2.996438000
C	-2.206010000	3.052611000	2.722529000
C	0.202215000	2.377719000	3.408735000
C	0.686838000	4.835967000	4.117278000
C	-1.711485000	5.507193000	3.436753000
H	-3.215781000	3.134544000	3.148914000
H	0.763069000	2.015225000	4.281801000
C	-1.304594000	2.294176000	3.714438000
H	1.271551000	4.477128000	4.974810000
H	-2.729476000	5.597443000	3.839013000
C	-0.724902000	5.233755000	4.587653000
H	-1.600810000	1.237561000	3.714836000
H	-0.648605000	6.136596000	5.205342000
H	-1.497661000	2.645114000	4.731133000
H	-1.127944000	4.461717000	5.245633000
C	-1.276979000	-0.774638000	-1.777947000
C	-1.292080000	0.271772000	-0.642061000
C	1.205064000	0.881906000	-1.215355000
C	1.184785000	-0.183640000	-2.333848000
C	-0.832712000	-0.271527000	0.727423000
C	1.643506000	0.352556000	0.166064000
H	-2.327198000	0.626129000	-0.529069000
H	1.918758000	1.663569000	-1.514857000
B	-0.285029000	1.429085000	-1.074417000
C	0.664462000	-0.634407000	0.837706000
C	0.122499000	-1.286946000	-2.167276000
H	0.996530000	0.336076000	-3.284887000
H	2.173003000	-0.654553000	-2.431107000
H	-1.737545000	-0.314470000	-2.664167000

H	-1.908150000	-1.634506000	-1.513536000
H	0.463286000	-2.016686000	-1.428944000
H	0.043499000	-1.842456000	-3.109823000
H	0.923304000	-0.708116000	1.901923000
H	0.827364000	-1.636990000	0.434270000
H	1.771745000	1.223163000	0.823575000
H	2.630657000	-0.125866000	0.096600000
H	-1.059768000	0.499302000	1.475277000
H	-1.428612000	-1.152198000	1.006573000

IM3

N	-0.621899000	2.803364000	-0.054511000
C	-1.179645000	3.144233000	-1.157944000
H	-1.383584000	2.364947000	-1.900683000
C	-1.489279000	4.537710000	-1.599634000
H	-1.448706000	5.228444000	-0.760167000
H	-2.502296000	4.545792000	-2.017978000
C	-0.476091000	4.946859000	-2.681444000
H	0.531987000	4.974548000	-2.258980000
H	-0.715201000	5.940352000	-3.066836000
H	-0.481093000	4.246708000	-3.523123000
H	0.269651000	4.827473000	0.538354000
B	-0.286420000	3.909306000	1.117703000
H	-2.419802000	4.727401000	1.082877000
H	1.607182000	2.936054000	1.946630000
H	-2.670419000	2.418081000	1.652784000
H	-0.088420000	1.262730000	2.261801000
C	-1.697908000	4.330927000	1.815177000
C	0.629507000	3.292854000	2.305737000
H	1.540332000	5.186774000	2.722254000
H	-1.076672000	6.343680000	2.202269000
C	-2.375602000	3.106641000	2.460922000
C	-0.062586000	2.097228000	2.982990000
C	0.932979000	4.456445000	3.273247000
C	-1.397626000	5.478634000	2.799270000
H	-3.312091000	3.397134000	2.959426000
H	0.539795000	1.709747000	3.819499000
C	-1.504850000	2.329000000	3.461944000
H	1.552514000	4.107608000	4.112892000
H	-2.312159000	5.788016000	3.327451000
C	-0.301793000	5.180566000	3.840093000
H	-1.972975000	1.356134000	3.665139000

H	0.021725000	6.126602000	4.292072000
H	-1.491682000	2.848415000	4.422903000
H	-0.723383000	4.599462000	4.664628000
C	-1.015152000	-0.390401000	-1.471799000
C	-1.144272000	0.138724000	-0.008097000
C	1.324275000	1.007108000	-0.112434000
C	1.361933000	0.604955000	-1.618318000
C	-0.723100000	-0.925584000	1.031492000
C	1.758568000	-0.116493000	0.853662000
H	-2.197055000	0.396836000	0.162127000
H	1.995271000	1.863558000	0.012192000
B	-0.183513000	1.381552000	0.072394000
C	0.773782000	-1.296209000	1.014218000
C	0.425972000	-0.554951000	-1.992049000
H	1.104080000	1.488483000	-2.222952000
H	2.392677000	0.348035000	-1.892940000
H	-1.554596000	0.290426000	-2.143074000
H	-1.542908000	-1.350492000	-1.543685000
H	0.851026000	-1.492416000	-1.629088000
H	0.395434000	-0.649292000	-3.083747000
H	1.010738000	-1.817142000	1.948699000
H	0.948762000	-2.031102000	0.225014000
H	1.919116000	0.347725000	1.834201000
H	2.734891000	-0.511665000	0.542513000
H	-0.999029000	-0.561370000	2.027878000
H	-1.313499000	-1.837243000	0.870410000

TS4

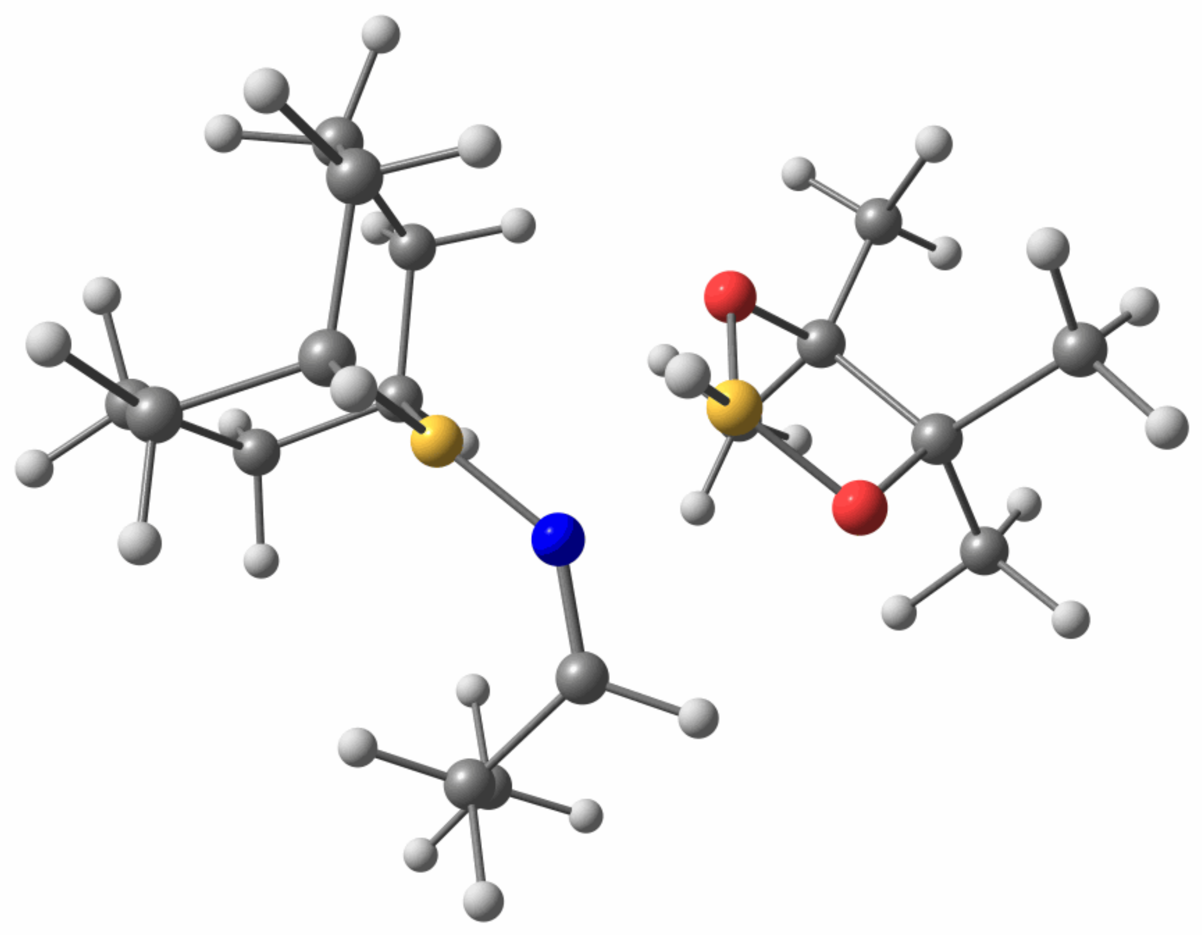

C	-1.361784000	-0.656063000	-0.254705000
C	-0.272116000	-1.455211000	0.502440000
C	0.007556000	0.563620000	2.145463000
C	-1.026216000	1.365232000	1.317093000
C	1.174389000	-1.226336000	-0.007092000
C	1.476207000	0.717902000	1.666903000
H	-0.502737000	-2.522248000	0.404265000
H	-0.043959000	0.910847000	3.184849000
B	-0.309774000	-0.970226000	1.996738000
C	1.760477000	0.173363000	0.256941000
C	-1.252306000	0.872354000	-0.124779000
H	-1.985003000	1.298038000	1.845446000
H	-0.741848000	2.426612000	1.300701000
H	-2.337815000	-0.970089000	0.137864000
H	-1.346845000	-0.932780000	-1.317669000

H	-0.465297000	1.246769000	-0.783465000
H	-2.181614000	1.318624000	-0.497200000
H	2.846161000	0.131981000	0.109013000
H	1.391876000	0.880640000	-0.488093000
H	2.128713000	0.195675000	2.384223000
H	1.766286000	1.776104000	1.714451000
H	1.822060000	-1.969504000	0.480610000
H	1.224434000	-1.444573000	-1.082407000
N	-0.648011000	-1.836408000	3.087466000
C	-0.040218000	-2.461765000	4.003606000
H	-0.654588000	-3.011415000	4.726390000
C	1.449679000	-2.429666000	4.207793000
H	1.944805000	-2.213782000	3.254376000
H	1.794787000	-3.409295000	4.554417000
C	1.803712000	-1.353356000	5.244821000
H	1.497504000	-0.364706000	4.888578000
H	2.879996000	-1.334449000	5.432498000
H	1.295691000	-1.542198000	6.195499000
C	-3.608578000	-0.456511000	4.112019000
C	-3.859426000	-1.821079000	4.838219000
H	-2.525085000	-2.859336000	1.968917000
B	-2.621088000	-2.161916000	2.937908000
O	-2.918207000	-2.698293000	4.203358000
O	-3.187665000	-0.879035000	2.806534000
C	-5.255689000	-2.382781000	4.562801000
H	-5.292371000	-3.414021000	4.923660000
H	-6.033618000	-1.804793000	5.071236000
H	-5.463530000	-2.389008000	3.488793000
C	-3.591267000	-1.804037000	6.336592000
H	-4.241784000	-1.080421000	6.840050000
H	-3.794650000	-2.794870000	6.752483000
H	-2.550614000	-1.550204000	6.551236000

C	-2.487432000	0.358090000	4.763331000
H	-2.802979000	0.759215000	5.731597000
H	-1.586455000	-0.243516000	4.910187000
H	-2.235869000	1.197904000	4.109233000
C	-4.846095000	0.417809000	3.960589000
H	-5.265261000	0.671811000	4.940497000
H	-4.573611000	1.347896000	3.452881000
H	-5.611430000	-0.082843000	3.365292000

IM4

C	-1.432226000	-0.639846000	-0.202590000
C	-0.345417000	-1.478948000	0.519972000
C	0.028592000	0.497693000	2.193446000
C	-1.022461000	1.334272000	1.413958000
C	1.098100000	-1.274166000	-0.011312000
C	1.478087000	0.657977000	1.663246000
H	-0.605315000	-2.537398000	0.408906000
H	0.009115000	0.824778000	3.240310000
B	-0.356591000	-1.011583000	2.013105000
C	1.713327000	0.113934000	0.243762000
C	-1.293269000	0.882675000	-0.033715000
H	-1.965158000	1.265619000	1.967776000
H	-0.716884000	2.389497000	1.418369000
H	-2.406003000	-0.949550000	0.197020000
H	-1.432207000	-0.891312000	-1.271660000
H	-0.518629000	1.264128000	-0.702888000
H	-2.225270000	1.352815000	-0.367617000
H	2.793394000	0.056089000	0.064425000
H	1.334322000	0.828525000	-0.488595000
H	2.162045000	0.146503000	2.357290000
H	1.759224000	1.718819000	1.698115000
H	1.740881000	-2.032025000	0.460390000
H	1.123303000	-1.490394000	-1.087551000
N	-0.735296000	-1.912814000	3.112569000
C	-0.016773000	-2.468708000	4.001796000
H	-0.546404000	-3.055459000	4.757996000
C	1.471060000	-2.314292000	4.106768000
H	1.885993000	-2.020460000	3.135874000
H	1.912851000	-3.279001000	4.377170000
C	1.818130000	-1.265806000	5.174578000
H	1.424434000	-0.284891000	4.892345000

H	2.901093000	-1.182630000	5.291719000
H	1.389845000	-1.536701000	6.144017000
C	-3.524773000	-0.453613000	4.121016000
C	-3.836209000	-1.810175000	4.834616000
H	-2.516086000	-2.940038000	2.107550000
B	-2.417425000	-2.186658000	3.052482000
O	-2.797289000	-2.662285000	4.353670000
O	-3.023566000	-0.887869000	2.855492000
C	-5.176585000	-2.399713000	4.386357000
H	-5.237458000	-3.431435000	4.743044000
H	-6.025093000	-1.838050000	4.790295000
H	-5.244098000	-2.409336000	3.294436000
C	-3.773200000	-1.757953000	6.355315000
H	-4.492320000	-1.032330000	6.751743000
H	-4.019923000	-2.742362000	6.763353000
H	-2.772437000	-1.487435000	6.700020000
C	-2.445061000	0.347449000	4.858938000
H	-2.826368000	0.760065000	5.798493000
H	-1.571550000	-0.273234000	5.082218000
H	-2.124809000	1.180172000	4.225561000
C	-4.740638000	0.432889000	3.884851000
H	-5.231330000	0.685177000	4.831698000
H	-4.427087000	1.364614000	3.403441000
H	-5.461089000	-0.061470000	3.230864000

TS5

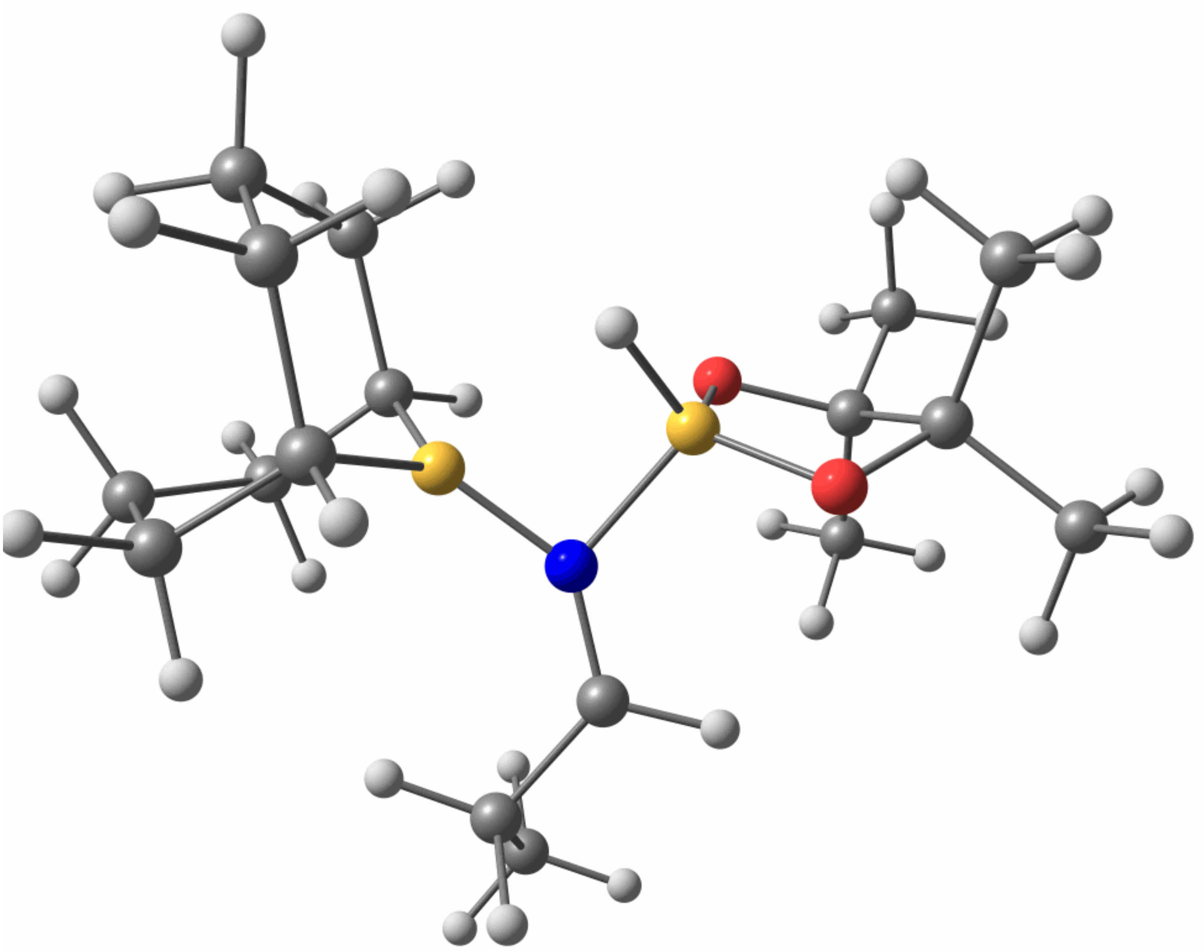

C	-1.237947000	-0.919468000	-0.420764000
C	-0.179205000	-1.510102000	0.538216000
C	-0.680235000	0.426449000	2.236745000
C	-1.684968000	1.029665000	1.231996000
C	1.248028000	-0.934164000	0.314673000
C	0.786735000	0.911667000	2.070487000
H	-0.134752000	-2.594551000	0.379729000
H	-1.013618000	0.674318000	3.249753000
B	-0.568799000	-1.119816000	2.010678000
C	1.445525000	0.537335000	0.731224000
C	-1.520130000	0.582455000	-0.233771000
H	-2.684848000	0.740707000	1.578722000
H	-1.644290000	2.126397000	1.279455000
H	-2.170082000	-1.476962000	-0.269906000

H	-0.930931000	-1.100272000	-1.459708000
H	-0.733489000	1.165767000	-0.718487000
H	-2.439470000	0.831162000	-0.776498000
H	2.521088000	0.740264000	0.797217000
H	1.072065000	1.198759000	-0.052505000
H	1.384767000	0.487448000	2.892614000
H	0.828316000	2.000491000	2.204210000
H	1.961370000	-1.555513000	0.873059000
H	1.519941000	-1.051601000	-0.742861000
N	-0.607401000	-2.065641000	3.143441000
C	0.179513000	-2.780426000	3.831772000
H	-0.287995000	-3.492374000	4.519783000
C	1.672506000	-2.692366000	3.813020000
H	1.995394000	-2.018944000	3.013740000
H	2.079394000	-3.688645000	3.603605000
C	2.185925000	-2.200918000	5.174273000
H	1.808185000	-1.197524000	5.389501000
H	3.277514000	-2.168079000	5.182267000
H	1.860163000	-2.865301000	5.979999000
C	-3.377590000	-1.836503000	5.216189000
C	-3.715936000	-3.227244000	4.581136000
H	-2.499531000	-1.793375000	2.029594000
B	-2.252475000	-2.114557000	3.208123000
O	-2.636613000	-3.419711000	3.666330000
O	-2.777873000	-1.138479000	4.128199000
C	-5.020543000	-3.192594000	3.780125000
H	-5.092042000	-4.113452000	3.194918000
H	-5.897606000	-3.121197000	4.431012000
H	-5.025246000	-2.344595000	3.088379000
C	-3.738892000	-4.386389000	5.567271000
H	-4.487307000	-4.217073000	6.349401000
H	-3.995023000	-5.310067000	5.040482000

H	-2.762526000	-4.522622000	6.038250000
C	-2.364267000	-1.941337000	6.361180000
H	-2.812784000	-2.368321000	7.264227000
H	-1.506959000	-2.558571000	6.078647000
H	-1.999566000	-0.936398000	6.592716000
C	-4.593597000	-1.050648000	5.692542000
H	-5.152537000	-1.614155000	6.448380000
H	-4.266433000	-0.107470000	6.140000000
H	-5.258401000	-0.817517000	4.858908000

IM5

C	-0.298398000	0.447053000	-3.171323000
C	0.707194000	-0.226298000	-2.215462000
C	0.852391000	2.009956000	-0.847062000
C	-0.158459000	2.648519000	-1.821538000
C	2.182047000	-0.063724000	-2.633784000
C	2.324679000	2.174392000	-1.275008000
H	0.480513000	-1.300978000	-2.197980000
H	0.730350000	2.502284000	0.127714000
B	0.512217000	0.445593000	-0.772981000
C	2.758162000	1.365357000	-2.518422000
C	-0.231561000	1.984678000	-3.208701000
H	-1.151872000	2.591718000	-1.354262000
H	0.060499000	3.718058000	-1.949627000
H	-1.307999000	0.149414000	-2.853241000
H	-0.171038000	0.060006000	-4.192257000
H	0.622144000	2.301657000	-3.813807000
H	-1.116449000	2.363549000	-3.734501000
H	3.854374000	1.304706000	-2.533072000
H	2.494281000	1.927487000	-3.417323000
H	2.954363000	1.889937000	-0.420965000
H	2.547803000	3.235390000	-1.457634000
H	2.779159000	-0.748143000	-2.016004000
H	2.321697000	-0.410372000	-3.667748000
N	1.000848000	-0.355899000	0.472220000
C	1.992428000	-0.777522000	1.140177000
H	1.773650000	-1.326403000	2.064092000
C	3.437168000	-0.565152000	0.821517000
H	3.542615000	-0.115129000	-0.166470000
H	3.926133000	-1.546793000	0.801326000
C	4.093477000	0.313488000	1.896160000
H	3.636134000	1.306595000	1.914090000

H	5.159949000	0.429555000	1.691507000
H	3.985009000	-0.128725000	2.891017000
C	-2.080016000	-0.604749000	2.379455000
C	-2.384361000	-1.598445000	1.208662000
H	-0.745712000	0.327132000	-0.456821000
B	-0.481381000	-0.476683000	0.757432000
O	-1.094559000	-1.724139000	0.584098000
O	-1.060267000	0.240475000	1.816577000
C	-3.356127000	-1.012055000	0.182395000
H	-3.347596000	-1.647409000	-0.707362000
H	-4.376249000	-0.972215000	0.575858000
H	-3.059582000	-0.001300000	-0.115250000
C	-2.850684000	-2.977246000	1.647611000
H	-3.772949000	-2.903357000	2.233959000
H	-3.052170000	-3.591820000	0.766034000
H	-2.088565000	-3.479077000	2.246572000
C	-1.473588000	-1.307145000	3.594670000
H	-2.217456000	-1.905995000	4.129099000
H	-0.650704000	-1.962687000	3.291972000
H	-1.080551000	-0.547925000	4.276134000
C	-3.259756000	0.257043000	2.803022000
H	-4.092948000	-0.368548000	3.141569000
H	-2.959384000	0.906123000	3.630160000
H	-3.601076000	0.889063000	1.981322000

TS6

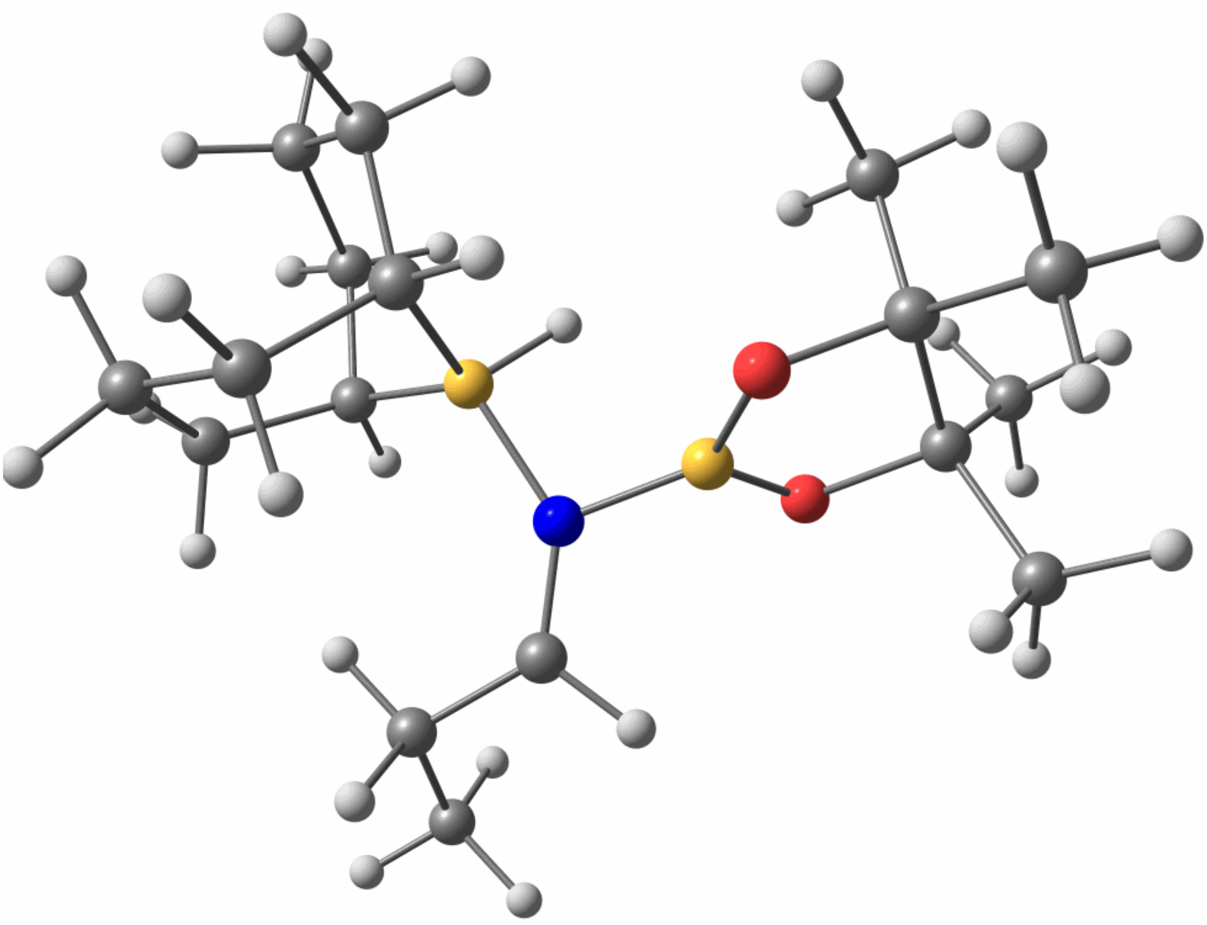

C	-0.457005000	0.337784000	-2.892487000
C	0.439086000	-0.340857000	-1.830909000
C	1.353372000	2.003702000	-1.146679000
C	0.285500000	2.684198000	-2.038161000
C	1.851938000	-0.705574000	-2.315325000
C	2.631187000	1.746487000	-1.990403000
H	-0.062683000	-1.273622000	-1.542696000
H	1.615313000	2.719938000	-0.351839000
B	0.556480000	0.695050000	-0.590538000
C	2.639087000	0.482390000	-2.881494000
C	-0.136797000	1.811567000	-3.234075000
H	-0.593401000	2.919022000	-1.426099000
H	0.667242000	3.640009000	-2.426028000
H	-1.483271000	0.299849000	-2.502378000
H	-0.461470000	-0.245184000	-3.826417000

H	0.650809000	1.857832000	-3.992105000
H	-1.019012000	2.259033000	-3.708879000
H	3.679047000	0.179391000	-3.058176000
H	2.235798000	0.717677000	-3.870369000
H	3.511322000	1.704470000	-1.339527000
H	2.814488000	2.622588000	-2.630782000
H	2.406362000	-1.140222000	-1.466345000
H	1.814228000	-1.494247000	-3.080926000
N	1.020055000	-0.113263000	0.747602000
C	2.083839000	-0.258826000	1.441440000
H	2.008038000	-0.836537000	2.371192000
C	3.425186000	0.308715000	1.138144000
H	3.350645000	0.958624000	0.270729000
H	4.089960000	-0.525674000	0.877379000
C	3.987067000	1.057797000	2.352389000
H	3.352621000	1.910081000	2.609036000
H	4.989011000	1.433106000	2.134247000
H	4.054260000	0.405205000	3.228406000
C	-1.842601000	-1.103289000	2.830838000
C	-2.209474000	-1.731719000	1.443205000
H	-0.565100000	1.028728000	-0.198792000
B	-0.244094000	-0.662547000	1.302936000
O	-0.936992000	-1.691645000	0.747030000
O	-0.750833000	-0.211112000	2.486105000
C	-3.176110000	-0.870783000	0.633294000
H	-3.218695000	-1.262916000	-0.386032000
H	-4.181960000	-0.892358000	1.062530000
H	-2.831283000	0.166256000	0.579759000
C	-2.690135000	-3.171238000	1.503145000
H	-3.602756000	-3.242356000	2.104141000
H	-2.916363000	-3.520186000	0.492564000
H	-1.931720000	-3.828982000	1.931193000

C	-1.278323000	-2.122938000	3.817002000
H	-2.056353000	-2.802529000	4.175941000
H	-0.482637000	-2.717017000	3.356188000
H	-0.859701000	-1.588497000	4.673674000
C	-2.954387000	-0.292022000	3.473643000
H	-3.830173000	-0.923967000	3.654799000
H	-2.611072000	0.103228000	4.433191000
H	-3.247506000	0.547356000	2.841362000

IM6

C	1.441720000	-0.091090000	-0.513306000
C	-0.046579000	-0.559599000	-0.710850000
B	0.041758000	1.669698000	-0.351987000
O	-0.745777000	0.698346000	-0.897496000
O	1.292003000	1.263914000	-0.018122000
C	-0.653077000	-1.217249000	0.526563000
H	-1.725882000	-1.345370000	0.362006000
H	-0.206825000	-2.198469000	0.710995000
H	-0.519622000	-0.597439000	1.417358000
C	-0.278426000	-1.432939000	-1.933622000
H	0.301222000	-2.358333000	-1.853968000
H	-1.337286000	-1.696441000	-1.994904000
H	0.002191000	-0.920152000	-2.855057000
C	2.218266000	0.013833000	-1.823540000
H	2.421750000	-0.973758000	-2.246849000
H	1.668778000	0.605209000	-2.562648000
H	3.169417000	0.513916000	-1.626245000
C	2.225653000	-0.894593000	0.511050000
H	2.288039000	-1.944599000	0.206695000
H	3.240196000	-0.495460000	0.585222000
H	1.763162000	-0.836617000	1.498039000
N	-0.428505000	3.073162000	-0.261812000
C	-0.934135000	3.530864000	-1.350193000
H	-0.997768000	2.855101000	-2.210739000
C	-1.377821000	4.934625000	-1.587538000
H	-1.491951000	5.468225000	-0.646373000
H	-2.345982000	4.899088000	-2.099914000
C	-0.341943000	5.636538000	-2.482628000
H	0.616822000	5.706149000	-1.962687000
H	-0.679046000	6.647504000	-2.720613000
H	-0.191361000	5.098865000	-3.423961000

H	0.153167000	5.040622000	0.713042000
B	-0.315011000	3.982241000	1.099787000
H	-2.533886000	4.520109000	0.985176000
H	1.616791000	3.103908000	1.933642000
H	-2.431085000	2.111831000	1.257522000
H	0.212821000	1.216018000	2.147850000
C	-1.813496000	4.125964000	1.721122000
C	0.596478000	3.330522000	2.274797000
H	1.228430000	5.298363000	2.872173000
H	-1.549397000	6.141038000	2.388123000
C	-2.351396000	2.748976000	2.154481000
C	0.020134000	2.024688000	2.865107000
C	0.715680000	4.445818000	3.336277000
C	-1.734525000	5.165288000	2.858600000
H	-3.374998000	2.836268000	2.547906000
H	0.581278000	1.738025000	3.768253000
C	-1.484863000	2.024929000	3.195371000
H	1.351085000	4.115315000	4.171638000
H	-2.701955000	5.251046000	3.376912000
C	-0.629408000	4.928230000	3.910112000
H	-1.829657000	0.985795000	3.289361000
H	-0.465642000	5.863160000	4.460933000
H	-1.655038000	2.473226000	4.177210000
H	-0.977289000	4.212419000	4.659910000

TS7

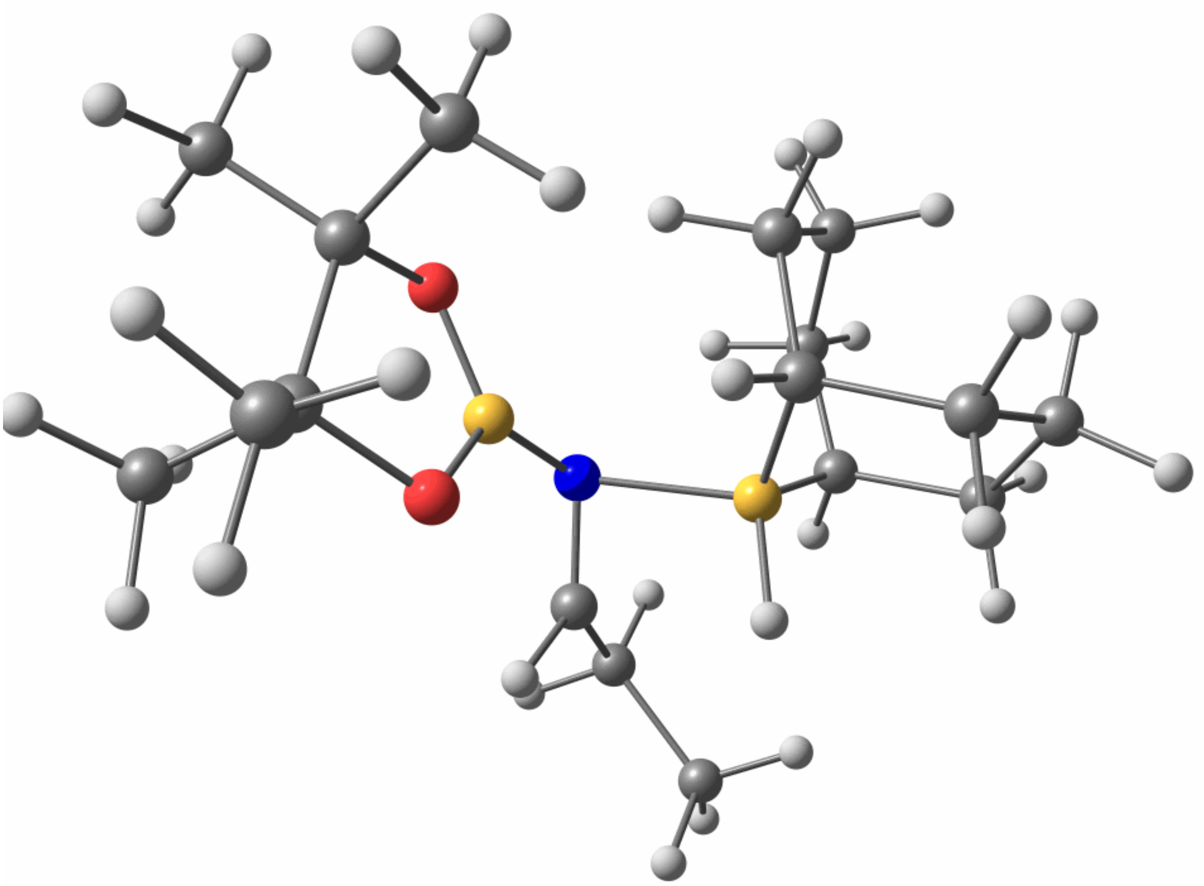

C	0.641338000	-0.793521000	0.184054000
C	0.628116000	-0.382017000	-1.330014000
B	-0.721501000	0.987232000	-0.125540000
O	0.001287000	0.921585000	-1.291296000
O	-0.492528000	-0.067027000	0.710516000
C	-0.269699000	-1.275359000	-2.183730000
H	-0.380154000	-0.819661000	-3.171154000
H	0.160367000	-2.273359000	-2.306670000
H	-1.263743000	-1.376031000	-1.737069000
C	2.002785000	-0.247038000	-1.963475000
H	2.537705000	-1.201692000	-1.922591000
H	1.894667000	0.041672000	-3.012223000
H	2.598455000	0.514892000	-1.457948000
C	1.878071000	-0.299355000	0.932487000
H	2.771850000	-0.850489000	0.625950000

H	2.046783000	0.768380000	0.765251000
H	1.722148000	-0.451005000	2.003739000
C	0.432601000	-2.277855000	0.438661000
H	1.232059000	-2.861375000	-0.029966000
H	0.454854000	-2.467257000	1.514795000
H	-0.528920000	-2.619010000	0.051609000
N	-1.730646000	2.016984000	0.154602000
C	-2.234387000	2.587664000	-0.902805000
H	-1.809198000	2.387468000	-1.898535000
C	-3.515146000	3.357901000	-0.899874000
H	-3.891988000	3.437875000	0.120739000
H	-4.208346000	2.702708000	-1.450256000
C	-3.440000000	4.712133000	-1.605667000
H	-2.851380000	5.417056000	-1.014011000
H	-4.442012000	5.122565000	-1.747599000
H	-2.966863000	4.619358000	-2.588427000
H	-0.899303000	4.203132000	-0.555303000
B	-0.996094000	3.668484000	0.547003000
H	-2.858600000	4.735258000	1.313894000
H	1.076986000	2.721523000	0.480196000
H	-2.555865000	2.786245000	2.725558000
H	0.022624000	1.580969000	2.331773000
C	-1.838557000	4.483801000	1.634030000
C	0.461118000	3.335223000	1.154792000
H	1.301938000	5.081803000	0.218340000
H	-1.246960000	6.388781000	0.830467000
C	-1.955106000	3.680234000	2.940873000
C	0.355647000	2.608708000	2.512924000
C	1.165302000	4.709826000	1.242108000
C	-1.104839000	5.842588000	1.774191000
H	-2.504841000	4.255945000	3.699338000
H	1.355263000	2.528824000	2.967803000

C	-0.610834000	3.232324000	3.537986000
H	2.171479000	4.586350000	1.668016000
H	-1.584577000	6.450030000	2.554903000
C	0.408289000	5.771385000	2.060092000
H	-0.803503000	2.491985000	4.324211000
H	0.849433000	6.754338000	1.853281000
H	-0.135855000	4.075590000	4.045328000
H	0.577060000	5.598136000	3.125227000

IM7

C	-0.765566000	-1.210122000	-1.064269000
C	0.237886000	-1.178408000	0.113776000
C	-0.317987000	1.334354000	0.519135000
C	-1.321120000	1.273699000	-0.661047000
C	1.679589000	-0.772899000	-0.286049000
C	1.125230000	1.713132000	0.109064000
H	0.301055000	-2.199920000	0.509032000
H	-0.674619000	2.105071000	1.204984000
B	-0.276609000	-0.108486000	1.168576000
C	1.855186000	0.678554000	-0.763631000
C	-1.079309000	0.158281000	-1.691909000
N	-0.657656000	-0.434154000	2.509393000
H	-2.325329000	1.138022000	-0.233922000
H	-1.341345000	2.243675000	-1.177416000
H	-1.704656000	-1.643560000	-0.689490000
H	-0.405412000	-1.894858000	-1.844858000
H	-0.274581000	0.446855000	-2.371501000
H	-1.971495000	0.060386000	-2.322337000
H	2.925898000	0.916234000	-0.775882000
H	1.526304000	0.769544000	-1.801108000
H	1.702387000	1.860480000	1.033762000
H	1.124446000	2.684096000	-0.405937000
H	2.323156000	-0.925849000	0.592988000
H	2.055421000	-1.457450000	-1.059388000
C	-0.671821000	-1.844629000	2.943723000
B	-0.925141000	0.519831000	3.550168000
O	-0.946127000	1.892655000	3.421684000
O	-1.175572000	0.118297000	4.849993000
C	0.698343000	-2.322143000	3.415877000
H	1.426107000	-2.165807000	2.609486000
C	0.677197000	-3.791124000	3.830682000

H	1.014237000	-1.693084000	4.257092000
H	0.380261000	-4.429907000	2.991836000
H	-0.035337000	-3.957151000	4.645342000
H	1.660498000	-4.124514000	4.172488000
C	-1.602444000	1.283838000	5.582768000
C	-1.111293000	1.174793000	7.017236000
C	-3.129843000	1.308465000	5.542225000
C	-0.957367000	2.442126000	4.755121000
C	0.497200000	2.706667000	5.142609000
C	-1.749271000	3.739542000	4.747446000
H	0.942841000	3.367784000	4.394975000
H	1.072561000	1.775704000	5.163634000
H	0.570014000	3.186438000	6.123026000
H	-2.739856000	3.596672000	4.312396000
H	-1.218563000	4.487375000	4.152333000
H	-1.860534000	4.126322000	5.766033000
H	-1.619295000	0.344518000	7.514986000
H	-1.333929000	2.094199000	7.569135000
H	-0.036288000	0.990565000	7.055804000
H	-3.506229000	0.369304000	5.955731000
H	-3.492787000	1.401942000	4.514146000
H	-3.532118000	2.136690000	6.132915000
H	-1.020187000	-2.469968000	2.115830000
H	-1.393433000	-1.957116000	3.758033000

TS8

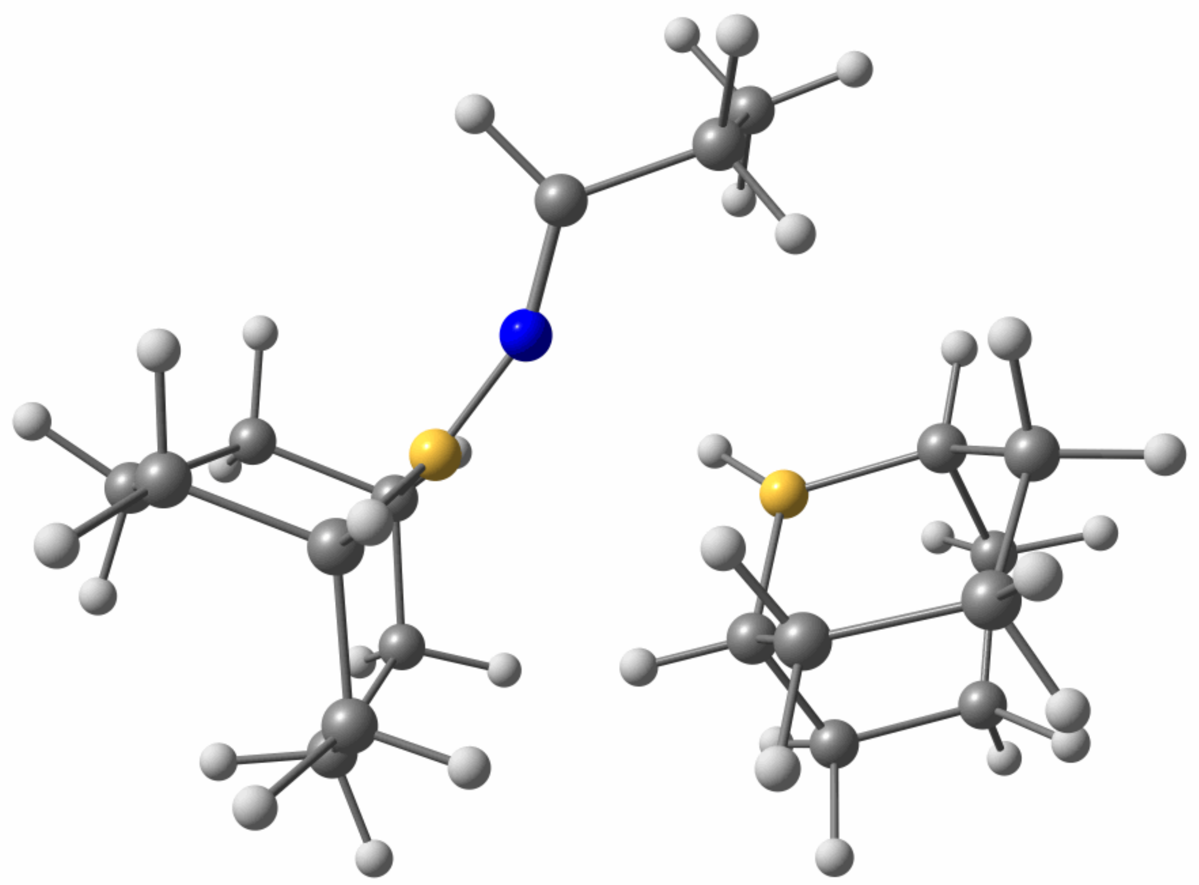

N	-0.951728000	1.912183000	-1.334930000
C	-1.364912000	2.829059000	-2.083550000
H	-1.233312000	2.749459000	-3.174846000
C	-2.002637000	4.099049000	-1.591404000
H	-2.316316000	3.950210000	-0.555194000
H	-2.896505000	4.307477000	-2.191360000
C	-1.024256000	5.275848000	-1.689874000
H	-0.150864000	5.096271000	-1.056246000
H	-1.501803000	6.203639000	-1.363986000
H	-0.677735000	5.417203000	-2.718595000
H	0.402972000	3.339106000	0.833412000
B	-0.609129000	3.149545000	1.445711000
H	-1.371772000	5.271750000	1.422393000
H	-0.420661000	0.937553000	1.733710000
H	-3.469338000	4.204907000	1.157813000

H	-2.702640000	1.363357000	0.959240000
C	-1.490229000	4.334121000	1.979611000
C	-0.965019000	1.792282000	2.147202000
H	0.640973000	1.925119000	3.584444000
H	0.298877000	4.760100000	3.165059000
C	-2.996260000	4.033733000	2.133161000
C	-2.473494000	1.504881000	2.026252000
C	-0.455917000	1.981906000	3.608887000
C	-0.759880000	4.528626000	3.351632000
H	-3.453927000	4.766645000	2.812850000
H	-2.718773000	0.555084000	2.522237000
C	-3.371200000	2.610699000	2.597493000
H	-0.792309000	1.137101000	4.225745000
H	-1.179816000	5.410916000	3.851685000
C	-0.851230000	3.311153000	4.285206000
H	-4.409830000	2.412383000	2.308044000
H	-0.194122000	3.476594000	5.147017000
H	-3.359022000	2.550401000	3.688605000
H	-1.861983000	3.248517000	4.693928000
C	-0.640116000	-1.347325000	-2.278133000
C	-1.066565000	-0.674318000	-0.944578000
C	1.242155000	0.579937000	-0.876147000
C	1.619708000	-0.091294000	-2.225377000
C	-0.661400000	-1.465868000	0.315285000
C	1.648557000	-0.241258000	0.363328000
H	-2.159432000	-0.578189000	-0.955910000
H	1.750600000	1.550617000	-0.832462000
B	-0.329495000	0.724892000	-0.996945000
C	0.853405000	-1.543629000	0.598727000
C	0.880025000	-1.409201000	-2.520890000
H	1.399832000	0.622907000	-3.032373000
H	2.703480000	-0.268667000	-2.260372000

H	-1.101408000	-0.782718000	-3.101210000
H	-1.057232000	-2.362510000	-2.331291000
H	1.317024000	-2.216524000	-1.929109000
H	1.056920000	-1.684722000	-3.567499000
H	0.995296000	-1.853190000	1.641251000
H	1.289853000	-2.347201000	0.000813000
H	1.541896000	0.408010000	1.241606000
H	2.717769000	-0.490632000	0.307289000
H	-1.163884000	-1.004771000	1.175197000
H	-1.057394000	-2.489338000	0.252232000

TS9

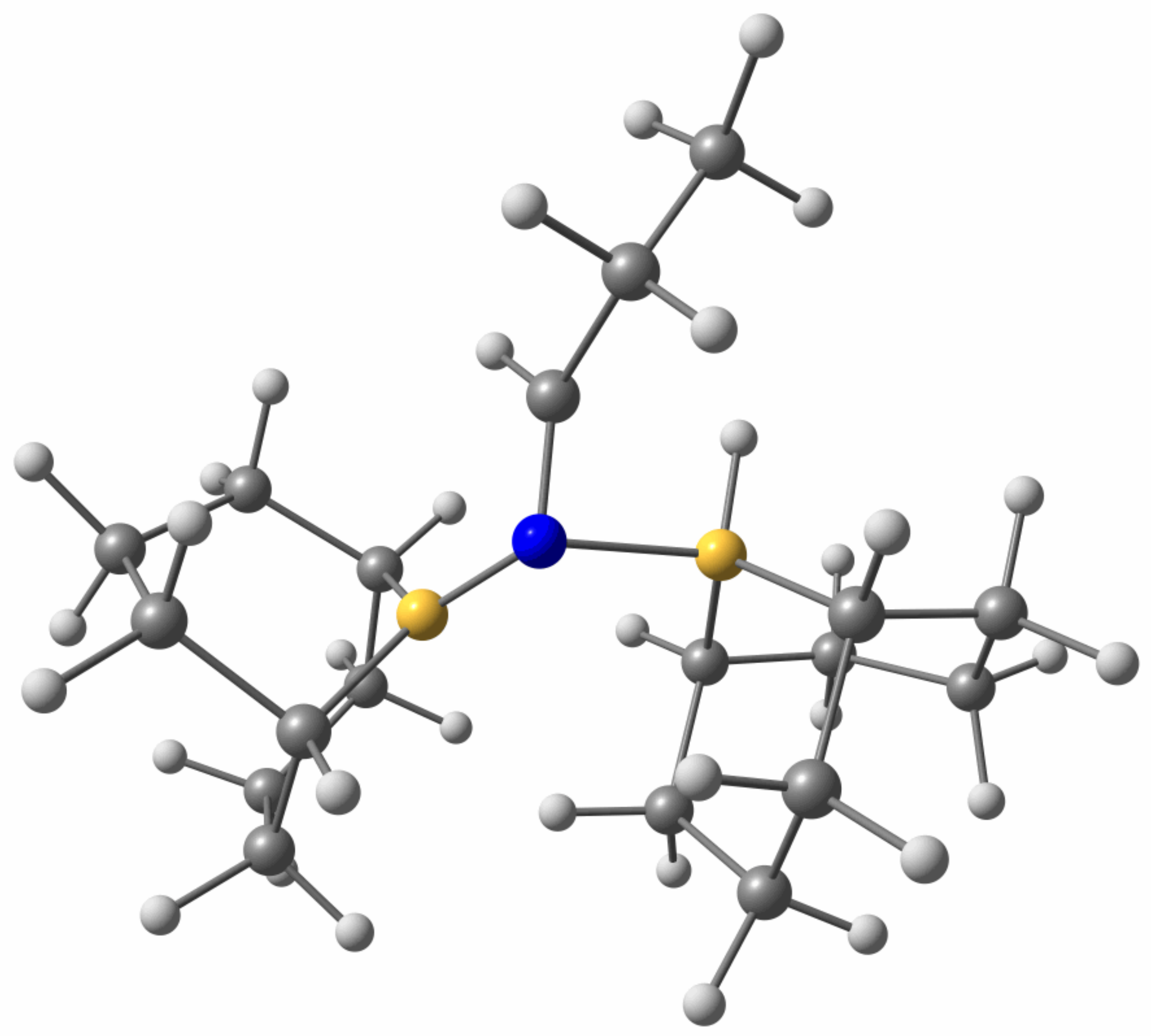

N	-0.930827000	2.506409000	-0.090192000
C	-0.540134000	3.309443000	-1.041872000
H	0.398728000	3.117466000	-1.591060000
C	-1.413249000	4.358316000	-1.658390000
H	-2.268590000	4.548515000	-1.008048000
H	-1.804962000	3.865351000	-2.562353000
C	-0.682076000	5.638458000	-2.053782000
H	-0.385877000	6.199421000	-1.164373000
H	-1.326746000	6.272940000	-2.665697000
H	0.220889000	5.417764000	-2.632696000
H	0.155933000	4.623673000	0.364015000
B	-0.493417000	3.824849000	1.046065000

H	-2.530156000	4.838425000	1.024781000
H	1.385115000	2.755013000	1.766935000
H	-2.909776000	2.572943000	1.703265000
H	-0.373342000	1.216285000	2.325978000
C	-1.813197000	4.408254000	1.738015000
C	0.466313000	3.195882000	2.177706000
H	1.534730000	5.045765000	2.422012000
H	-0.992160000	6.377543000	1.989816000
C	-2.542196000	3.266120000	2.471371000
C	-0.273402000	2.102774000	2.970488000
C	0.912892000	4.392727000	3.048882000
C	-1.352863000	5.572593000	2.646360000
H	-3.427873000	3.648704000	2.998373000
H	0.336367000	1.767479000	3.823079000
C	-1.682321000	2.473346000	3.474311000
H	1.557918000	4.039569000	3.866214000
H	-2.207798000	5.986706000	3.199782000
C	-0.232393000	5.229122000	3.645380000
H	-2.211670000	1.548721000	3.738067000
H	0.184603000	6.163147000	4.041805000
H	-1.598504000	3.032752000	4.409212000
H	-0.652774000	4.710811000	4.510763000
C	-1.136781000	-0.530751000	-1.353545000
C	-1.071551000	-0.109678000	0.146168000
C	1.283416000	0.922398000	-0.300450000
C	1.286573000	0.282652000	-1.723842000
C	-0.432613000	-1.177996000	1.060640000
C	1.866644000	-0.007553000	0.787352000
H	-2.097782000	0.082927000	0.481853000
H	1.900741000	1.829960000	-0.344013000
B	-0.228631000	1.207674000	0.032114000
C	1.101055000	-1.328119000	0.967722000

C	0.238458000	-0.822421000	-1.973346000
H	1.111371000	1.084742000	-2.454608000
H	2.290055000	-0.107947000	-1.941648000
H	-1.630543000	0.270954000	-1.923959000
H	-1.780220000	-1.413879000	-1.456463000
H	0.601809000	-1.782017000	-1.600518000
H	0.121416000	-0.953243000	-3.055497000
H	1.457770000	-1.816560000	1.881775000
H	1.367328000	-2.008393000	0.155178000
H	1.875197000	0.537320000	1.740920000
H	2.915995000	-0.232501000	0.554613000
H	-0.689552000	-0.917386000	2.094934000
H	-0.900451000	-2.154559000	0.873780000

IM8

C	-1.908355000	-1.183092000	0.577728000
C	-0.366907000	-1.265730000	0.432293000
C	-0.341297000	1.309495000	0.080446000
C	-1.885623000	1.339454000	0.042592000
C	0.126350000	-1.491050000	-1.016857000
C	0.320383000	1.056637000	-1.303413000
H	-0.042895000	-2.130453000	1.025139000
H	0.013131000	2.291870000	0.407869000
B	0.183166000	0.118090000	0.980405000
C	-0.029195000	-0.285064000	-1.960744000
C	-2.571177000	-0.022748000	-0.178902000
N	1.116415000	0.230582000	2.076868000
H	-2.231553000	1.740178000	1.005860000
H	-2.230596000	2.049534000	-0.721954000
H	-2.142271000	-1.085335000	1.648903000
H	-2.360842000	-2.130418000	0.254370000
H	-2.609377000	-0.260997000	-1.244094000
H	-3.617497000	0.054278000	0.139691000
H	0.620364000	-0.432884000	-2.831743000
H	-1.045029000	-0.241611000	-2.359304000
H	1.411639000	1.112951000	-1.172917000
H	0.054548000	1.875135000	-1.985859000
H	1.194761000	-1.746533000	-0.965050000
H	-0.373448000	-2.367250000	-1.453097000
C	1.930249000	-0.964808000	2.374193000
C	1.395857000	-1.761934000	3.559309000
H	1.376331000	-1.109743000	4.441723000
C	2.234415000	-3.006124000	3.839834000
H	0.353959000	-2.038759000	3.353905000
H	3.272293000	-2.736382000	4.062408000
H	2.242592000	-3.676046000	2.973402000

H	1.844467000	-3.567456000	4.692667000
H	2.962316000	-0.656190000	2.569032000
H	1.968253000	-1.607411000	1.488765000
H	3.004791000	4.780535000	2.835070000
H	1.238633000	3.867314000	1.584531000
C	2.400958000	4.049946000	3.386340000
H	2.253498000	4.484465000	4.377469000
H	3.617716000	2.480299000	2.537235000
H	0.427528000	4.774170000	2.850755000
C	1.047233000	3.885244000	2.666815000
H	4.065483000	2.919318000	4.178408000
C	3.202524000	2.745934000	3.521145000
H	1.003203000	3.751733000	5.640957000
C	0.251391000	2.603444000	3.015276000
C	2.383373000	1.534256000	4.043182000
H	-0.586616000	2.532739000	2.316491000
B	1.232335000	1.359363000	2.961667000
H	3.075847000	0.686270000	4.103087000
H	-1.102699000	3.429111000	4.507291000
H	2.609926000	2.035190000	6.151830000
C	0.634258000	2.725322000	5.582136000
C	-0.373426000	2.610950000	4.433237000
C	1.806212000	1.735372000	5.464618000
H	0.115992000	2.546196000	6.531749000
H	-0.947456000	1.679765000	4.556345000
H	1.456349000	0.756117000	5.820004000

TS10

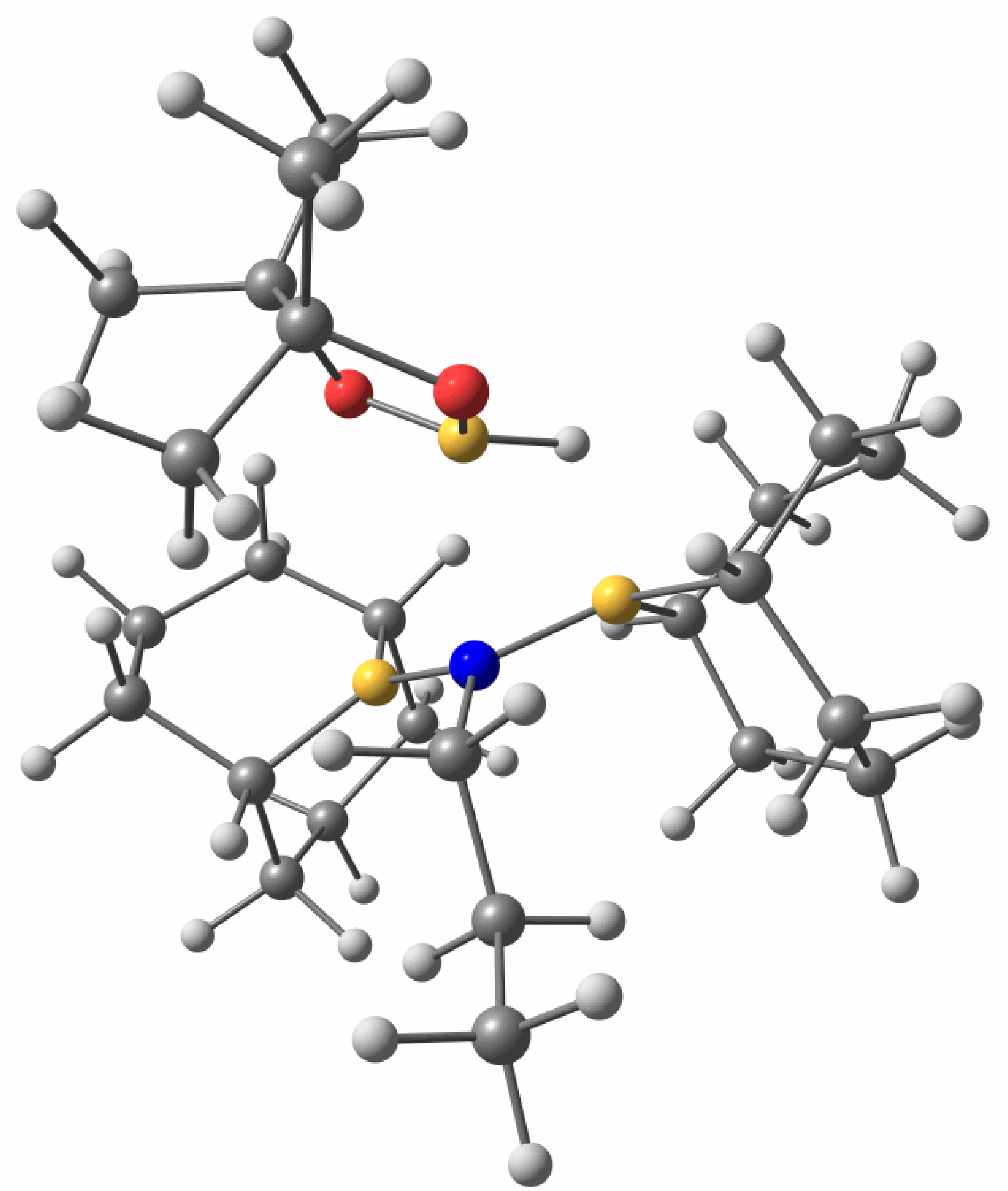

C	-1.088483000	-2.480952000	2.160353000
C	-0.444263000	-1.555985000	1.097990000
C	-1.603671000	0.523706000	2.137288000
C	-2.092714000	-0.372355000	3.304694000
C	-1.336035000	-1.402980000	-0.153726000
C	-2.560315000	0.584387000	0.928807000

H	0.475401000	-2.041524000	0.754509000
H	-1.542748000	1.536146000	2.540332000
B	-0.190546000	-0.108181000	1.751793000
C	-2.679379000	-0.704529000	0.097993000
C	-2.251124000	-1.866304000	2.970575000
N	1.092167000	0.233608000	2.502575000
H	-1.377355000	-0.259954000	4.133331000
H	-3.050626000	0.010143000	3.682873000
H	-0.303589000	-2.805647000	2.853344000
H	-1.443586000	-3.398605000	1.670625000
H	-3.191426000	-2.009142000	2.432743000
H	-2.365112000	-2.426845000	3.906727000
H	-3.145397000	-0.463741000	-0.865039000
H	-3.364482000	-1.402542000	0.586089000
H	-2.199693000	1.388065000	0.269814000
H	-3.560898000	0.894542000	1.260409000
H	-0.772426000	-0.844024000	-0.913932000
H	-1.524463000	-2.394358000	-0.587669000
C	1.921443000	-0.923186000	2.906023000
C	1.553840000	-1.479417000	4.282411000
H	1.798535000	-0.738244000	5.050605000
C	2.288396000	-2.787643000	4.567043000
H	0.471320000	-1.633246000	4.340954000
H	3.373304000	-2.650225000	4.502383000
H	2.007776000	-3.559103000	3.841497000
H	2.058733000	-3.166199000	5.566321000
H	2.976785000	-0.637119000	2.916723000
H	1.827892000	-1.708202000	2.151012000
H	3.589507000	4.761801000	2.777566000
H	1.819888000	3.954288000	1.491349000
C	2.878221000	4.154119000	3.351109000
H	2.723685000	4.697554000	4.286380000

H	3.992687000	2.408130000	2.749095000
H	1.010417000	4.995586000	2.654311000
C	1.565500000	4.052076000	2.550362000
H	4.342060000	2.944061000	4.383719000
C	3.524664000	2.799597000	3.663701000
H	1.247058000	4.219782000	5.420865000
C	0.616712000	2.872736000	2.888504000
C	2.544939000	1.729769000	4.195123000
H	-0.115358000	2.831096000	2.074702000
B	1.399093000	1.511669000	3.113772000
H	3.140436000	0.834130000	4.408264000
H	-0.787875000	3.976062000	4.146457000
H	2.616361000	2.420089000	6.263933000
C	0.781105000	3.232448000	5.438331000
C	-0.147002000	3.087220000	4.224325000
C	1.852740000	2.129148000	5.528693000
H	0.178591000	3.207366000	6.354104000
H	-0.821607000	2.236297000	4.400149000
H	1.368500000	1.229511000	5.933674000
C	2.931807000	1.805457000	-0.720427000
C	3.281119000	0.289755000	-0.569771000
H	0.058445000	0.708617000	0.407637000
B	1.282986000	0.738985000	0.404487000
O	2.006620000	-0.276616000	-0.184201000
O	1.891718000	1.976226000	0.267491000
C	4.278978000	0.034024000	0.556284000
H	4.303452000	-1.037286000	0.773438000
H	5.284385000	0.358699000	0.271749000
H	3.988185000	0.566283000	1.467127000
C	3.740356000	-0.390013000	-1.849548000
H	4.642655000	0.094401000	-2.237780000
H	3.974908000	-1.437559000	-1.642463000

H	2.963201000	-0.358599000	-2.615009000
C	2.316683000	2.133850000	-2.081048000
H	3.063824000	2.089538000	-2.878911000
H	1.504875000	1.440360000	-2.321760000
H	1.904210000	3.145227000	-2.043217000
C	4.069903000	2.765069000	-0.412528000
H	4.909021000	2.596002000	-1.095827000
H	3.723221000	3.794055000	-0.543709000
H	4.422645000	2.653788000	0.614244000

IM9

C	-1.146580000	-2.470917000	2.121008000
C	-0.522771000	-1.549326000	1.050416000
C	-1.656015000	0.529448000	2.132454000
C	-2.117838000	-0.360206000	3.305563000
C	-1.435462000	-1.381099000	-0.184795000
C	-2.645157000	0.588486000	0.946047000
H	0.388531000	-2.034948000	0.684371000
H	-1.596088000	1.547033000	2.524957000
B	-0.257829000	-0.059099000	1.612171000
C	-2.777227000	-0.698035000	0.112496000
C	-2.276309000	-1.855931000	2.978008000
N	1.133484000	0.220492000	2.431617000
H	-1.389710000	-0.240979000	4.121964000
H	-3.071809000	0.013395000	3.703905000
H	-0.344706000	-2.816223000	2.784763000
H	-1.531221000	-3.380917000	1.638303000
H	-3.236682000	-2.001651000	2.476987000
H	-2.354381000	-2.417807000	3.917521000
H	-3.277629000	-0.456994000	-0.833260000
H	-3.437663000	-1.407370000	0.618682000
H	-2.302513000	1.394034000	0.280096000
H	-3.639886000	0.890774000	1.302951000
H	-0.888322000	-0.794582000	-0.937898000
H	-1.626779000	-2.362044000	-0.641279000
C	1.859866000	-0.978617000	2.938531000
C	1.383651000	-1.435263000	4.312215000
H	1.581680000	-0.659682000	5.058445000
C	2.072439000	-2.735582000	4.721882000
H	0.299029000	-1.576695000	4.293786000
H	3.160692000	-2.613993000	4.744573000
H	1.841719000	-3.539436000	4.014630000

H	1.750566000	-3.058825000	5.714825000
H	2.932622000	-0.754764000	2.967071000
H	1.732520000	-1.782272000	2.211267000
H	3.559141000	4.704674000	2.877999000
H	1.732073000	4.010800000	1.620103000
C	2.855220000	4.088303000	3.450042000
H	2.743558000	4.591905000	4.413989000
H	3.865860000	2.323810000	2.730341000
H	0.960498000	4.972868000	2.877159000
C	1.513914000	4.041580000	2.690905000
H	4.346626000	2.809579000	4.353707000
C	3.481434000	2.710273000	3.684802000
H	1.167637000	4.186382000	5.477962000
C	0.580005000	2.837062000	2.978794000
C	2.496078000	1.668517000	4.276931000
H	-0.142434000	2.804787000	2.159087000
B	1.374848000	1.492043000	3.176711000
H	3.077006000	0.763260000	4.484407000
H	-0.890261000	3.826227000	4.253118000
H	2.640585000	2.468056000	6.298799000
C	0.740596000	3.182078000	5.522475000
C	-0.191258000	2.982445000	4.321546000
C	1.852585000	2.118242000	5.617580000
H	0.150322000	3.151564000	6.445581000
H	-0.809980000	2.088545000	4.493841000
H	1.417917000	1.227878000	6.091415000
C	2.747311000	1.719647000	-0.565635000
C	3.202742000	0.225865000	-0.528143000
H	0.108344000	0.636200000	0.549988000
B	1.456580000	0.564938000	0.920136000
O	2.100007000	-0.412336000	0.134749000
O	2.027214000	1.835460000	0.671380000

C	4.449342000	0.018901000	0.333019000
H	4.579591000	-1.052786000	0.507509000
H	5.348429000	0.406124000	-0.156180000
H	4.339750000	0.515197000	1.303355000
C	3.390624000	-0.414544000	-1.894825000
H	4.148493000	0.125046000	-2.473266000
H	3.724150000	-1.448632000	-1.772253000
H	2.454305000	-0.423107000	-2.455743000
C	1.773312000	2.005324000	-1.709819000
H	2.281319000	2.006813000	-2.678804000
H	0.970859000	1.261078000	-1.738879000
H	1.324309000	2.989223000	-1.548516000
C	3.877847000	2.736582000	-0.563108000
H	4.521978000	2.600716000	-1.438467000
H	3.461375000	3.747552000	-0.597715000
H	4.485611000	2.648711000	0.340118000

TS11

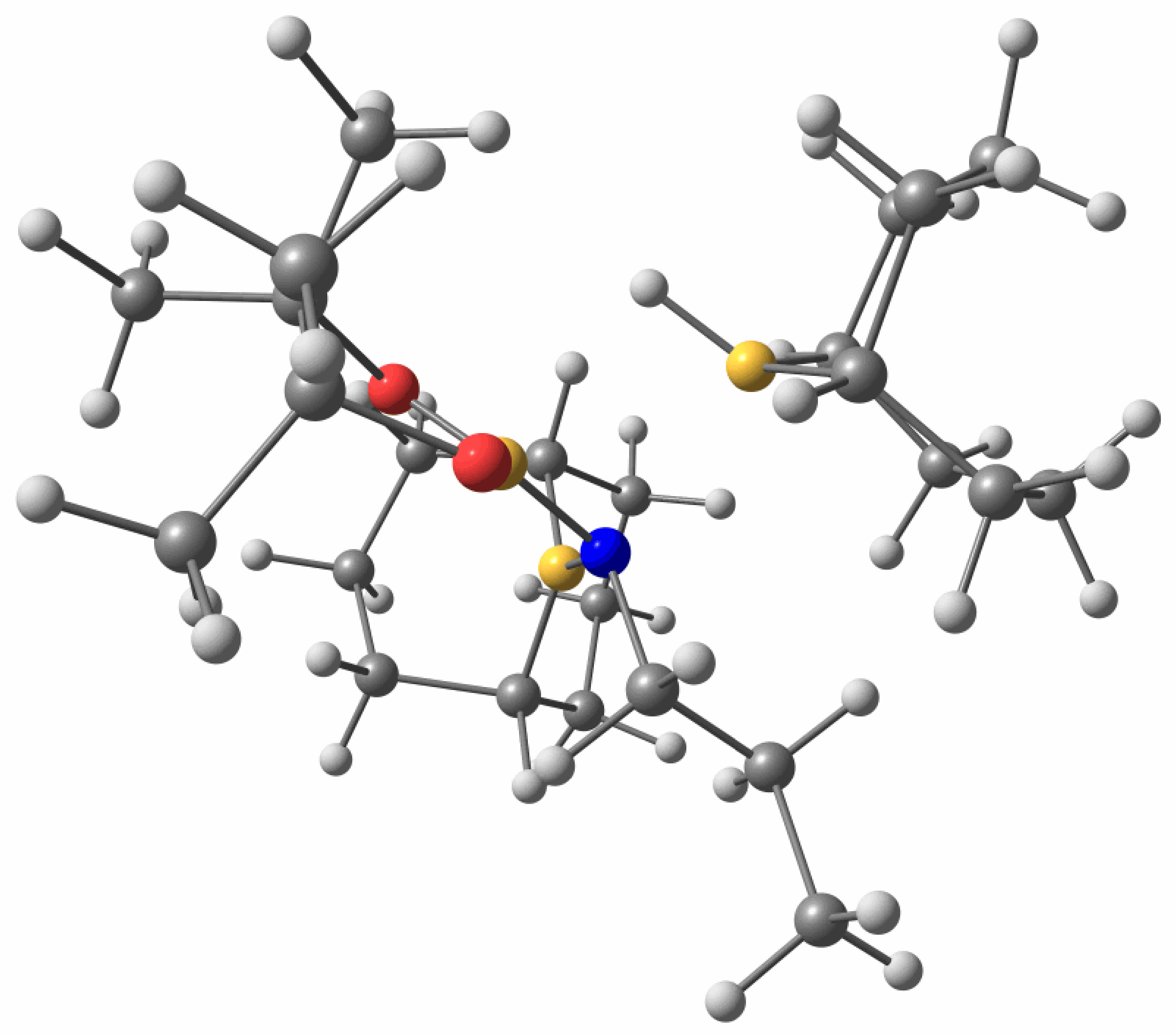

C	-1.165821000	-2.449291000	1.920317000
C	-0.697658000	-1.469145000	0.824353000
C	-1.880508000	0.498007000	2.077572000
C	-2.214027000	-0.432127000	3.251631000
C	-1.738564000	-1.352122000	-0.325617000
C	-2.968639000	0.510356000	0.961602000
H	0.224572000	-1.860970000	0.377259000
H	-1.833885000	1.525095000	2.454595000
B	-0.587385000	0.049010000	1.279864000
C	-3.090595000	-0.779738000	0.129002000
C	-2.244851000	-1.936743000	2.907912000
N	1.315617000	0.276016000	2.424774000

H	-1.475020000	-0.245114000	4.042654000
H	-3.186274000	-0.160268000	3.687611000
H	-0.285276000	-2.767737000	2.488293000
H	-1.545977000	-3.367432000	1.448786000
H	-3.235444000	-2.176125000	2.512618000
H	-2.166305000	-2.512879000	3.839418000
H	-3.709320000	-0.573063000	-0.752679000
H	-3.632002000	-1.542757000	0.694756000
H	-2.734216000	1.338669000	0.279419000
H	-3.943957000	0.745842000	1.410225000
H	-1.324120000	-0.709666000	-1.115206000
H	-1.896868000	-2.340582000	-0.778605000
C	1.821298000	-1.002870000	3.001253000
C	1.123303000	-1.445412000	4.281316000
H	1.219464000	-0.681416000	5.058531000
C	1.706930000	-2.766079000	4.780535000
H	0.055163000	-1.562907000	4.093434000
H	2.776889000	-2.670011000	4.993981000
H	1.586573000	-3.555379000	4.030312000
H	1.210216000	-3.096025000	5.696302000
H	2.901015000	-0.912341000	3.191763000
H	1.705898000	-1.778227000	2.238202000
H	3.640723000	4.574321000	3.263431000
H	1.988879000	3.927050000	1.753831000
C	2.832373000	3.999772000	3.731247000
H	2.601172000	4.525640000	4.661047000
H	3.843368000	2.170251000	3.186917000
H	1.082988000	4.949730000	2.864534000
C	1.616618000	3.992373000	2.780599000
H	4.137891000	2.680739000	4.843370000
C	3.356159000	2.600921000	4.076371000
H	0.888770000	4.209704000	5.508241000

C	0.612538000	2.824873000	2.962295000
C	2.257625000	1.613304000	4.568283000
H	-0.000710000	2.770430000	2.056290000
B	1.353642000	1.472087000	3.279081000
H	2.775089000	0.692092000	4.859357000
H	-0.991883000	3.874423000	4.004432000
H	2.155356000	2.460917000	6.575074000
C	0.420849000	3.222556000	5.507978000
C	-0.327027000	3.019368000	4.185816000
C	1.462013000	2.121560000	5.792836000
H	-0.303965000	3.239097000	6.330227000
H	-0.982496000	2.141620000	4.287079000
H	0.927282000	1.259692000	6.215768000
C	2.720215000	1.613984000	-0.711757000
C	3.255739000	0.143088000	-0.667406000
H	-0.171672000	0.834663000	0.467357000
B	1.897583000	0.533676000	1.105144000
O	2.384746000	-0.473328000	0.306168000
O	2.205610000	1.786377000	0.627105000
C	4.675919000	0.045466000	-0.112121000
H	4.905739000	-1.005934000	0.078652000
H	5.411090000	0.442672000	-0.817992000
H	4.765544000	0.592959000	0.831930000
C	3.142332000	-0.615589000	-1.979383000
H	3.716104000	-0.111159000	-2.764040000
H	3.545055000	-1.624064000	-1.853648000
H	2.102088000	-0.699880000	-2.298656000
C	1.556649000	1.794632000	-1.684926000
H	1.905031000	1.770305000	-2.721842000
H	0.798231000	1.019653000	-1.547458000
H	1.089009000	2.764212000	-1.493501000
C	3.783442000	2.673178000	-0.956017000

H	4.279005000	2.505828000	-1.918122000
H	3.313734000	3.660347000	-0.979988000
H	4.535178000	2.670873000	-0.164165000

TS12

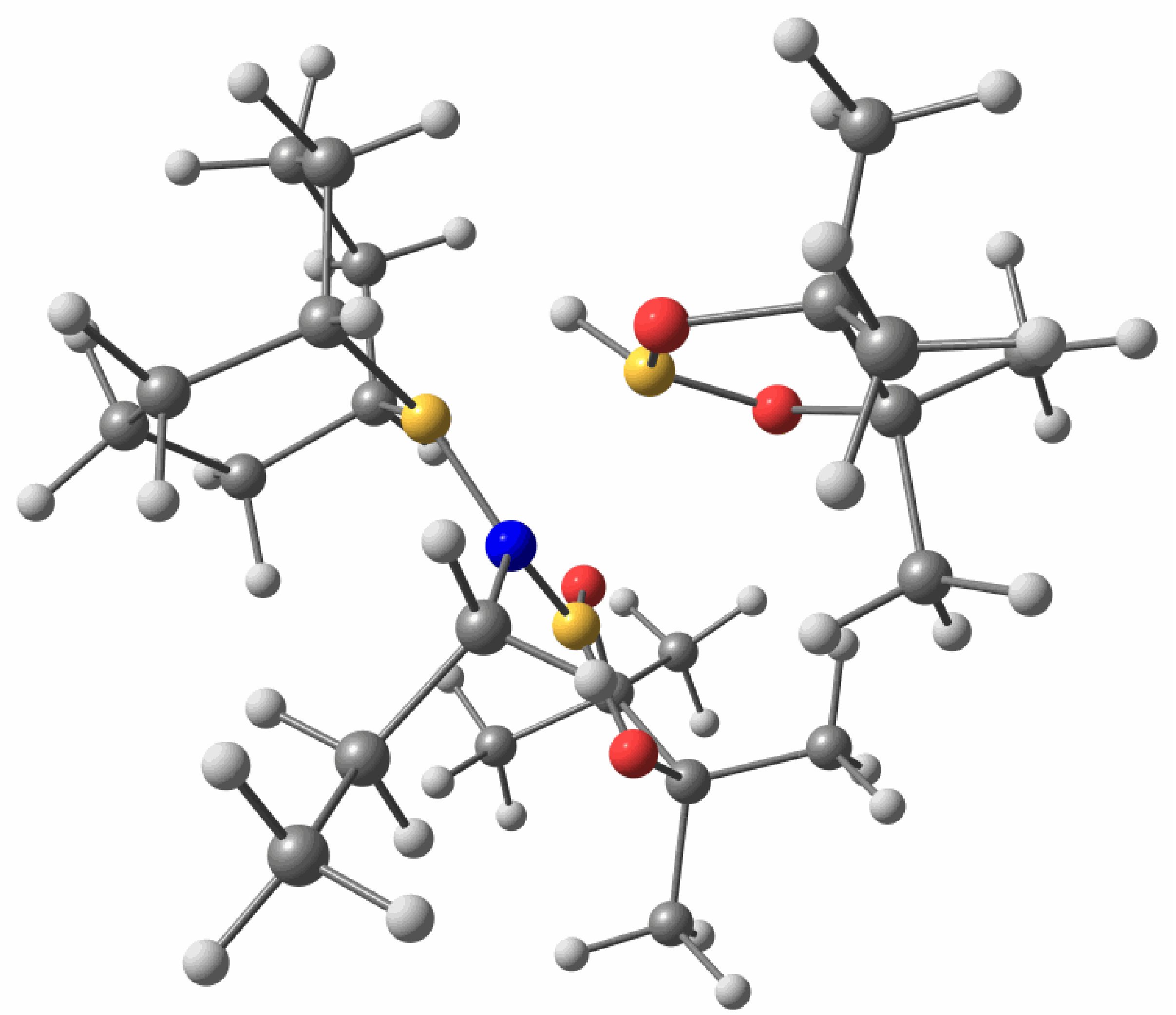

N	2.861671000	0.298821000	-1.870502000
C	2.740208000	-1.150722000	-1.611100000
B	2.911432000	1.124020000	-0.682705000
O	3.233199000	2.459693000	-0.661900000
O	2.654534000	0.623839000	0.577035000
C	3.994725000	-1.745316000	-0.967662000
H	4.877031000	-1.441330000	-1.542927000
C	3.913379000	-3.268613000	-0.899345000
H	4.110713000	-1.327515000	0.038186000
H	3.848511000	-3.702479000	-1.903107000
H	3.027791000	-3.588789000	-0.340095000
H	4.791261000	-3.693411000	-0.405681000
C	2.678414000	1.742881000	1.493026000

C	3.285421000	1.283567000	2.808977000
C	1.239065000	2.204854000	1.698346000
C	3.532090000	2.791414000	0.708407000
C	5.036498000	2.587936000	0.892831000
C	3.155952000	4.242350000	0.958778000
H	5.563961000	3.199650000	0.155966000
H	5.313897000	1.541341000	0.726827000
H	5.363448000	2.882855000	1.894298000
H	2.126014000	4.439896000	0.657689000
H	3.813562000	4.893492000	0.376898000
H	3.274204000	4.492190000	2.018550000
H	2.618608000	0.560305000	3.285923000
H	3.412974000	2.133175000	3.488222000
H	4.254818000	0.806034000	2.654900000
H	0.652449000	1.366194000	2.082974000
H	0.801956000	2.535047000	0.750355000
H	1.187682000	3.024681000	2.420937000
H	2.518480000	-1.654843000	-2.554290000
H	1.888560000	-1.332854000	-0.947465000
H	-1.287350000	-1.985283000	-3.820584000
H	-1.260987000	0.003444000	-5.216727000
C	-1.350246000	-1.415527000	-2.889598000
H	-0.771474000	-1.941232000	-2.127224000
C	-1.481971000	0.618544000	-4.340577000
H	-2.567482000	0.678597000	-4.217917000
H	-2.399734000	-1.379327000	-2.578160000
O	0.594589000	-0.107834000	-3.424744000
H	-1.091907000	1.624557000	-4.523916000
C	-0.807614000	-0.013530000	-3.121733000
B	1.199913000	1.030580000	-2.902514000
H	0.166642000	-0.485569000	-0.525616000
C	-0.831758000	0.918727000	-1.859893000

O	0.359325000	1.717103000	-2.032157000
H	-2.962285000	1.292933000	-1.748464000
H	-2.058100000	2.524996000	-2.661070000
C	-0.722623000	0.147871000	-0.547315000
C	-2.026669000	1.860749000	-1.796164000
H	-0.647768000	0.859006000	0.276222000
H	-1.605532000	-0.476938000	-0.382750000
H	-1.953200000	2.476741000	-0.895511000
C	3.200625000	0.588931000	-5.755929000
C	3.462784000	-0.206022000	-4.460455000
C	4.420677000	1.955467000	-3.363364000
C	4.257228000	2.689589000	-4.709912000
C	4.869008000	-0.861648000	-4.425487000
C	5.781494000	1.230246000	-3.184433000
H	2.722419000	-1.012341000	-4.436136000
H	4.350830000	2.703780000	-2.572401000
B	3.380512000	0.768087000	-3.200481000
C	6.055288000	0.099366000	-4.192535000
C	4.117802000	1.802723000	-5.959333000
H	1.966150000	1.691357000	-3.586352000
H	3.355122000	3.312111000	-4.628969000
H	5.092763000	3.388674000	-4.855578000
H	2.153642000	0.922515000	-5.742011000
H	3.295300000	-0.081361000	-6.621326000
H	5.101737000	1.462140000	-6.292479000
H	3.726687000	2.412236000	-6.783011000
H	6.917512000	-0.483936000	-3.846330000
H	6.366235000	0.540076000	-5.142568000
H	5.812113000	0.818660000	-2.163358000
H	6.599604000	1.961566000	-3.237503000
H	4.868470000	-1.619018000	-3.631073000
H	5.034671000	-1.414379000	-5.360941000

IM10

N	2.989570000	0.207192000	-1.842920000
C	3.189637000	-1.244155000	-1.543091000
B	3.143152000	1.040205000	-0.628154000
O	3.588922000	2.326911000	-0.596836000
O	2.859168000	0.529502000	0.611825000
C	4.553069000	-1.545082000	-0.924565000
H	5.337276000	-1.034544000	-1.495036000
C	4.818338000	-3.049289000	-0.904873000
H	4.585909000	-1.149628000	0.096187000
H	4.849186000	-3.454276000	-1.921990000
H	4.030763000	-3.578785000	-0.358608000
H	5.772034000	-3.277334000	-0.422425000
C	2.927380000	1.637335000	1.543496000
C	3.476626000	1.131189000	2.866731000
C	1.506781000	2.167449000	1.712767000
C	3.859375000	2.641474000	0.788840000
C	5.344822000	2.363364000	1.013085000
C	3.546321000	4.108622000	1.028912000
H	5.922131000	2.953294000	0.296464000
H	5.576387000	1.305902000	0.846416000
H	5.655097000	2.635702000	2.026037000
H	2.535531000	4.355043000	0.700365000
H	4.250628000	4.727199000	0.466807000
H	3.646612000	4.351469000	2.092050000
H	2.761022000	0.439084000	3.318097000
H	3.631102000	1.965566000	3.558966000
H	4.423746000	0.605611000	2.730950000
H	0.873894000	1.356278000	2.082538000
H	1.106722000	2.509316000	0.752632000
H	1.474715000	2.990556000	2.432507000
H	3.060302000	-1.792527000	-2.477334000

H	2.392996000	-1.567973000	-0.864661000
H	-1.350498000	-1.897444000	-3.788120000
H	-0.424482000	-0.092055000	-5.170879000
C	-1.442055000	-1.265975000	-2.900200000
H	-1.193455000	-1.868941000	-2.024861000
C	-0.700981000	0.611517000	-4.380837000
H	-1.741776000	0.914898000	-4.529122000
H	-2.482525000	-0.932789000	-2.820596000
O	0.848659000	-0.547597000	-2.954953000
H	-0.064910000	1.497898000	-4.472467000
C	-0.504799000	-0.075230000	-3.027267000
B	1.603950000	0.540774000	-2.484936000
H	-0.047521000	-0.577087000	-0.356418000
C	-0.558271000	0.933426000	-1.832813000
O	0.772283000	1.485905000	-1.832348000
H	-2.575748000	1.670890000	-2.107464000
H	-1.322384000	2.672699000	-2.879330000
C	-0.777556000	0.227109000	-0.495375000
C	-1.559210000	2.065976000	-2.003619000
H	-0.646801000	0.950894000	0.311851000
H	-1.785984000	-0.191631000	-0.423434000
H	-1.532054000	2.713673000	-1.122489000
C	3.419182000	0.409427000	-5.816690000
C	3.689570000	-0.285648000	-4.465791000
C	4.368617000	2.021713000	-3.447844000
C	4.216268000	2.652257000	-4.849871000
C	5.146107000	-0.785173000	-4.329536000
C	5.772180000	1.461111000	-3.142944000
H	3.027645000	-1.160856000	-4.433315000
H	4.169510000	2.814432000	-2.723393000
B	3.362772000	0.786282000	-3.314866000
C	6.218223000	0.294957000	-4.046893000

C	4.247887000	1.681197000	-6.042369000
H	2.153942000	1.233900000	-3.572283000
H	3.248575000	3.174435000	-4.864134000
H	4.981471000	3.428061000	-4.996677000
H	2.349900000	0.665118000	-5.862464000
H	3.603548000	-0.291455000	-6.642842000
H	5.278838000	1.402795000	-6.278008000
H	3.874595000	2.201079000	-6.933313000
H	7.092865000	-0.190403000	-3.594734000
H	6.577981000	0.707495000	-4.992941000
H	5.777063000	1.133591000	-2.091616000
H	6.525725000	2.259157000	-3.207568000
H	5.175642000	-1.527772000	-3.522719000
H	5.434983000	-1.333528000	-5.237999000

TS13

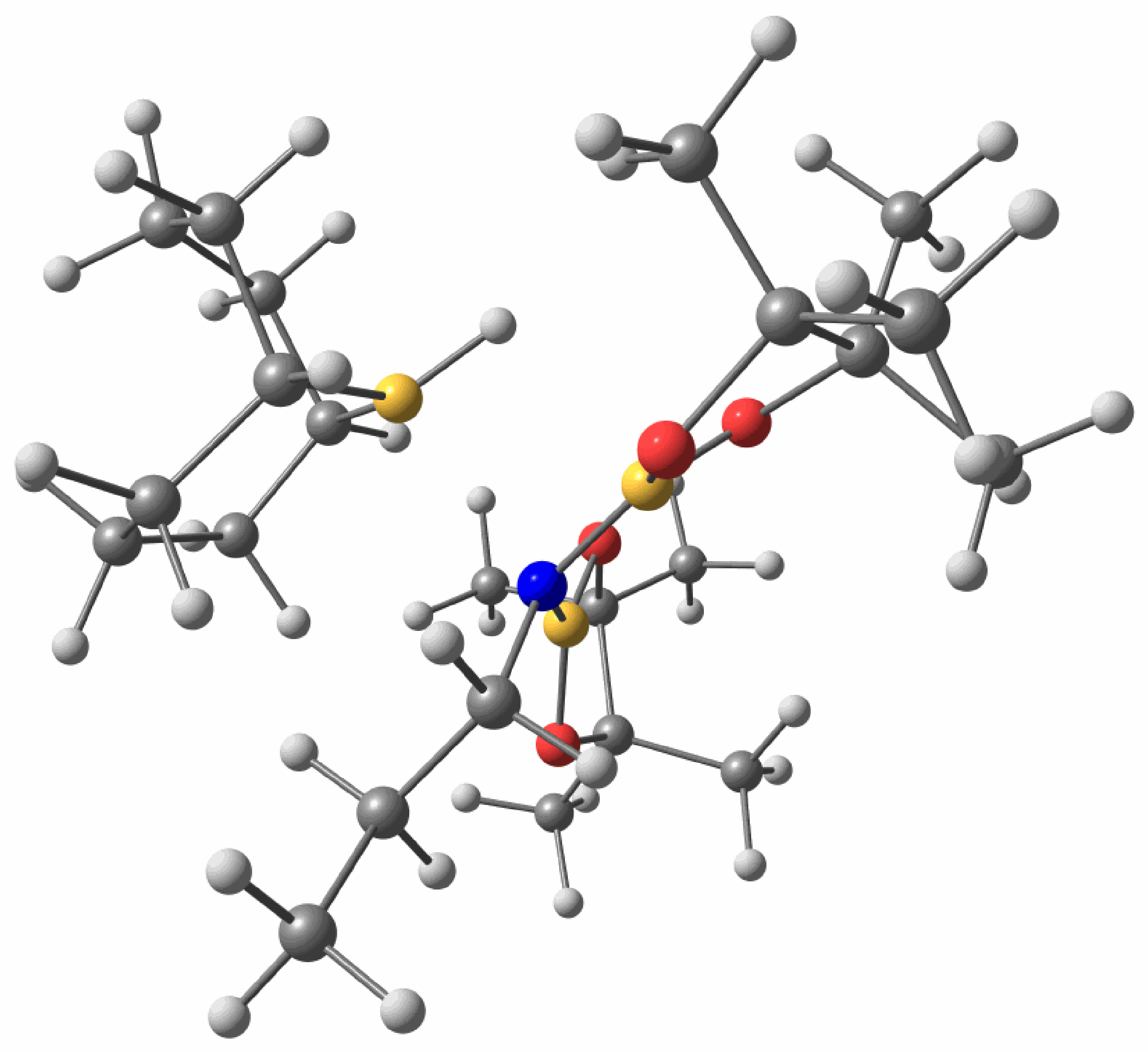

N	2.783499000	0.126905000	-1.649444000
C	3.121734000	-1.327296000	-1.592517000
B	3.211188000	1.008726000	-0.577141000
O	2.942071000	2.352455000	-0.560419000
O	3.882935000	0.587692000	0.550700000
C	4.522666000	-1.699646000	-1.116312000
H	5.272893000	-1.115811000	-1.656717000
C	4.765231000	-3.191483000	-1.346564000
H	4.638150000	-1.462069000	-0.057907000
H	4.711534000	-3.445872000	-2.410821000
H	4.016695000	-3.796497000	-0.823387000
H	5.750526000	-3.490023000	-0.979424000

C	3.801920000	1.668074000	1.507116000
C	5.065266000	1.674246000	2.351727000
C	2.576334000	1.392702000	2.377937000
C	3.618826000	2.916104000	0.581806000
C	4.948114000	3.493326000	0.098540000
C	2.749251000	4.017833000	1.167556000
H	4.748224000	4.236857000	-0.677324000
H	5.586975000	2.714979000	-0.330901000
H	5.486952000	3.980331000	0.916748000
H	1.737825000	3.660000000	1.366592000
H	2.684070000	4.844318000	0.455327000
H	3.184980000	4.397285000	2.098097000
H	5.100453000	0.768551000	2.963074000
H	5.073147000	2.540938000	3.021160000
H	5.959423000	1.701728000	1.726626000
H	2.687460000	0.407524000	2.838168000
H	1.661953000	1.391589000	1.775725000
H	2.473245000	2.139728000	3.170288000
H	2.963594000	-1.722330000	-2.600637000
H	2.391914000	-1.834334000	-0.945888000
H	-1.208591000	-1.903999000	-4.195934000
H	0.146106000	-0.254797000	-5.376005000
C	-1.485546000	-1.209608000	-3.398324000
H	-1.533131000	-1.766280000	-2.460798000
C	-0.249205000	0.520586000	-4.713809000
H	-1.188931000	0.893723000	-5.131867000
H	-2.479031000	-0.807196000	-3.623071000
O	0.802947000	-0.636937000	-2.903101000
H	0.474593000	1.339071000	-4.679525000
C	-0.462260000	-0.086972000	-3.327983000
B	1.420856000	0.324235000	-2.137161000
H	-0.935240000	-0.493256000	-0.636741000

C	-0.742434000	0.985555000	-2.219961000
O	0.585671000	1.358336000	-1.796080000
H	-2.446858000	1.962293000	-3.124256000
H	-0.887499000	2.746907000	-3.475337000
C	-1.448991000	0.404199000	-0.995726000
C	-1.467980000	2.227977000	-2.710841000
H	-1.432553000	1.150978000	-0.197579000
H	-2.489810000	0.148378000	-1.214791000
H	-1.621753000	2.914010000	-1.873725000
C	3.800895000	0.889935000	-5.948625000
C	3.748838000	0.043645000	-4.643768000
C	4.712819000	2.073722000	-3.307489000
C	4.869641000	2.847860000	-4.650348000
C	5.060615000	-0.731579000	-4.400566000
C	5.942851000	1.243092000	-2.917117000
H	2.930167000	-0.679261000	-4.761284000
H	4.535706000	2.827907000	-2.534720000
B	3.443820000	1.176073000	-3.581973000
C	6.269520000	0.080773000	-3.875322000
C	4.881747000	1.984211000	-5.927455000
H	2.377274000	1.727433000	-3.619973000
H	4.031297000	3.553576000	-4.722847000
H	5.783841000	3.457427000	-4.616152000
H	2.819349000	1.358606000	-6.106964000
H	3.971888000	0.227935000	-6.808720000
H	5.863257000	1.522092000	-6.061200000
H	4.744817000	2.639538000	-6.796227000
H	6.958854000	-0.610263000	-3.372193000
H	6.836553000	0.480026000	-4.720358000
H	5.770374000	0.847984000	-1.905225000
H	6.831822000	1.886302000	-2.836026000
H	4.847116000	-1.535734000	-3.689830000

H	5.365357000	-1.239455000	-5.327546000

IM11

N	-0.829961000	-0.293775000	2.574989000
C	-0.905668000	-1.747235000	2.796283000
B	-0.920073000	0.562101000	3.711761000
O	-0.912534000	1.938339000	3.680419000
O	-1.034919000	0.061172000	4.996858000
C	0.468294000	-2.368760000	3.033072000
H	1.104362000	-2.149674000	2.166214000
C	0.378776000	-3.876714000	3.253880000
H	0.925745000	-1.883314000	3.903640000
H	-0.064604000	-4.373394000	2.384389000
H	-0.243877000	-4.107990000	4.124560000
H	1.365741000	-4.315545000	3.422855000
C	-1.345997000	1.178910000	5.852340000
C	-0.704951000	0.955274000	7.212498000
C	-2.867505000	1.237781000	5.983757000
C	-0.763009000	2.383667000	5.041780000
C	0.731504000	2.591512000	5.286787000
C	-1.514974000	3.693114000	5.218147000
H	1.110840000	3.294876000	4.541370000
H	1.280169000	1.650498000	5.179724000
H	0.922512000	2.997897000	6.284534000
H	-2.550781000	3.602022000	4.886757000
H	-1.032699000	4.471806000	4.621454000
H	-1.502693000	4.005108000	6.268046000
H	-1.177613000	0.099991000	7.702924000
H	-0.841178000	1.835564000	7.849806000
H	0.362486000	0.748537000	7.118268000
H	-3.222862000	0.278614000	6.369268000
H	-3.337098000	1.413348000	5.011068000
H	-3.179681000	2.028879000	6.671930000
H	-1.372123000	-2.212609000	1.922080000

H	-1.547392000	-1.936855000	3.662234000
H	0.285970000	3.530097000	-0.755285000
H	-2.039485000	2.803507000	-0.522848000
C	0.636753000	2.538463000	-1.052945000
H	1.626243000	2.382667000	-0.619690000
C	-1.770221000	1.854635000	-0.993409000
H	-1.848176000	1.965179000	-2.079141000
H	0.717914000	2.514303000	-2.145022000
O	-0.329380000	1.459325000	0.872461000
H	-2.485904000	1.094558000	-0.665293000
C	-0.348891000	1.487615000	-0.567500000
B	-0.564184000	0.156112000	1.248014000
H	1.998633000	0.160871000	-0.022968000
C	0.030095000	0.014719000	-0.934755000
O	-0.509471000	-0.722435000	0.179619000
H	-0.275003000	0.107519000	-3.073362000
H	-1.682514000	-0.493252000	-2.165669000
C	1.540092000	-0.222505000	-0.939942000
C	-0.592618000	-0.502923000	-2.221259000
H	1.724211000	-1.298881000	-0.992235000
H	2.019079000	0.257041000	-1.798697000
H	-0.267487000	-1.532070000	-2.396073000

S8 NMR Spectra

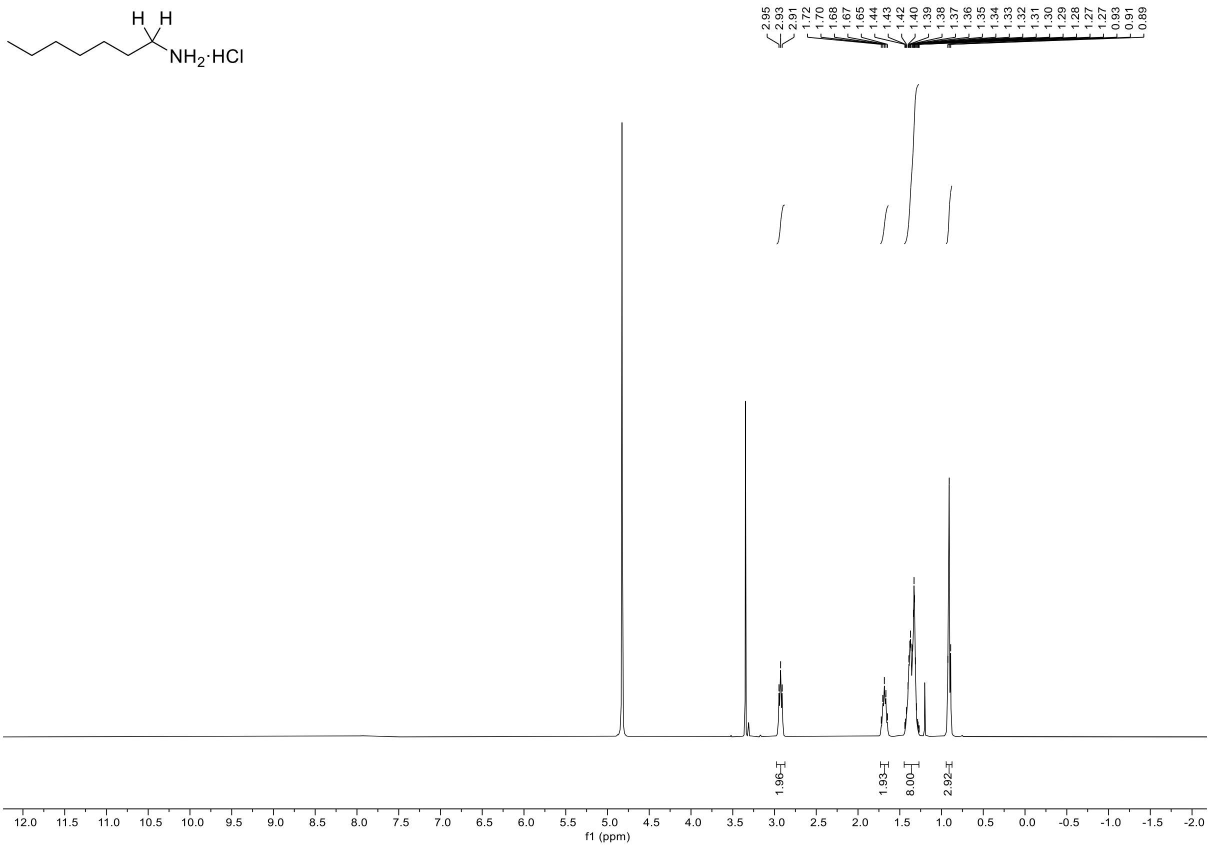

^{1}H NMR (400 MHz, methanol-*d*$_4$) of heptan-1-amine hydrochloride (**1a)**

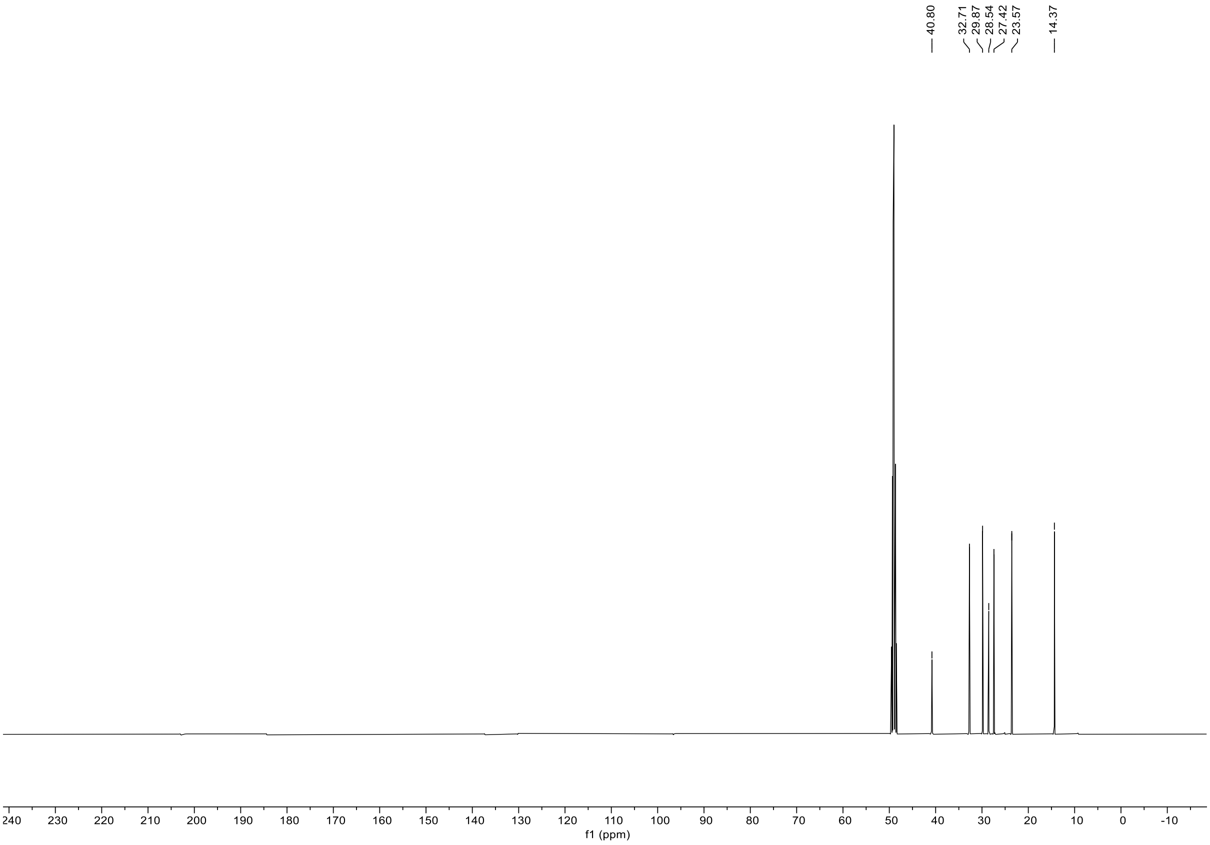

^{13}C NMR (126 MHz, methanol-*d*$_4$) of heptan-1-amine hydrochloride (**1a**)

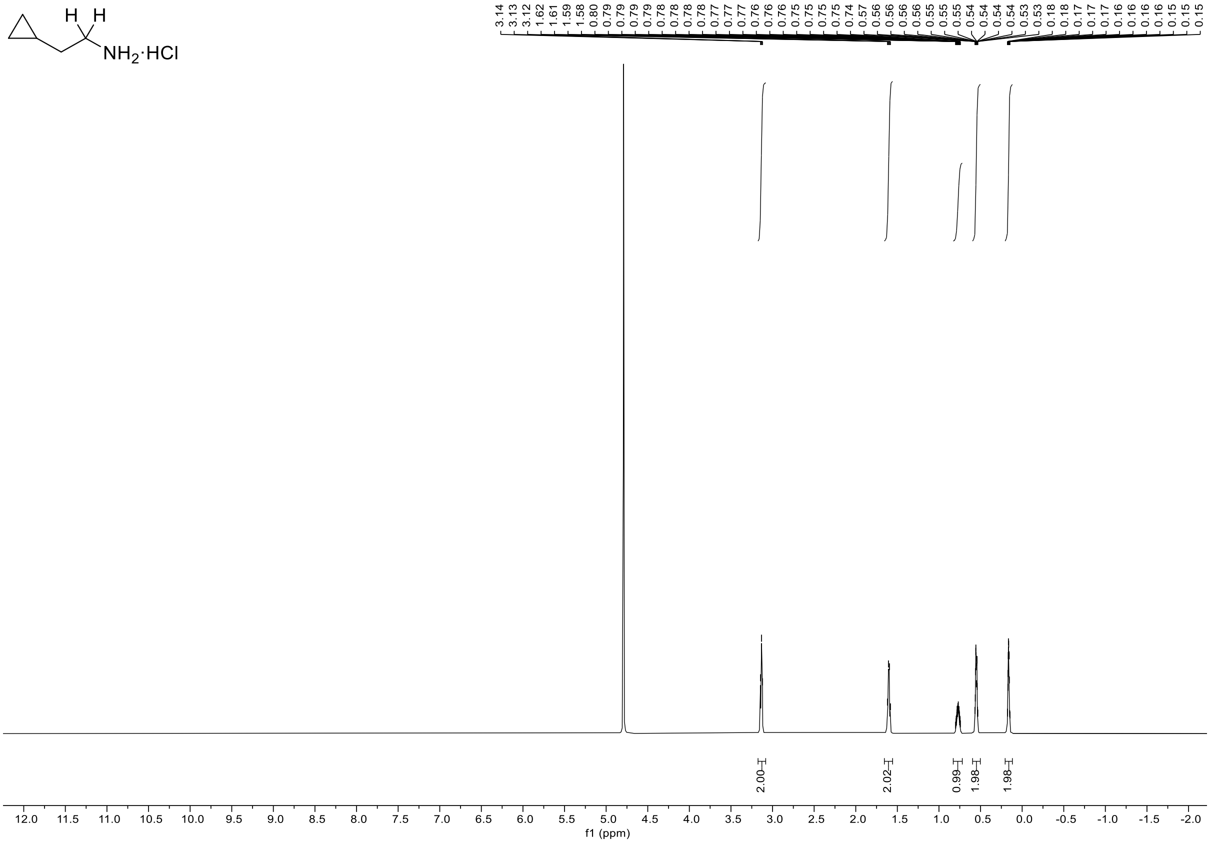

^{1}H NMR (600 MHz, D_2O) of 2-cyclopropylethanamine hydrochloride (**1b**)

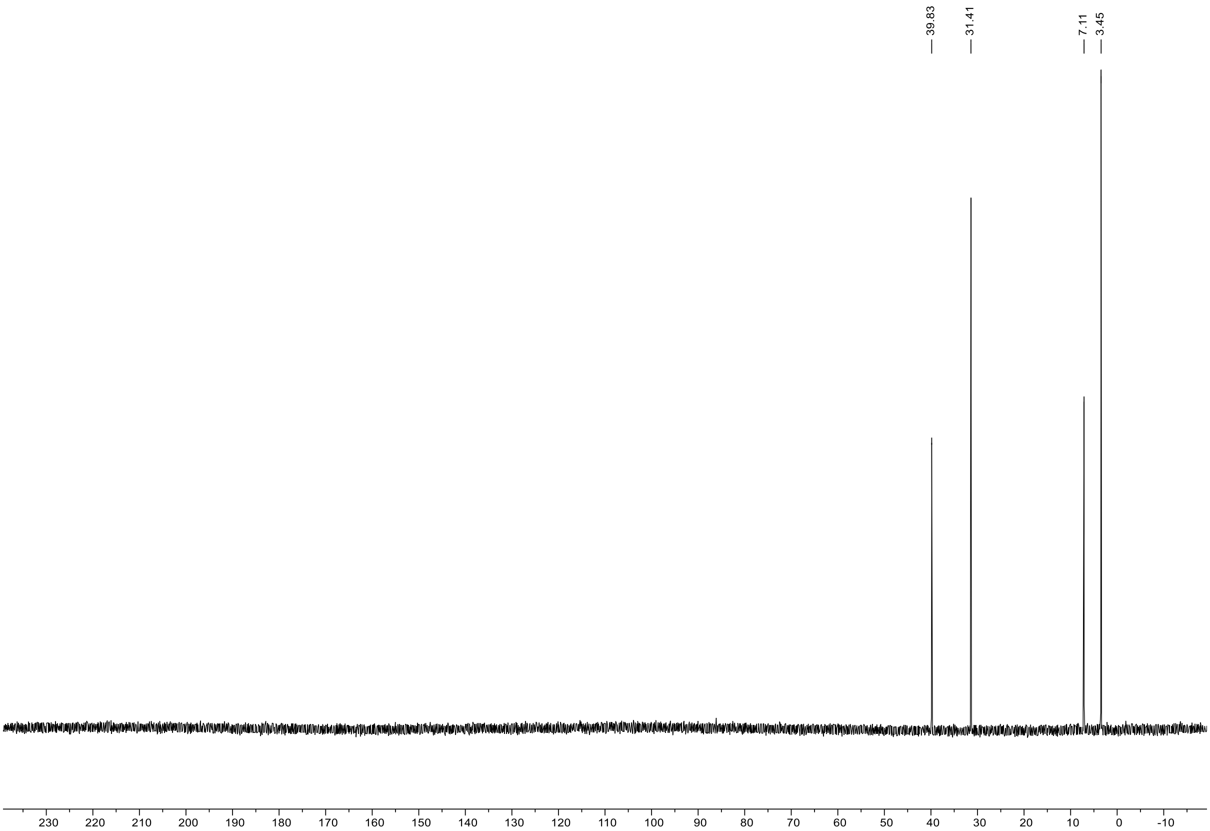

^{13}C NMR (151 MHz, D_2O) of 2-cyclopropylethanamine hydrochloride (**1b**)

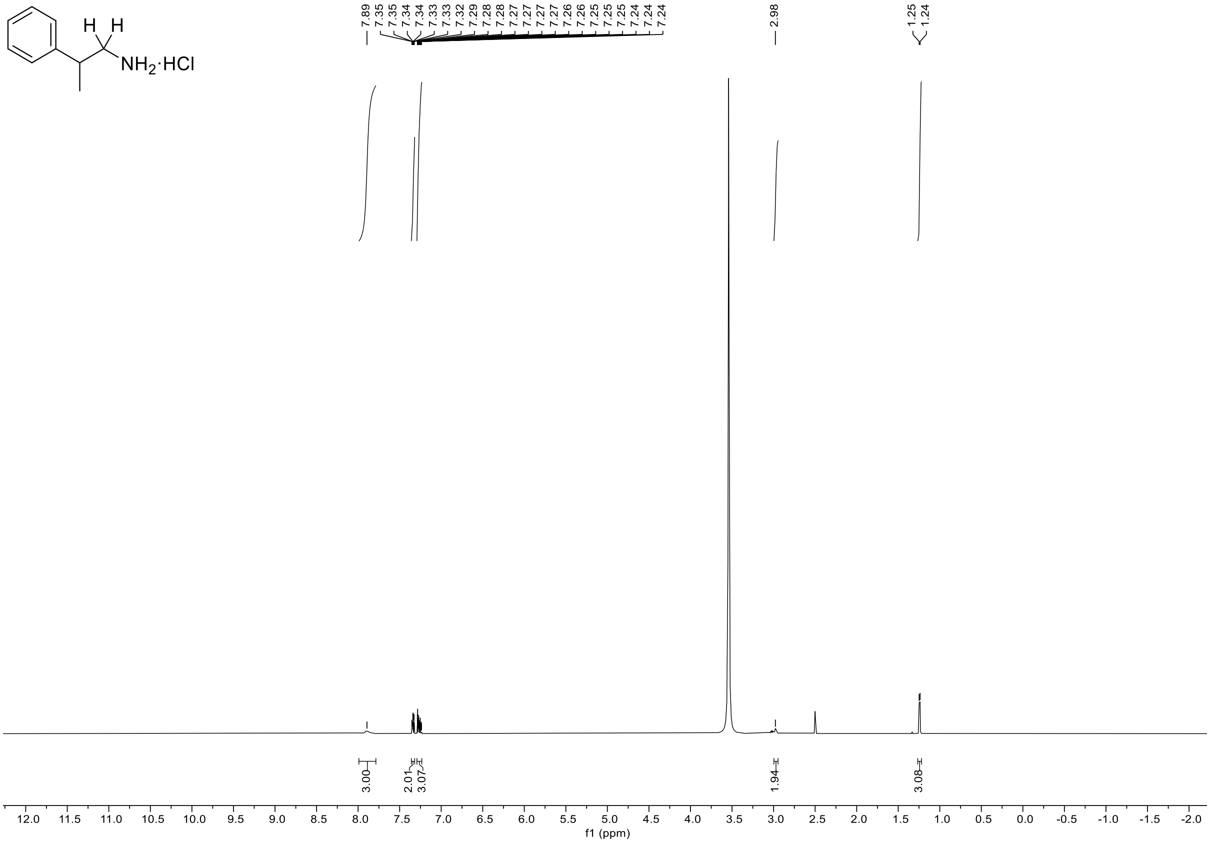

^{1}H NMR (600 MHz, DMSO-d_6) of *rac*-2-phenylpropan-1-amine hydrochloride (**1c**)

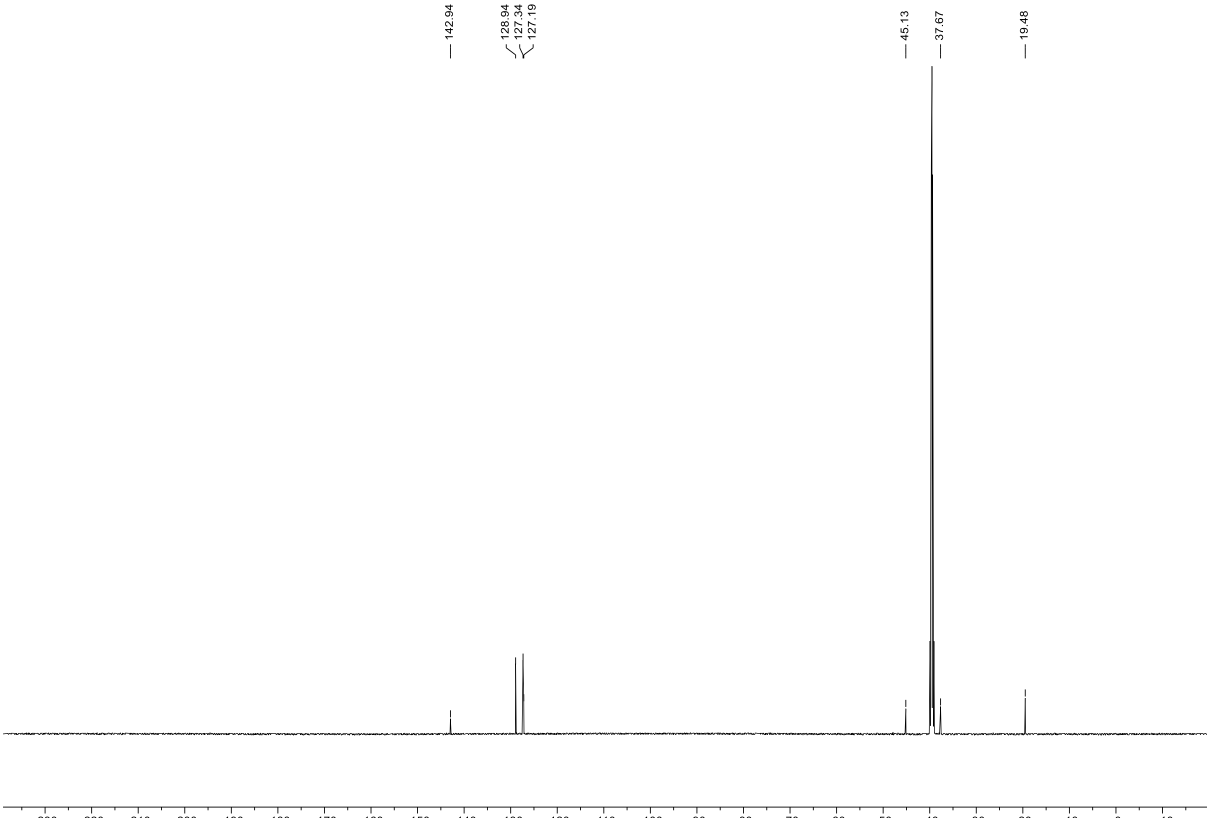

^{13}C NMR (151 MHz, DMSO-d_6) of *rac*-2-phenylpropan-1-amine hydrochloride (**1c**)

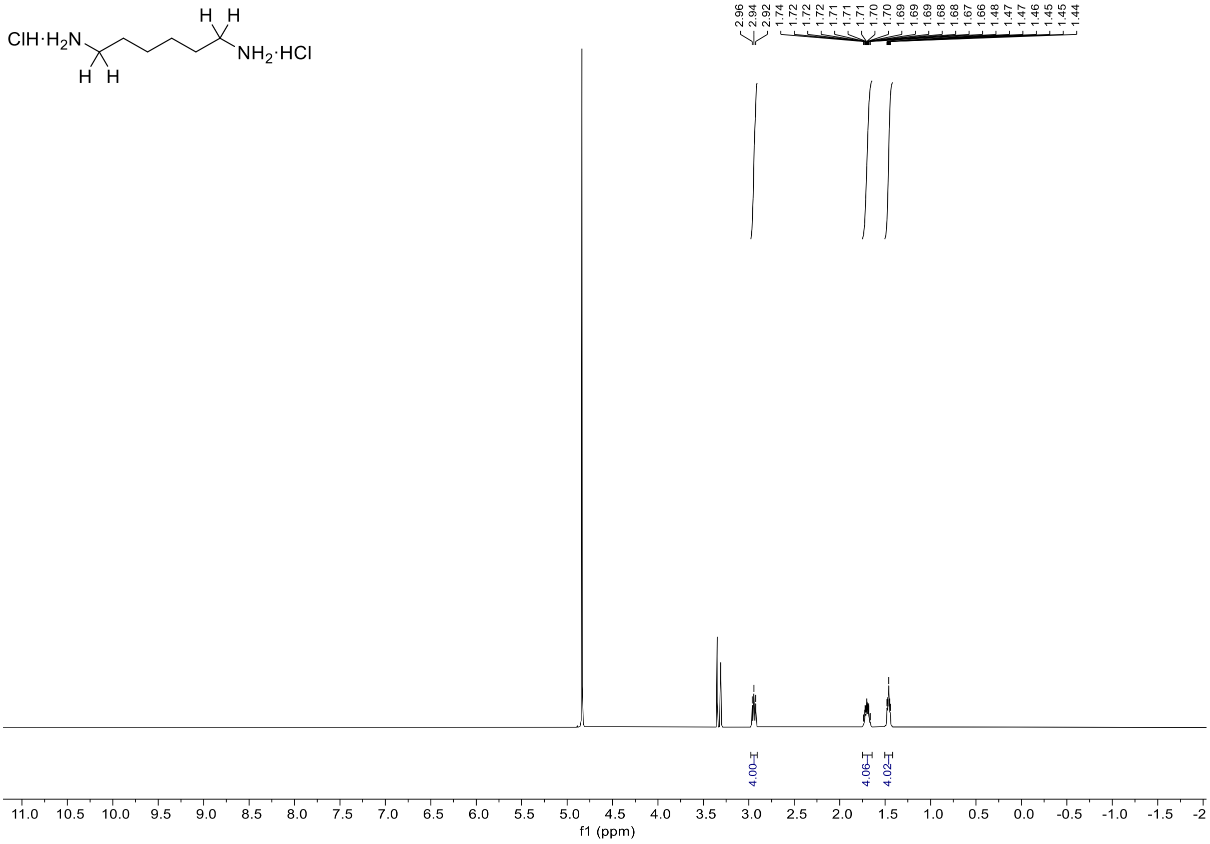

^{1}H NMR (400 MHz, methanol-d_4) of hexane-1,6-diamine dihydrochloride (**1d**)

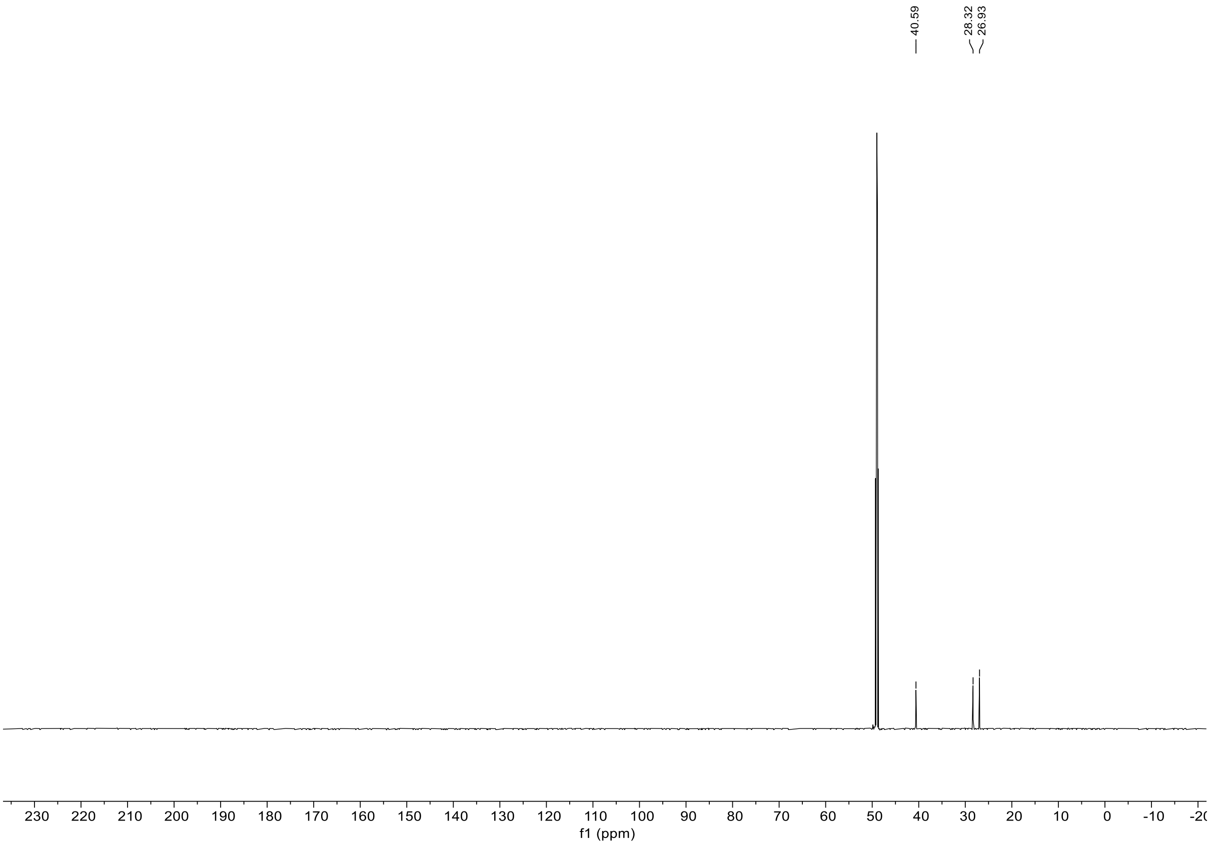

^{13}C NMR (151 MHz, methanol-d_4) of hexane-1,6-diamine dihydrochloride (**1d**)

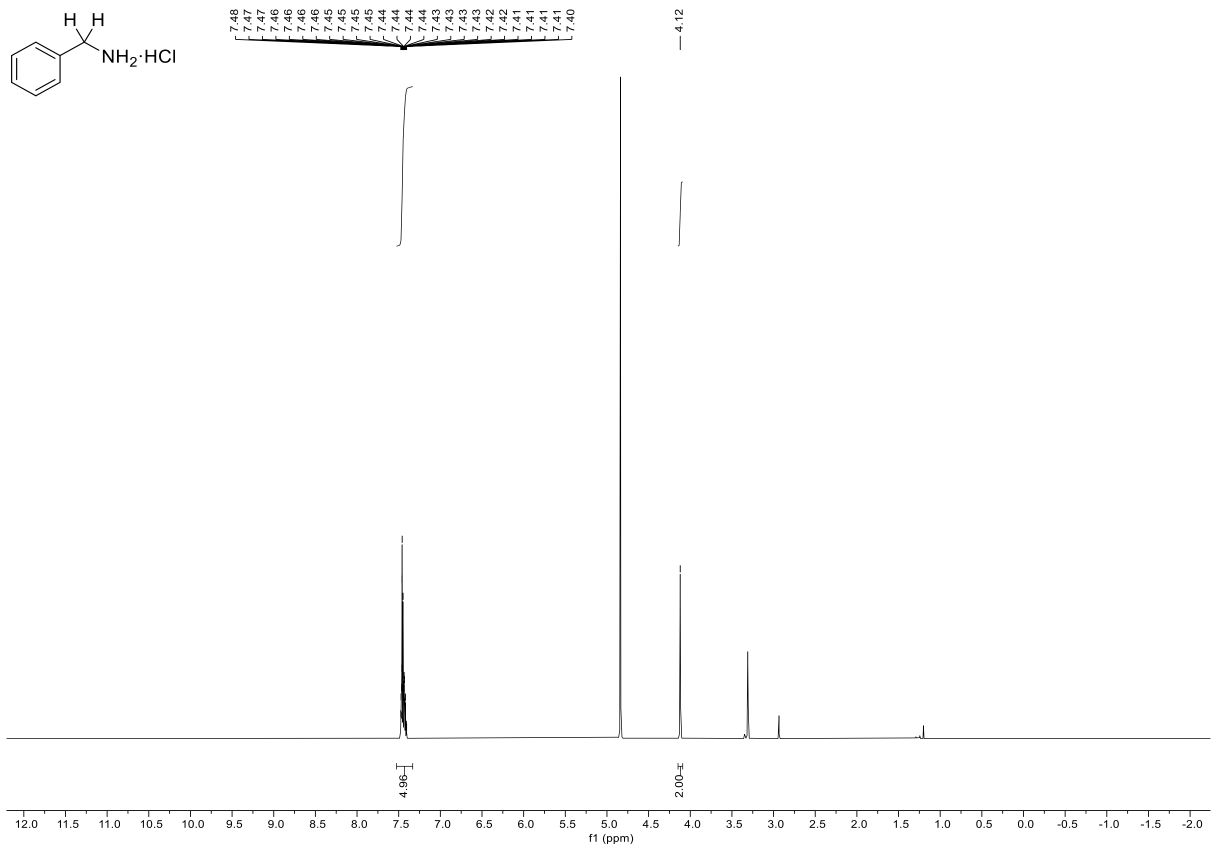

^{1}H NMR (600 MHz, methanol-d_4) of benzylamine hydrochloride (**1e**)

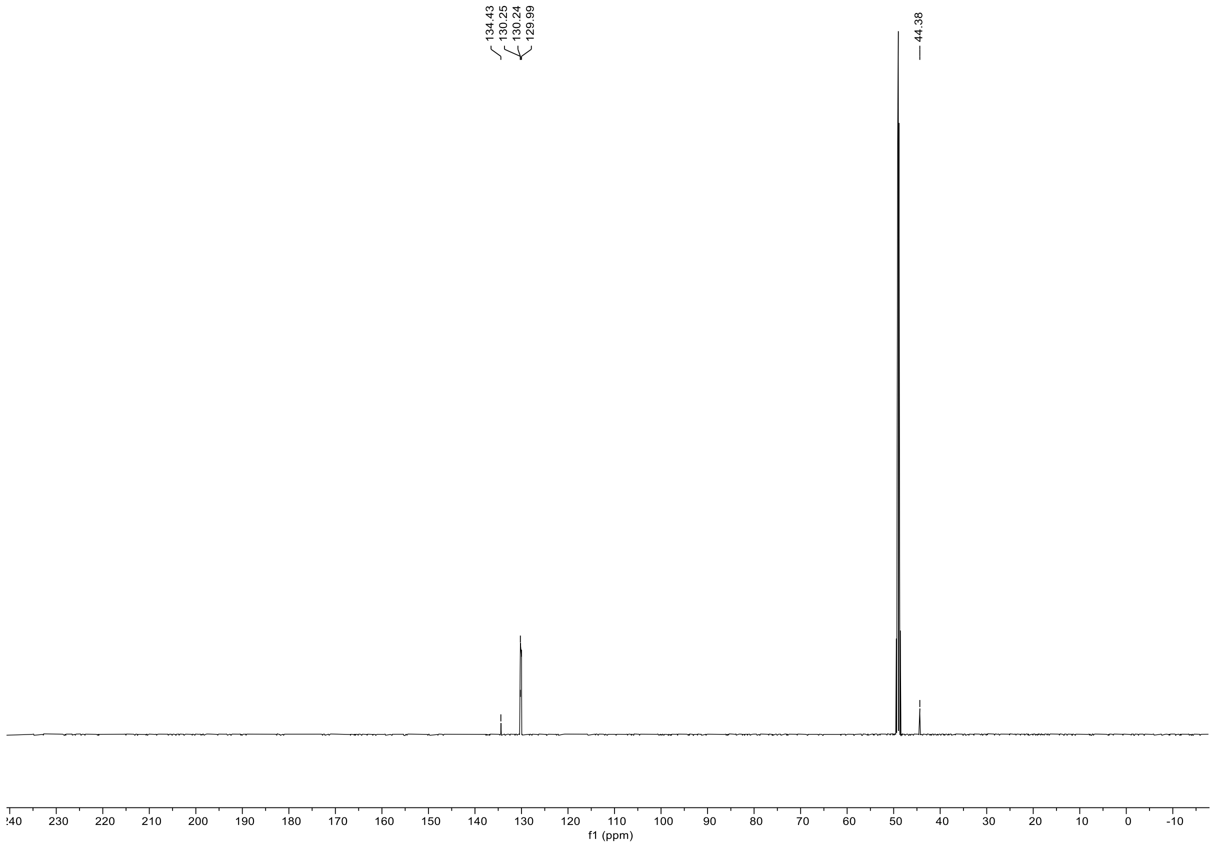

^{13}C NMR (151 MHz, methanol-d_4) of benzylamine hydrochloride (**1e**)

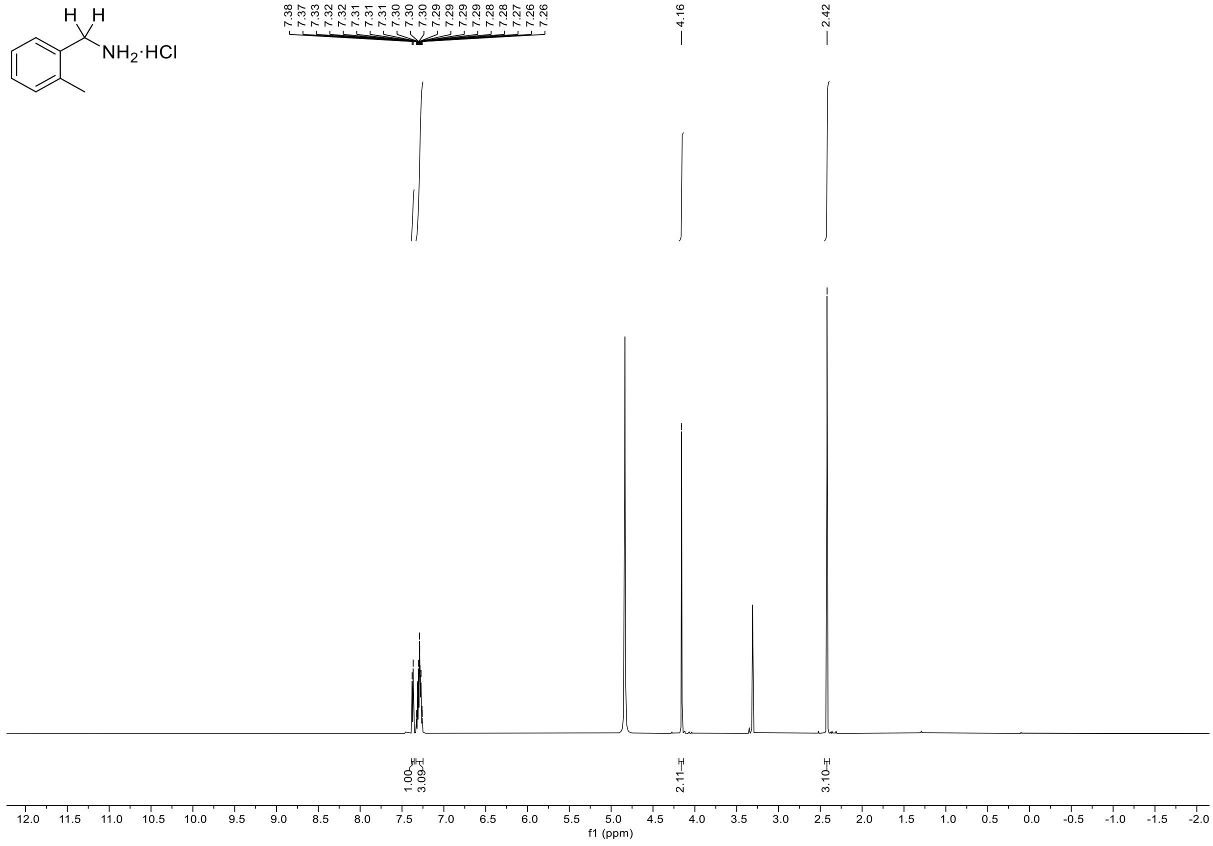

^{1}H NMR (600 MHz, methanol-d_4) of 2-methylbenzylamine hydrochloride (**1f**)

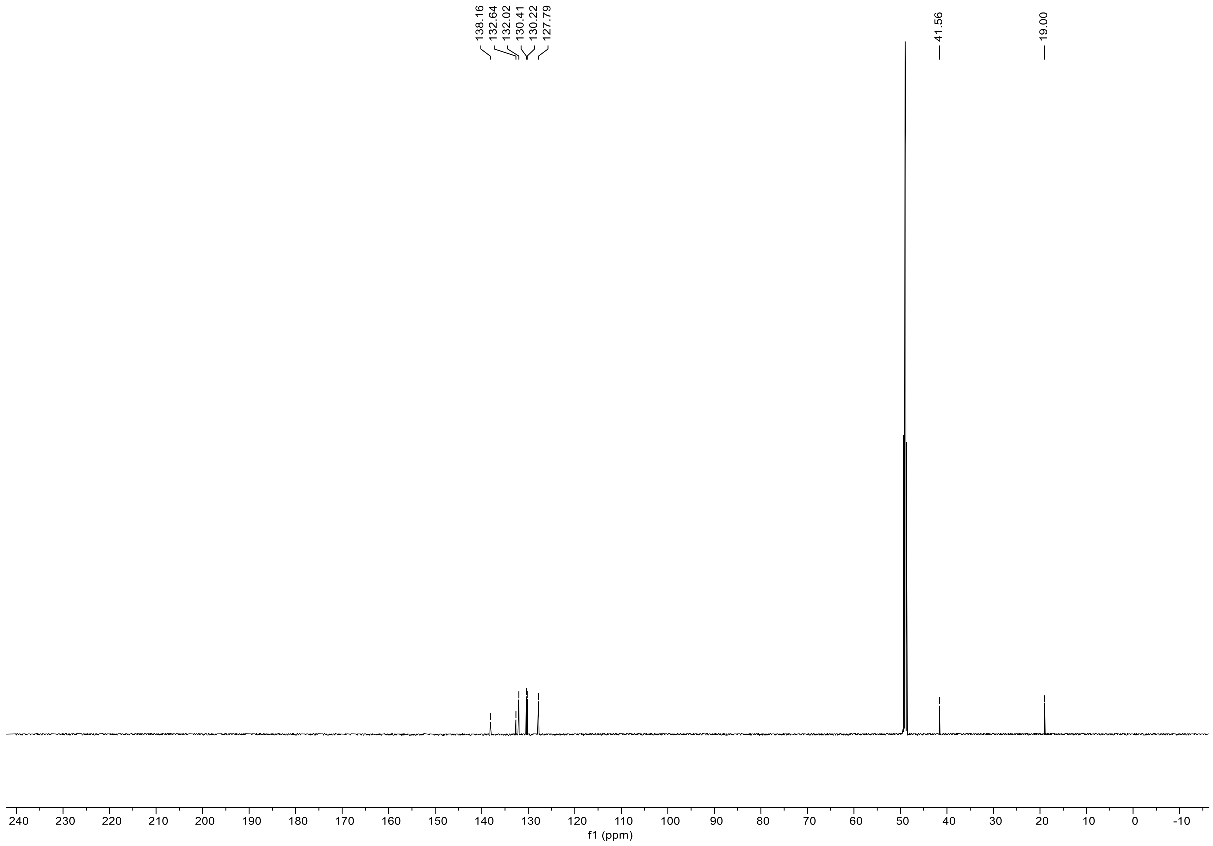

^{13}C NMR (151 MHz, methanol-d_4) of 2-methylbenzylamine hydrochloride (**1f**)

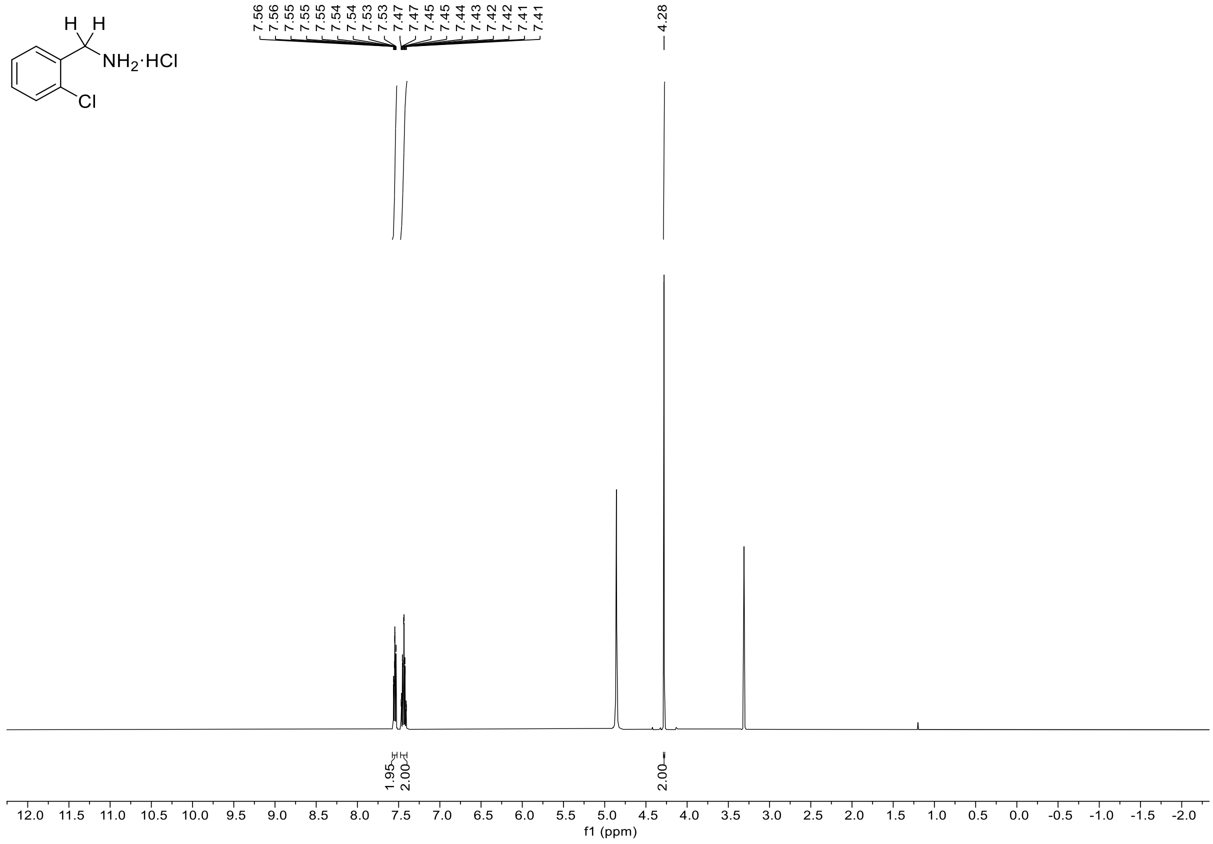

^{1}H NMR (500 MHz, methanol-d_4) of 2-chlorobenzylamine hydrochloride (**1g**)

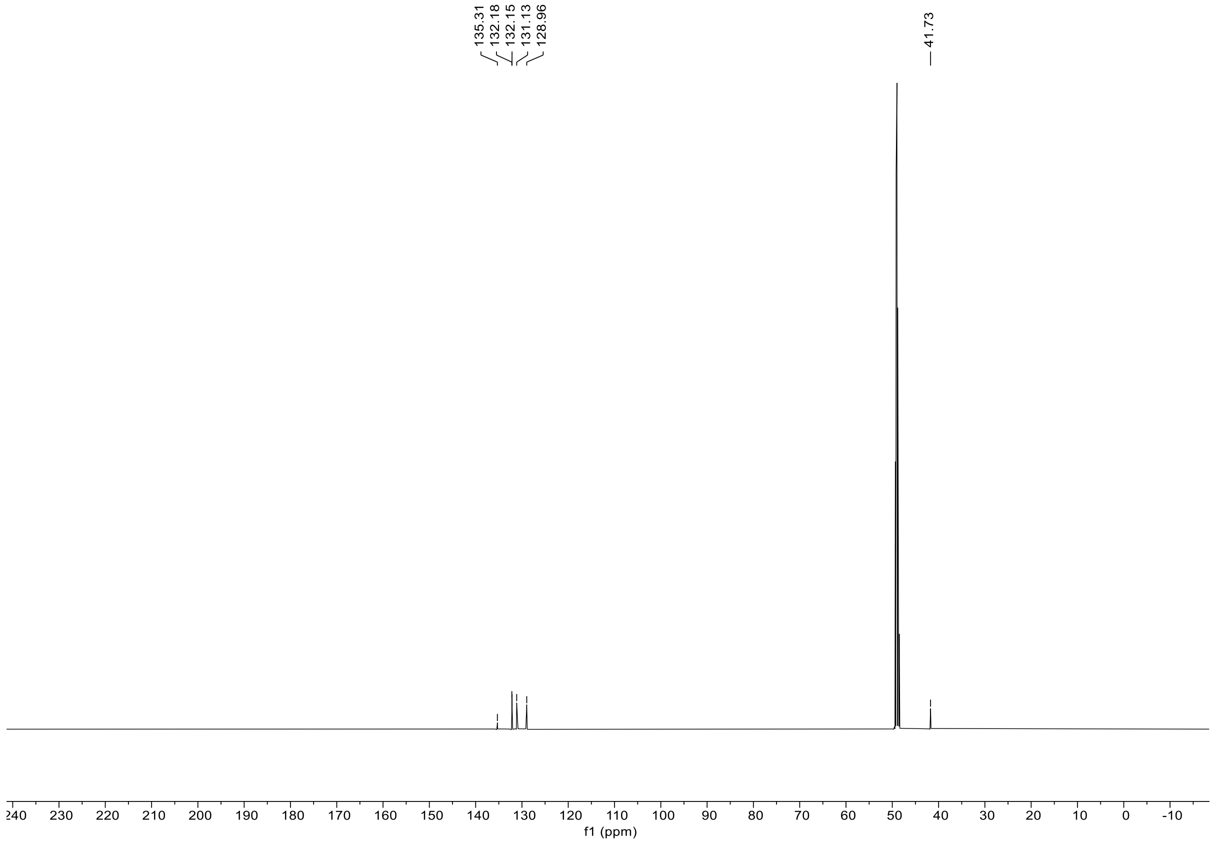

^{13}C NMR (126 MHz, methanol-d_4) of 2-chlorobenzylamine hydrochloride (**1g**)

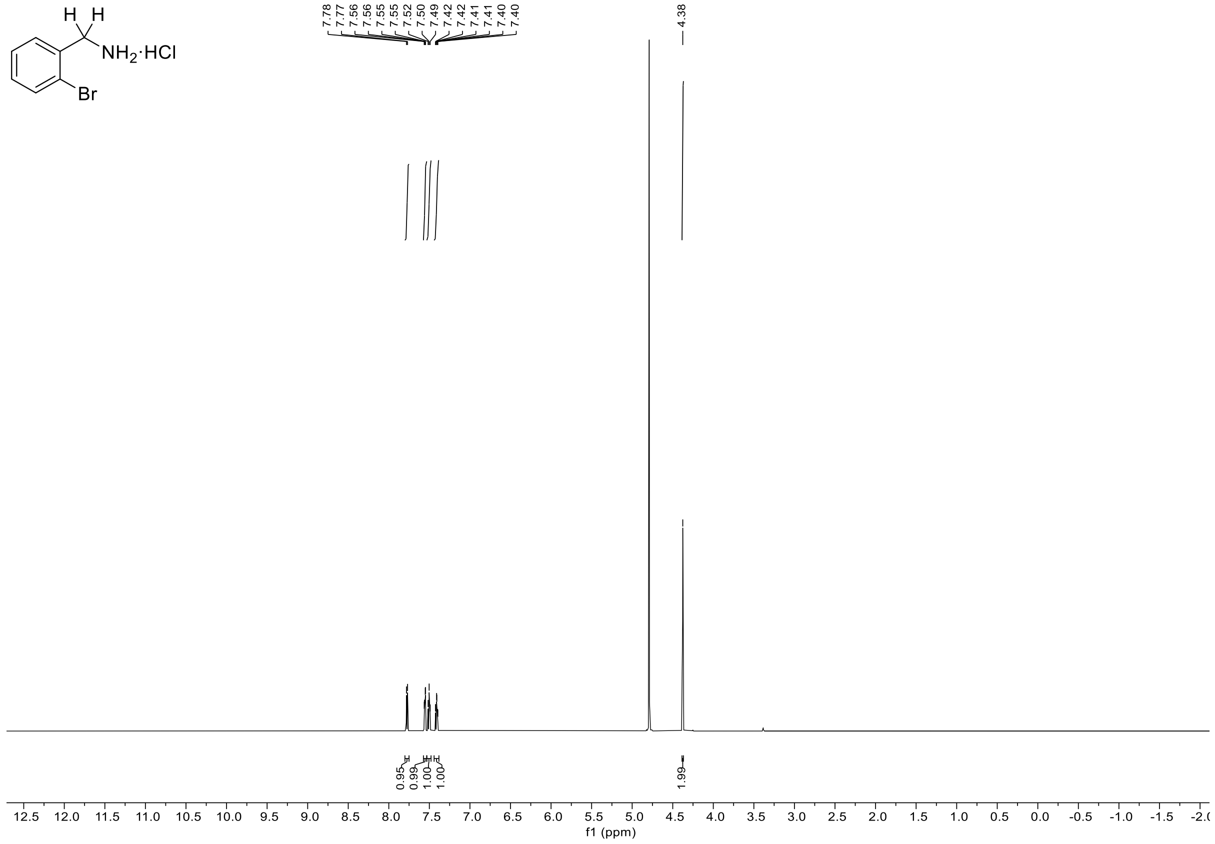

^{1}H NMR (600 MHz, D_2O) of 2-bromobenzylamine hydrochloride (**1h**)

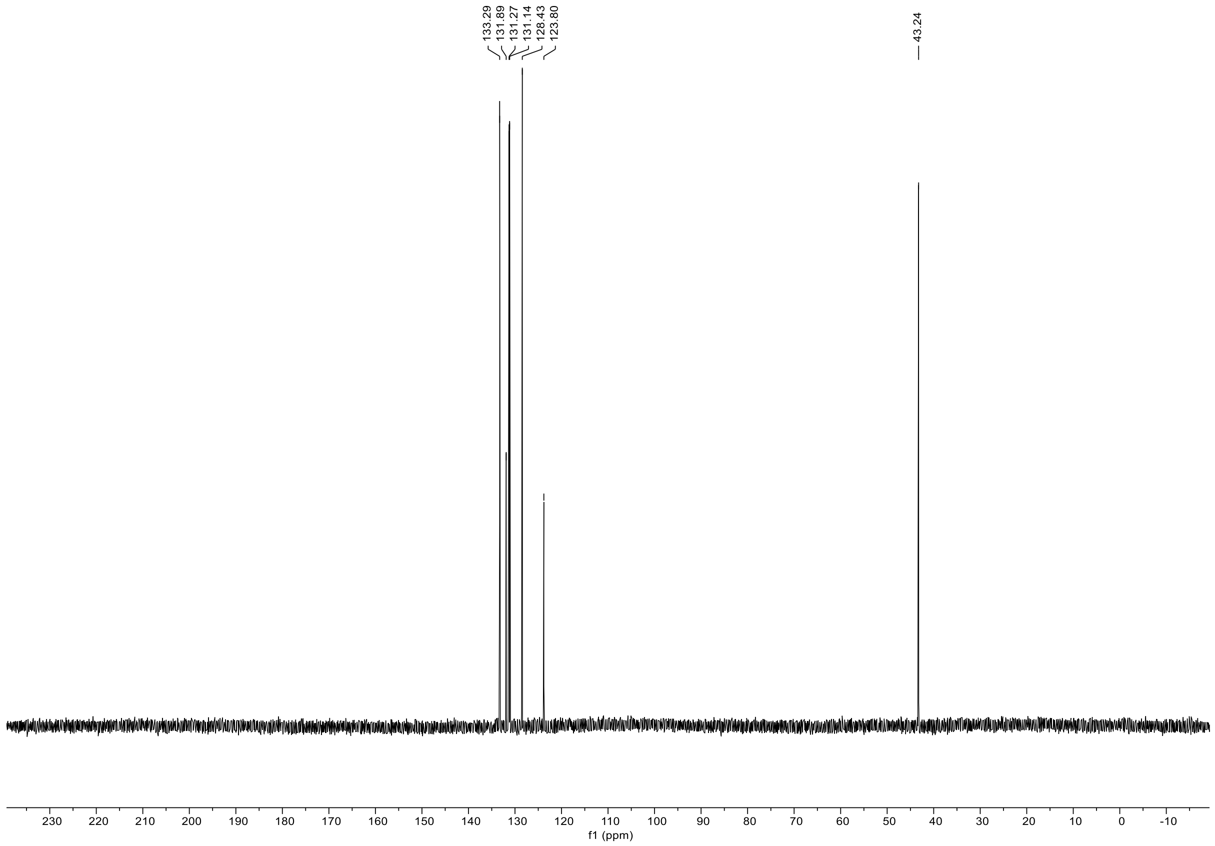

^{13}C NMR (151 MHz, D_2O) of 2-bromobenzylamine hydrochloride (**1h**)

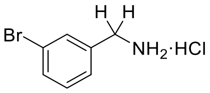

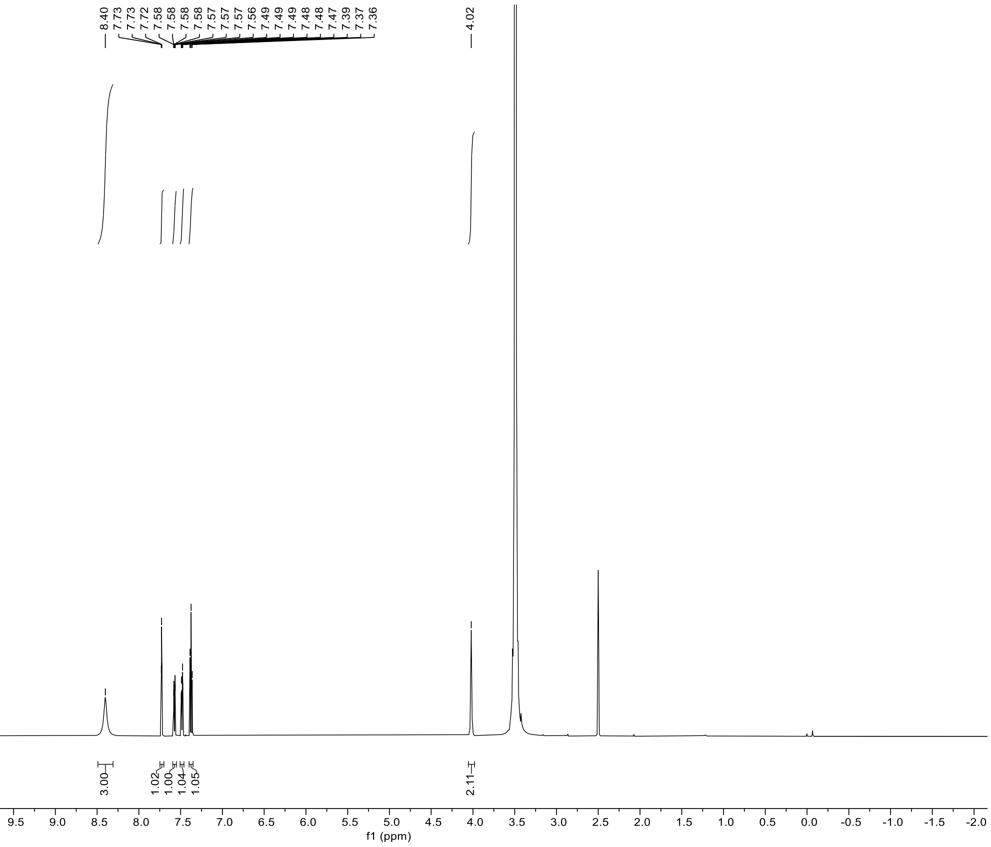

^{1}H NMR (600 MHz, DMSO-d_6) of 3-bromobenzylamine hydrochloride (**1i**)

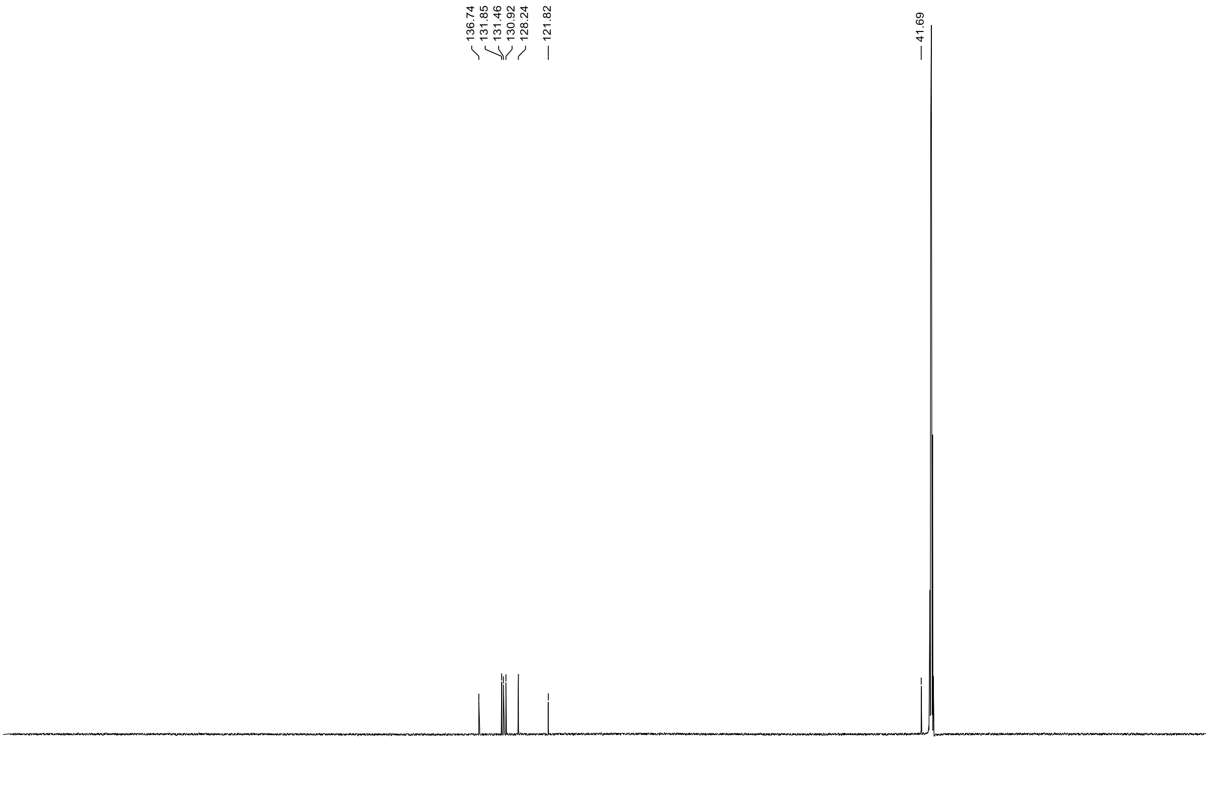

^{13}C NMR (151 MHz, DMSO-d_6) of 3-bromobenzylamine hydrochloride (**1i**)

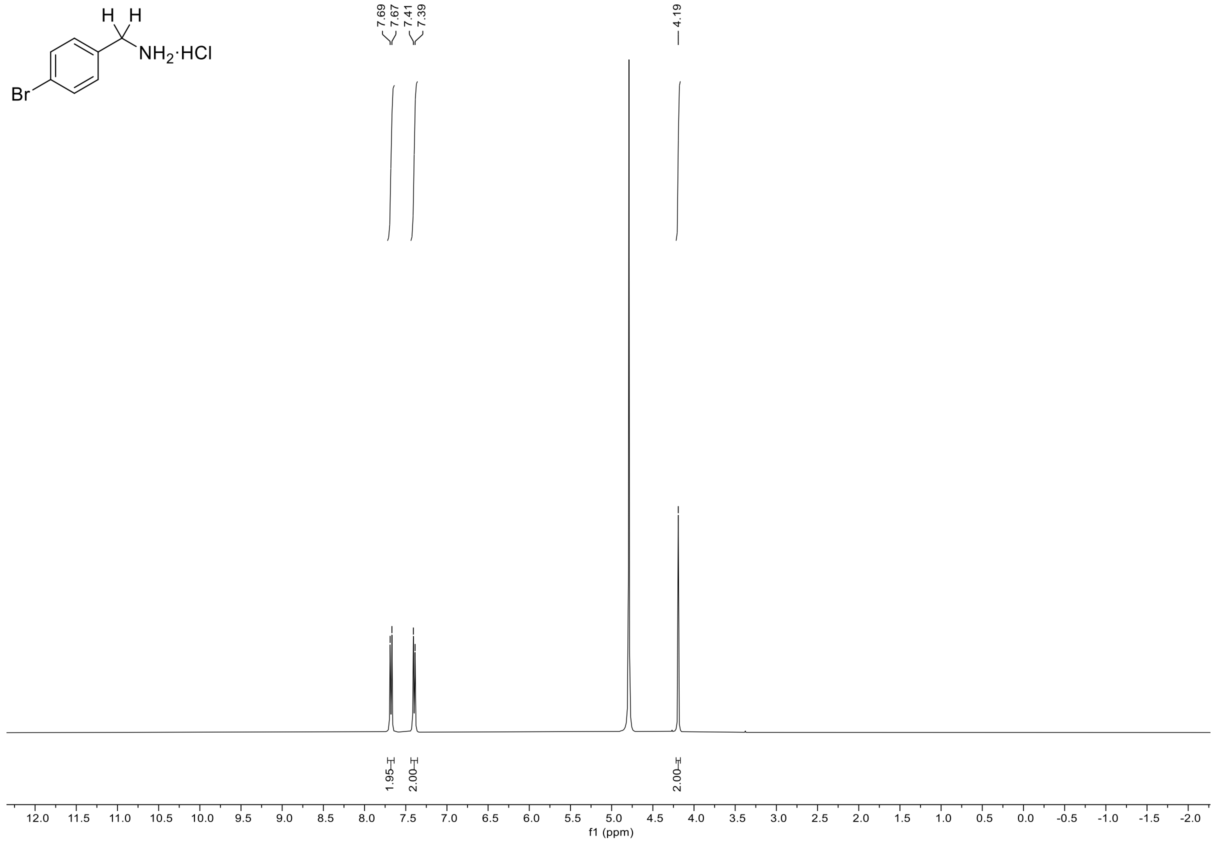

^{1}H NMR (400 MHz, D_2O) of 4-bromobenzylamine hydrochloride (**1j**)

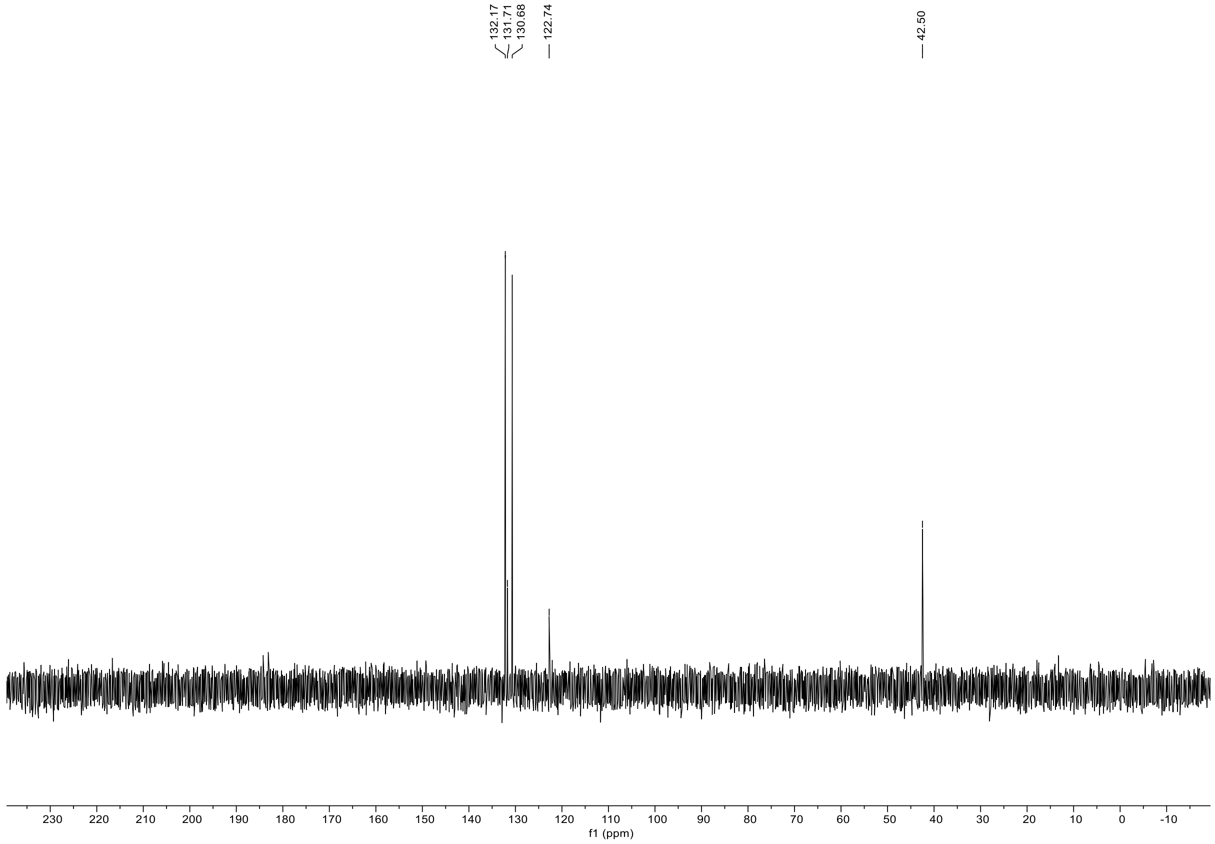

^{13}C NMR (101 MHz, D_2O) of 4-bromobenzylamine hydrochloride (**1j**)

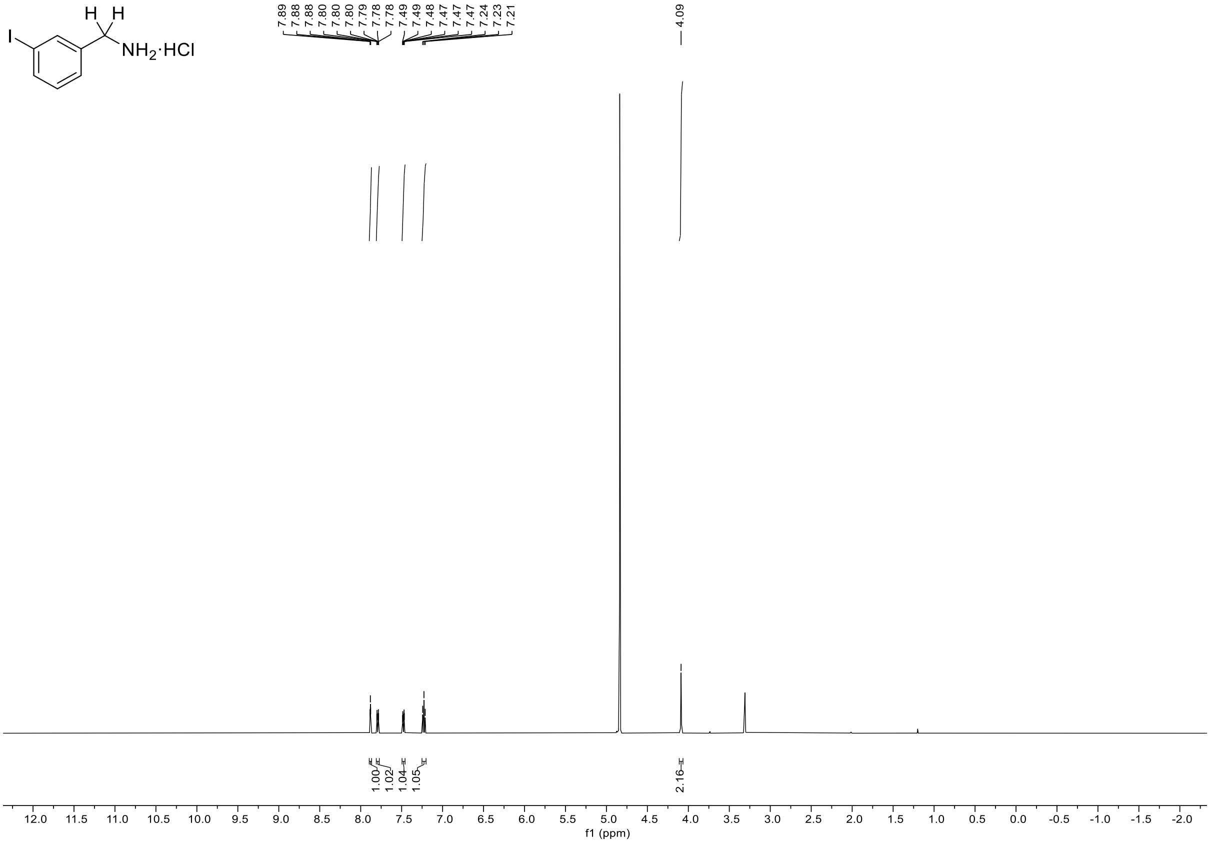

^{1}H NMR (500 MHz, methanol-d_4) of 3-iodobenzylamine hydrochloride (**1k**)

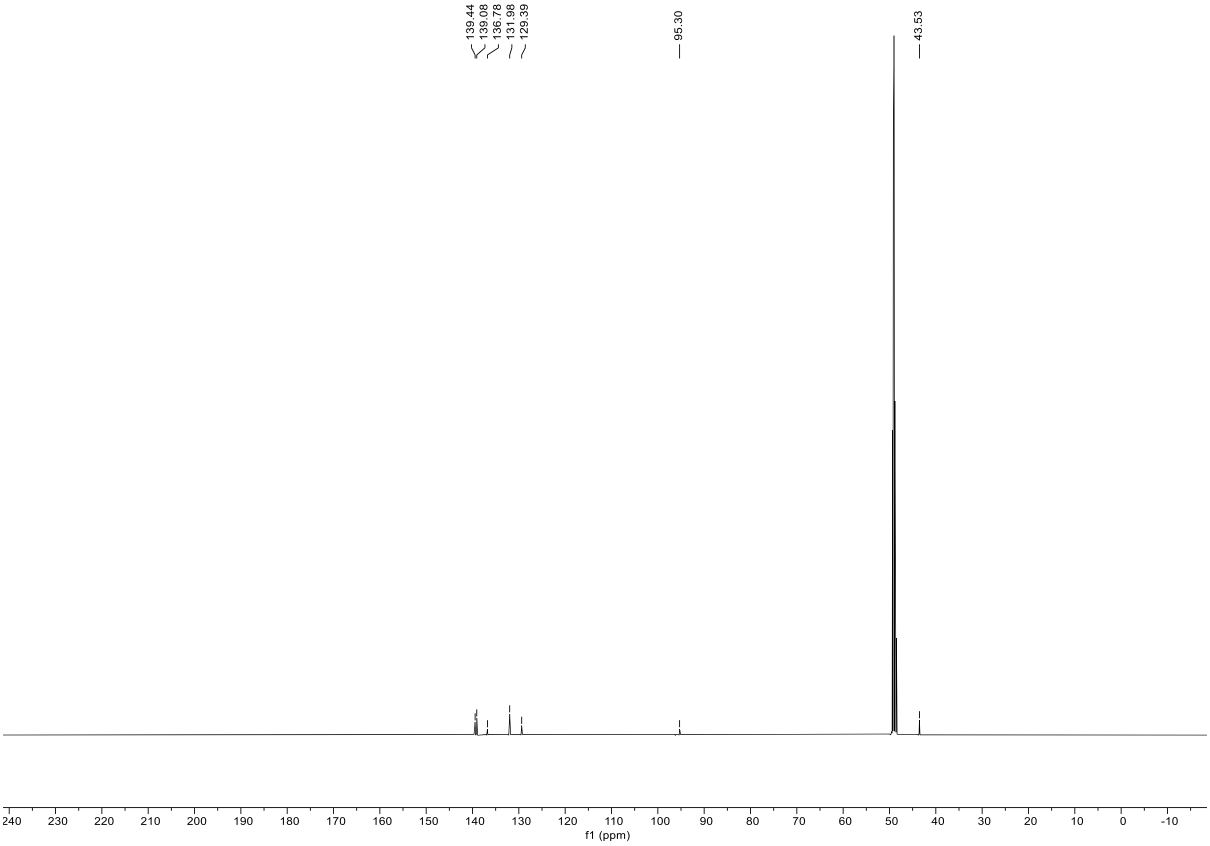

^{13}C NMR (126 MHz, methanol-d_4) of 3-iodobenzylamine hydrochloride (**1k**)

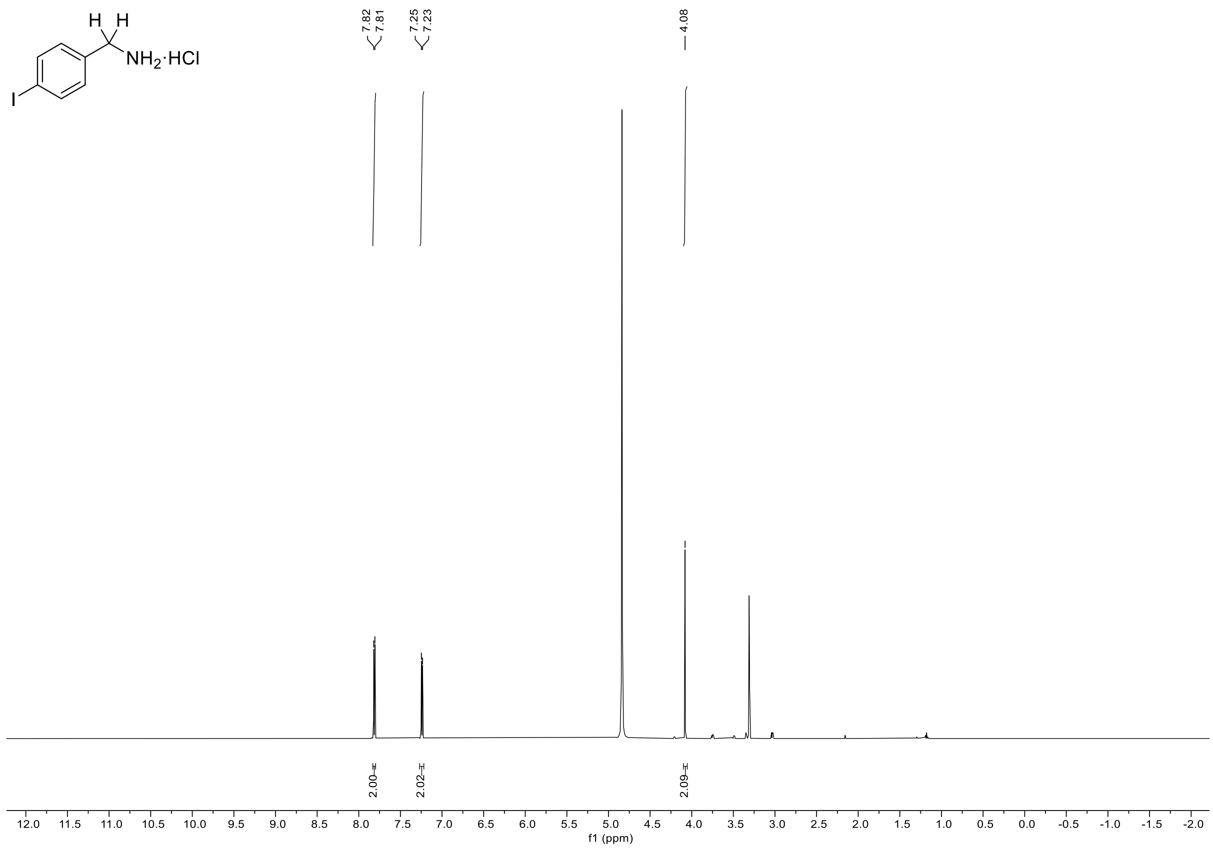

^{1}H NMR (600 MHz, methanol-*d*4) of 4-iodobenzylamine hydrochloride (**1l**)

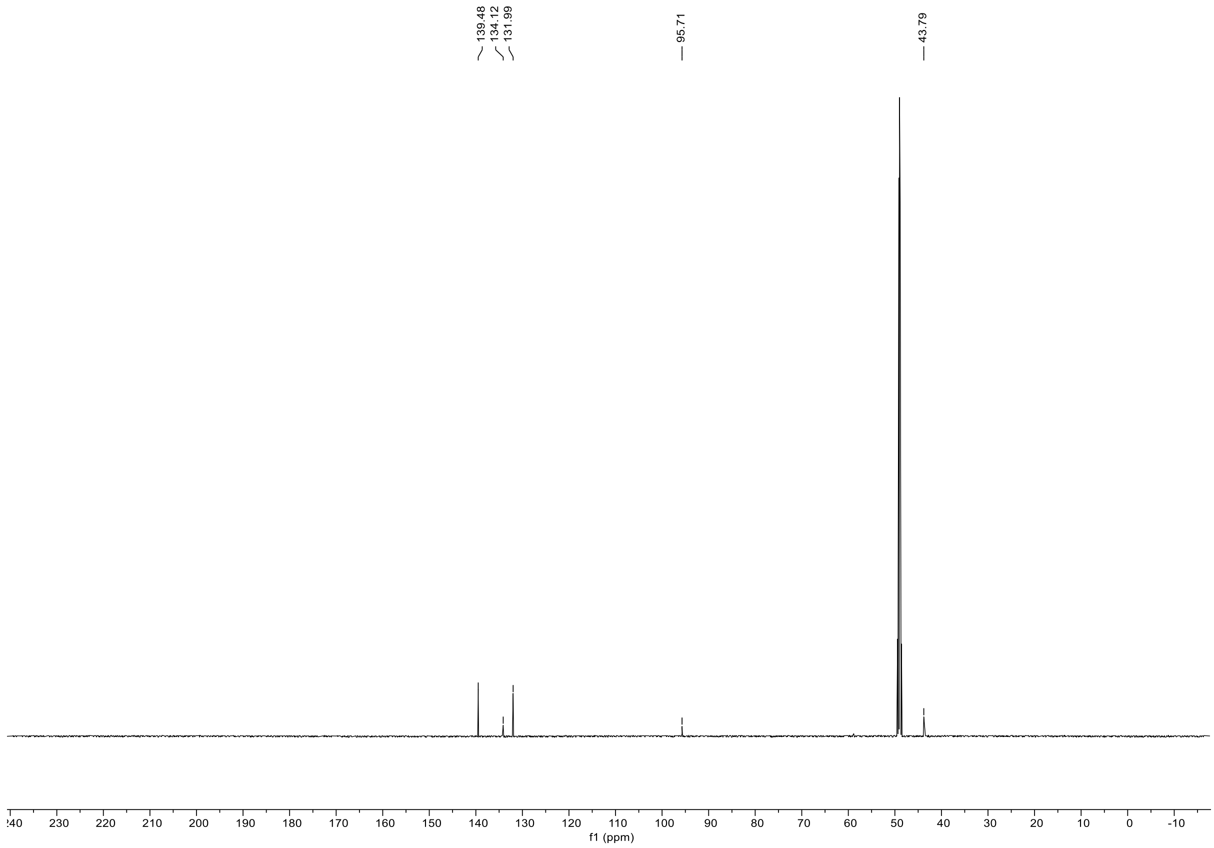

^{13}C NMR (151 MHz, methanol-*d*4) of 4-iodobenzylamine hydrochloride (**1l**)

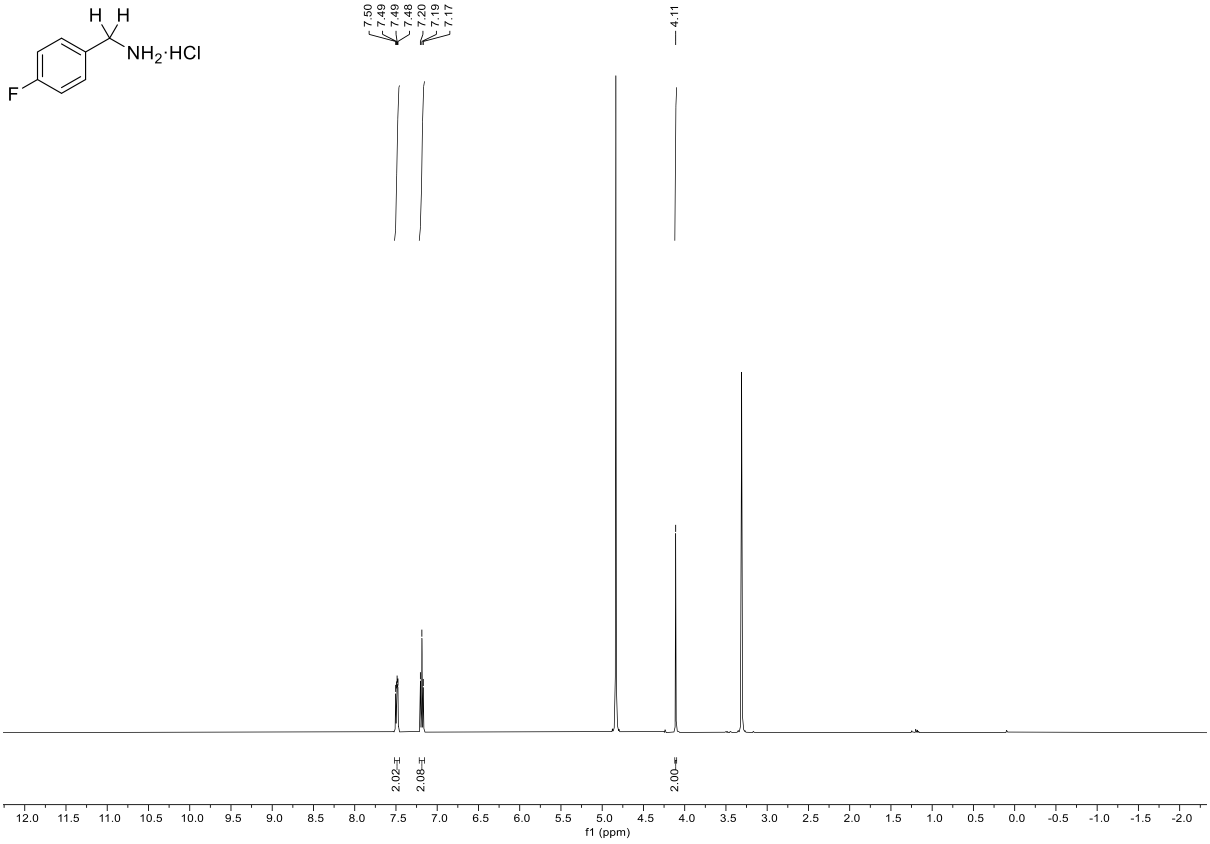

^{1}H NMR (500 MHz, methanol-d_4) of 4-fluorobenzylamine hydrochloride (**1m**)

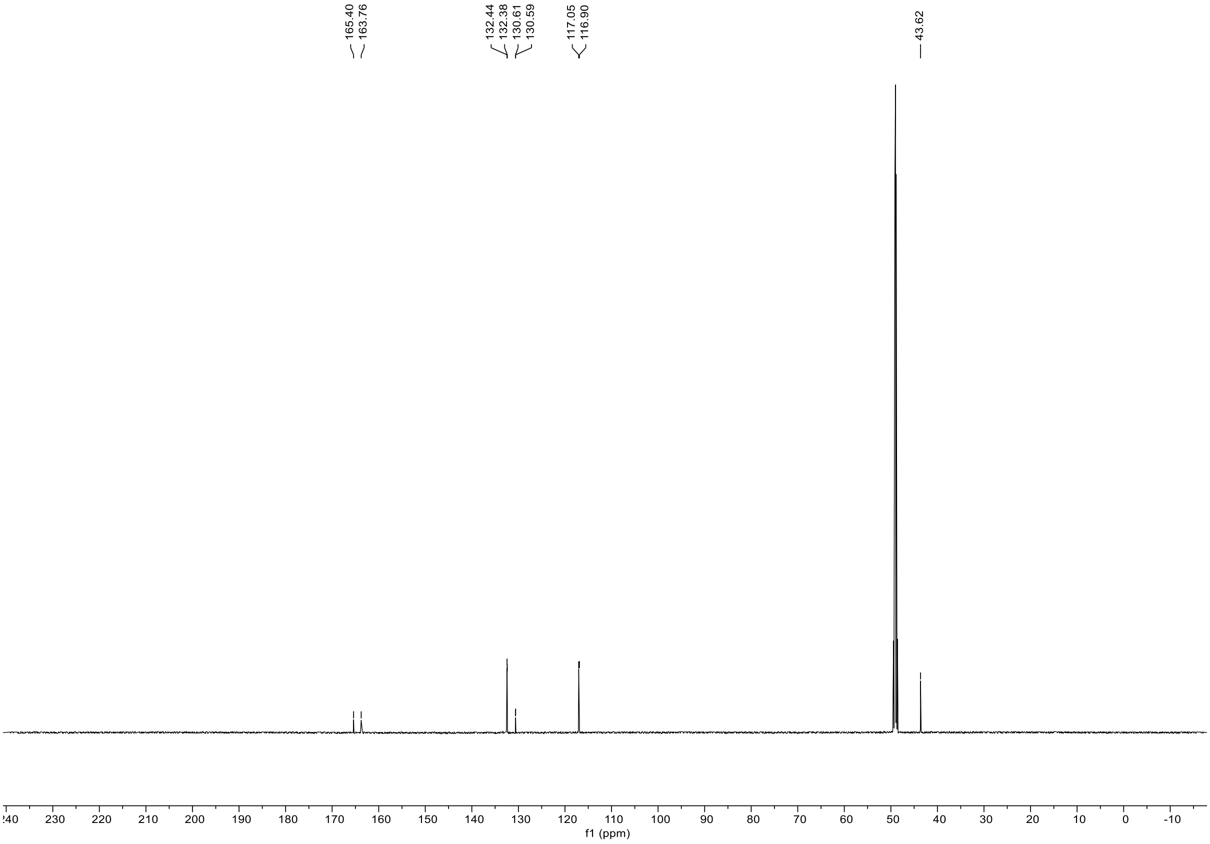

^{13}C NMR (151 MHz, methanol-d_4) of 4-fluorobenzylamine hydrochloride (**1m**)

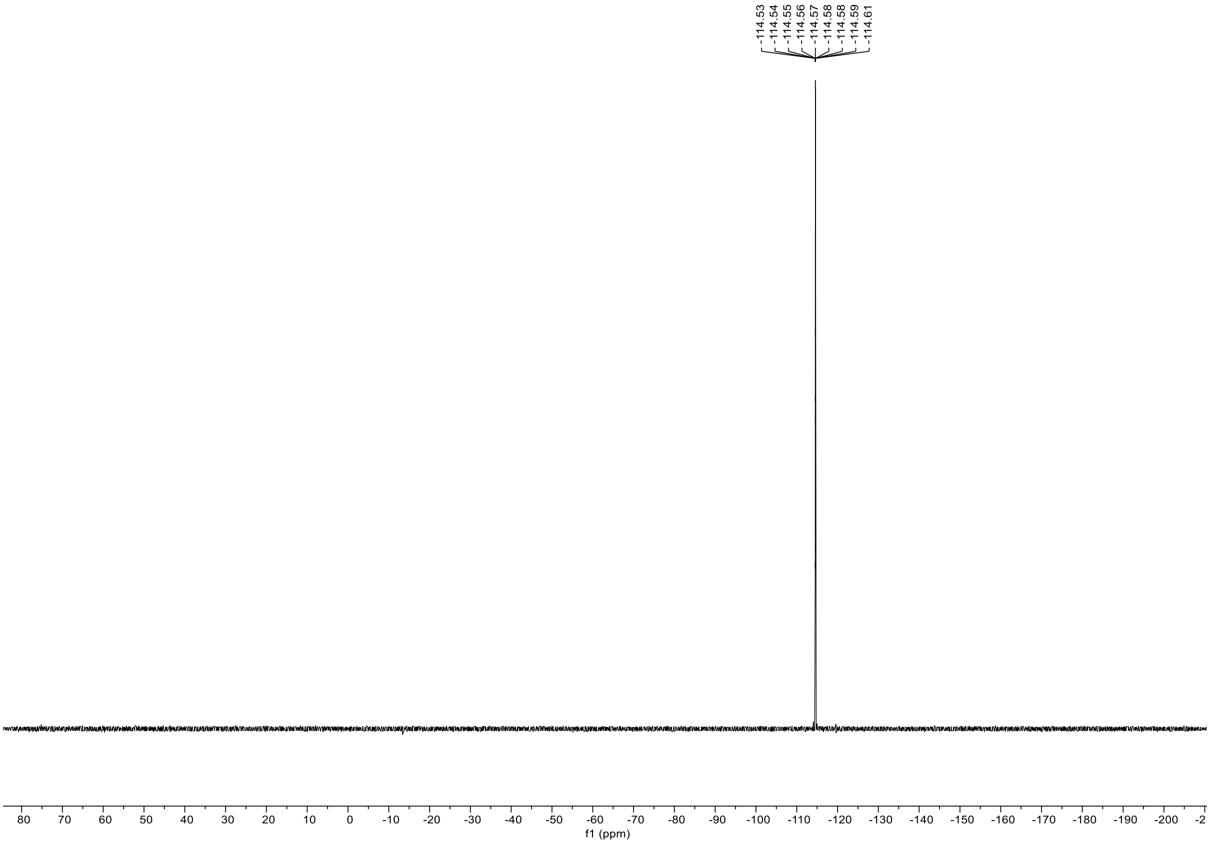

^{19}F NMR (376 MHz, methanol-d_4) of 4-fluorobenzylamine hydrochloride (**1m**)

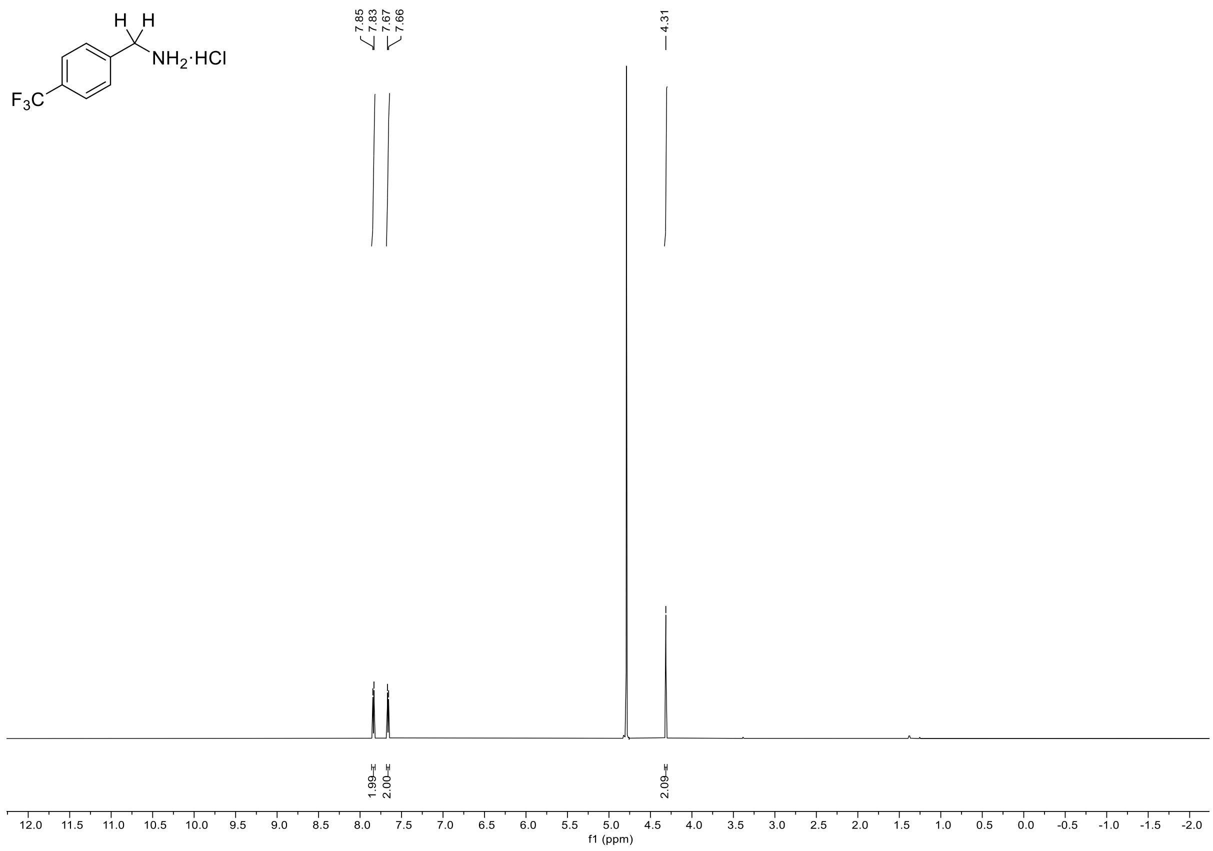

^{1}H NMR (600 MHz, D_2O) of 4-(trifluoromethyl)benzylamine hydrochloride (**1n**)

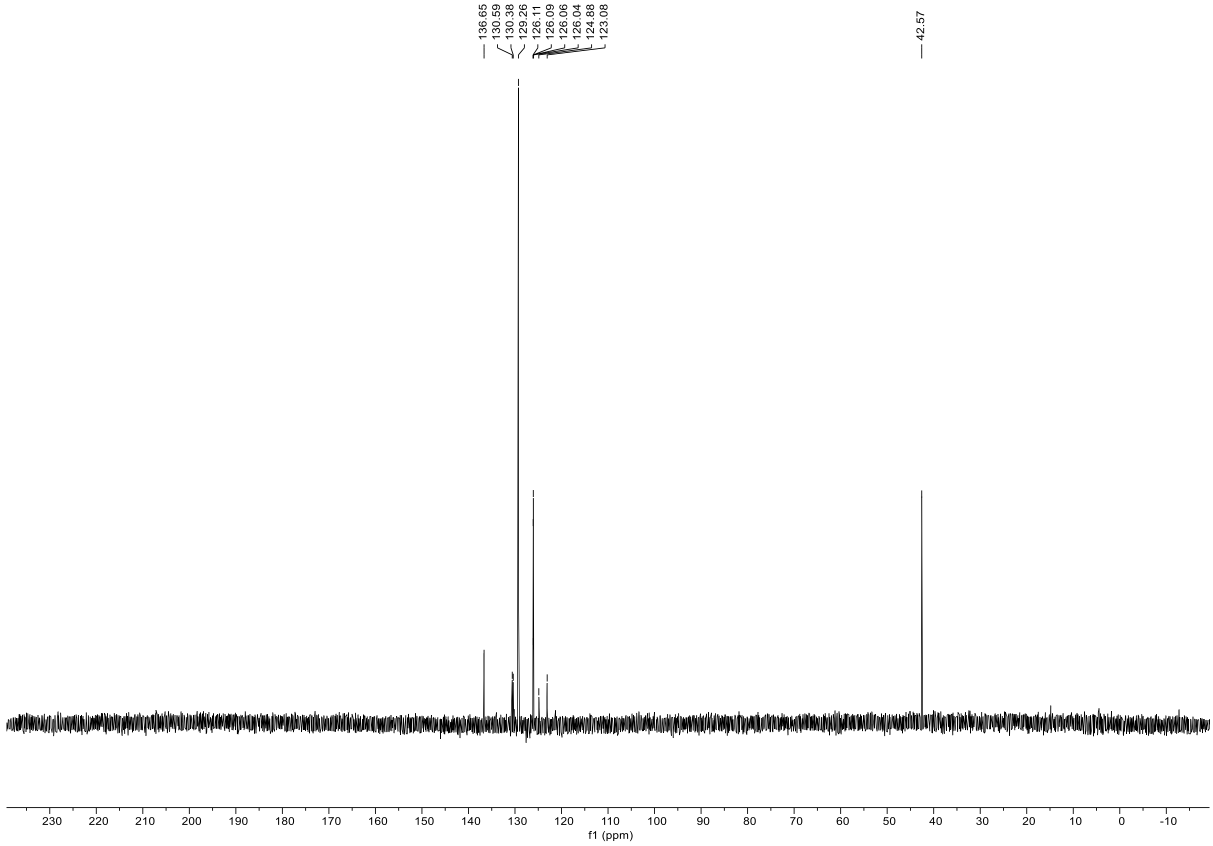

^{13}C NMR (151 MHz, D_2O) of 4-(trifluoromethyl)benzylamine hydrochloride (**1n**)

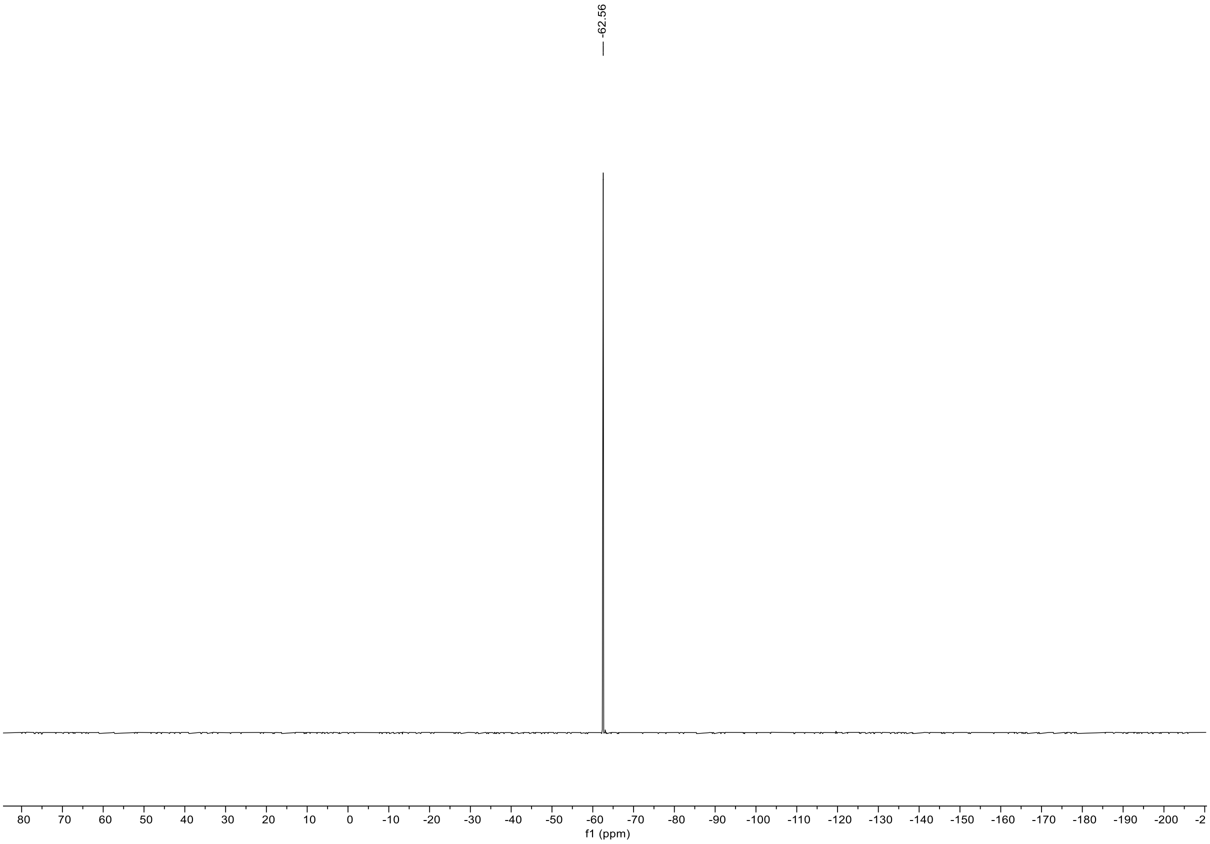

^{19}F NMR (376 MHz, D_2O) of 4-(trifluoromethyl)benzylamine hydrochloride (**1n**)

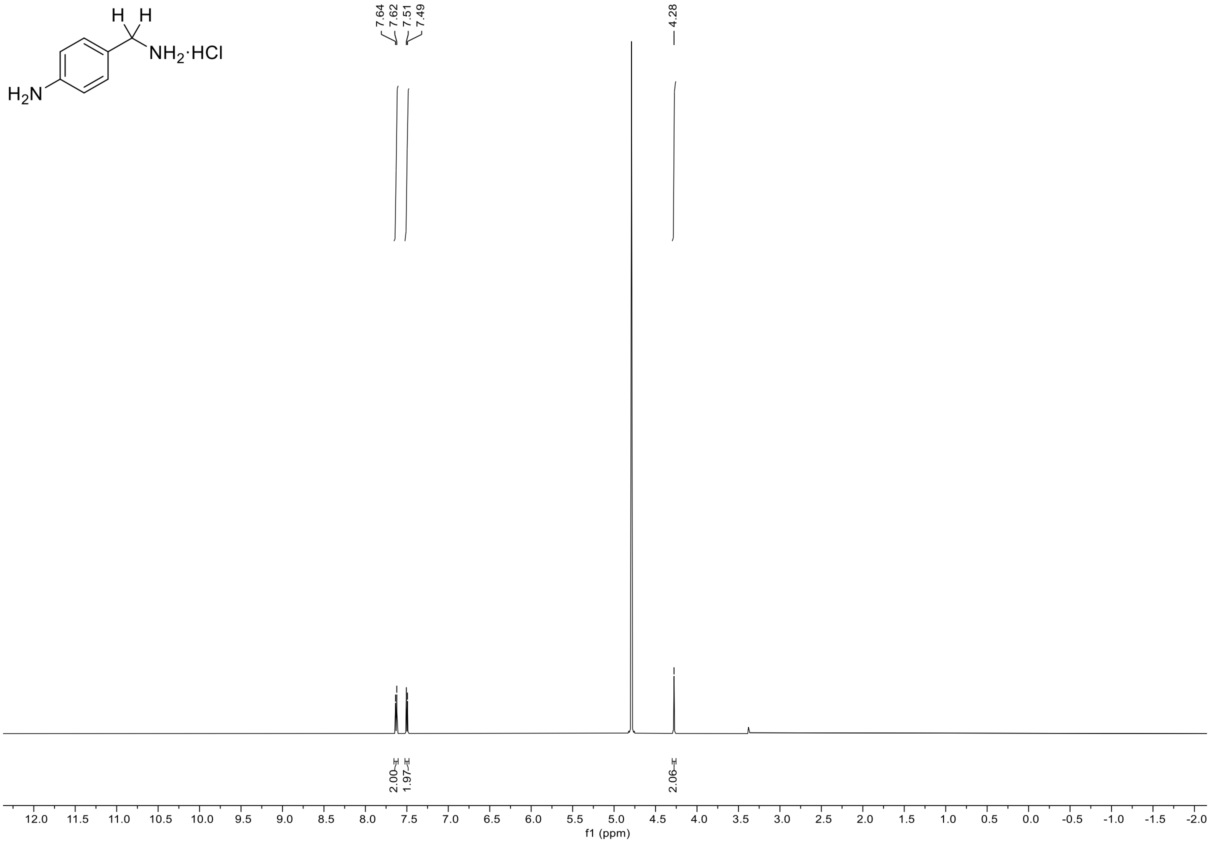

^{1}H NMR (600 MHz, D_2O) of 4-aminobenzylamine dihydrochloride (**1o**)

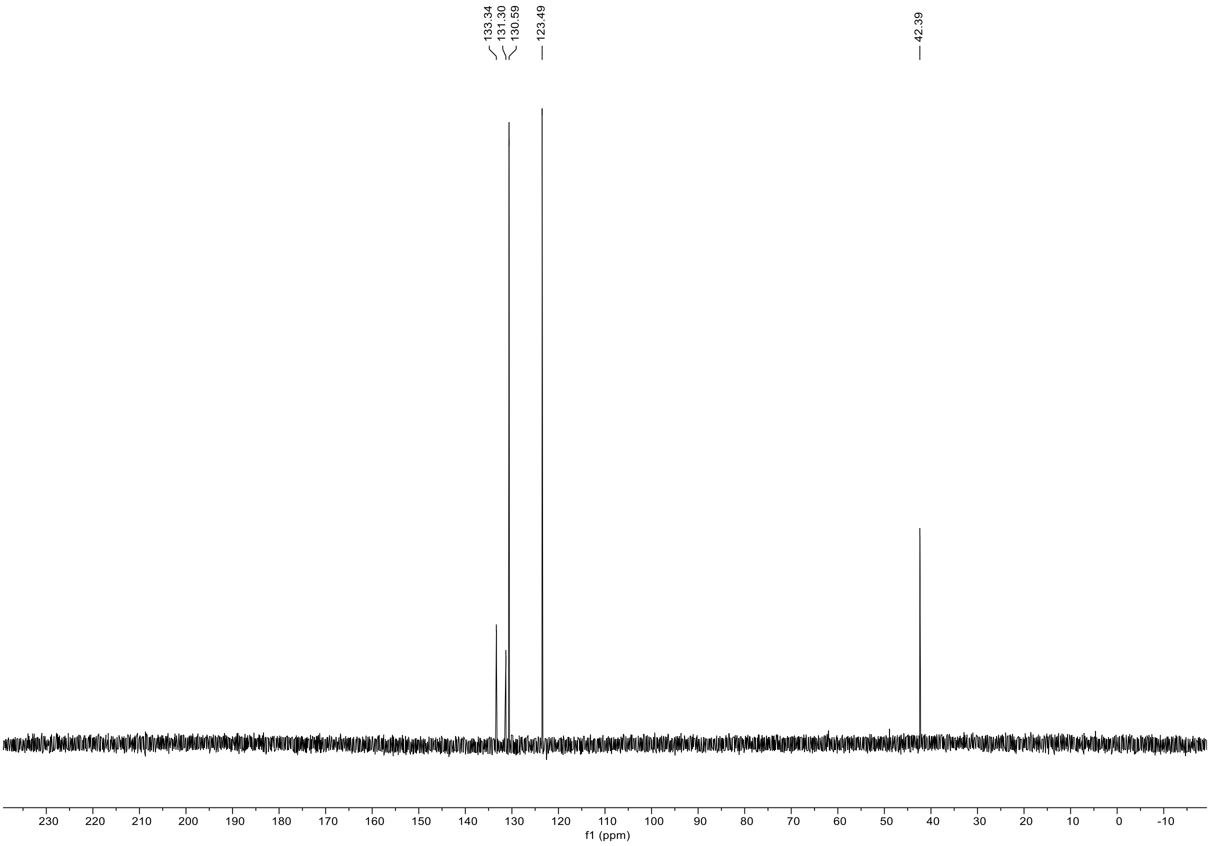

^{13}C NMR (151 MHz, D_2O) of 4-aminobenzylamine dihydrochloride (**1o**)

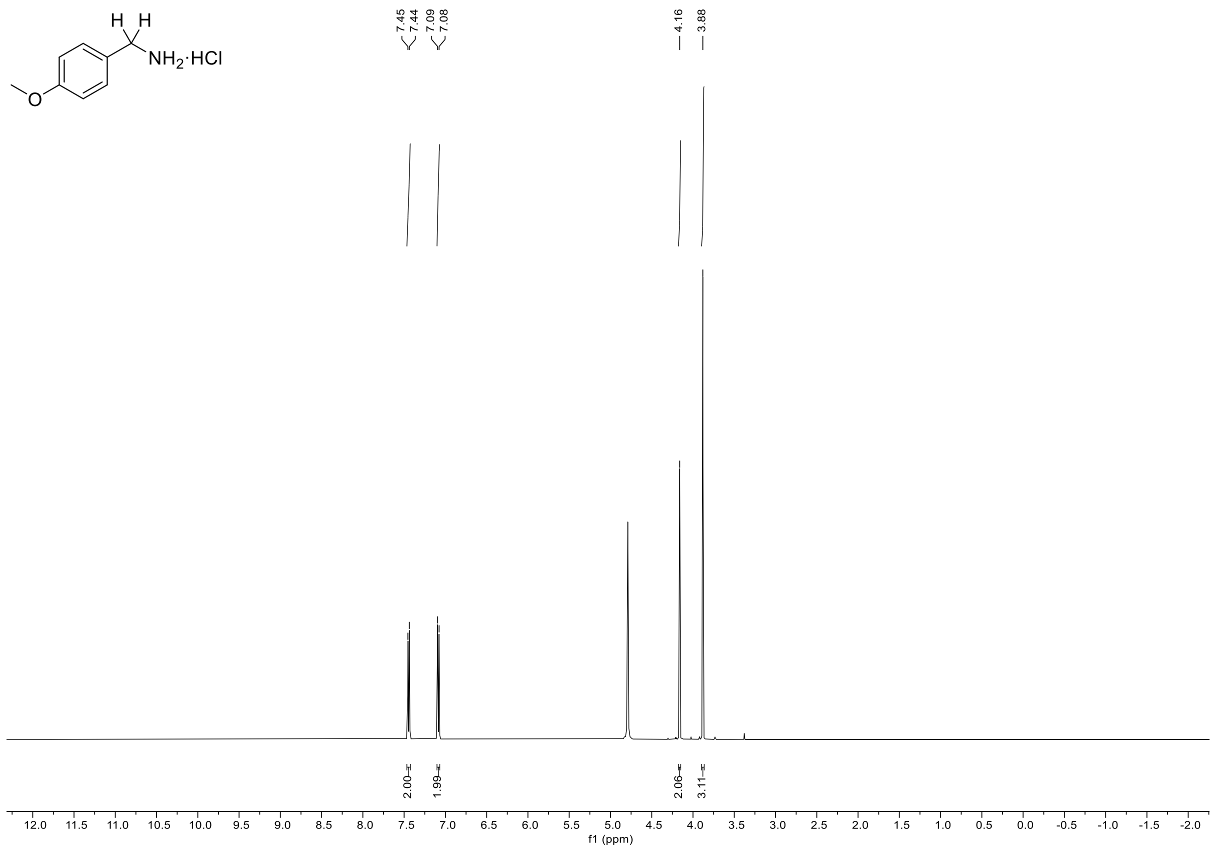

^{1}H NMR (500 MHz, D_2O) of 4-methoxybenzylamine hydrochloride (**1p**)

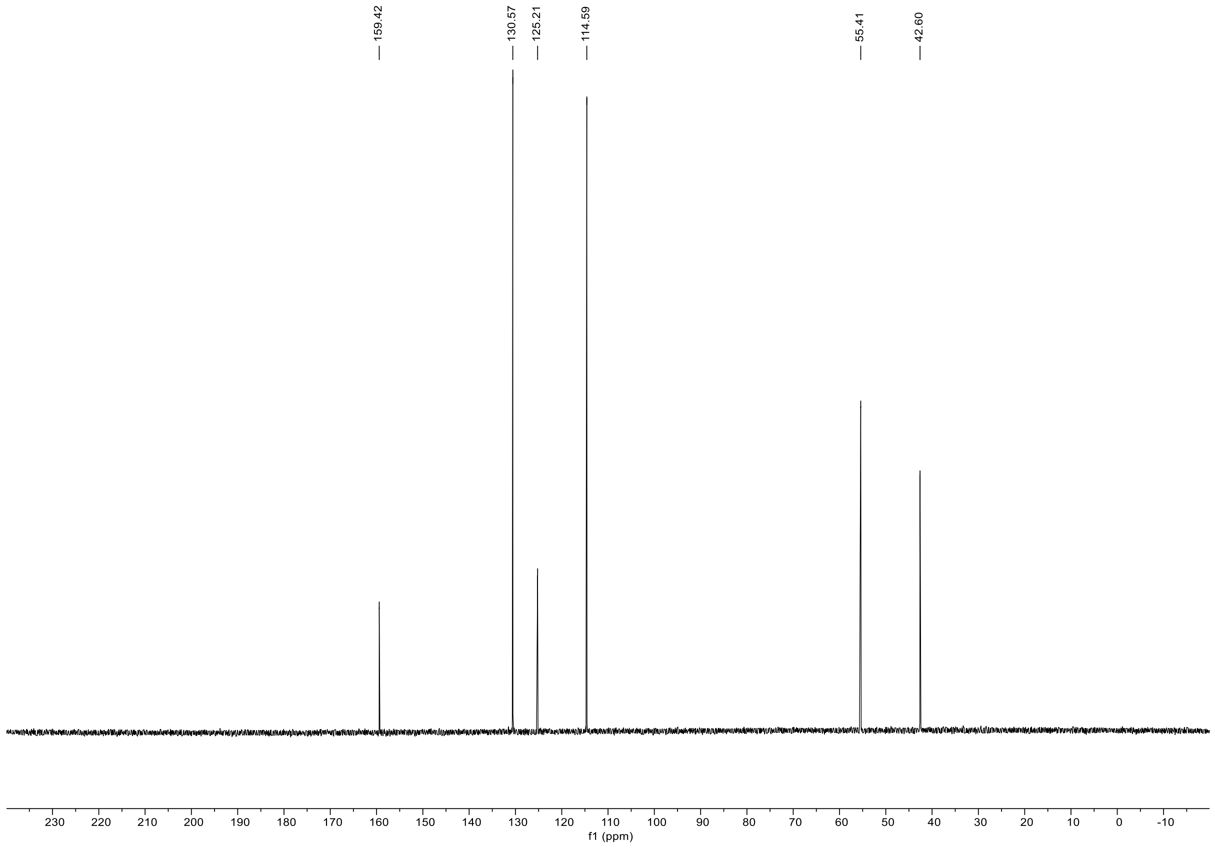

^{13}C NMR (126 MHz, D_2O) of 4-methoxybenzylamine hydrochloride (**1p**)

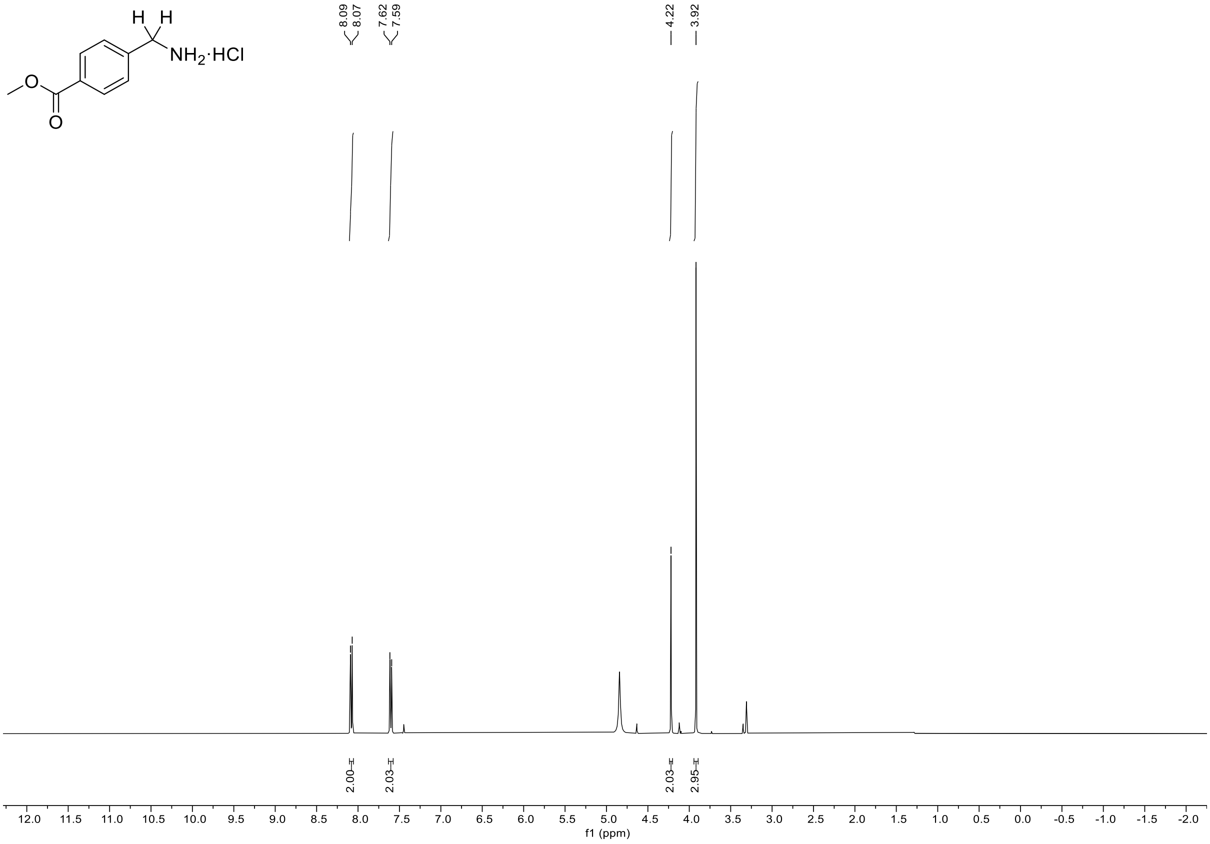

^{1}H NMR (400 MHz, methanol-*d*$_4$) of methyl 4-(aminomethyl)benzoate hydrochloride (**1q**)

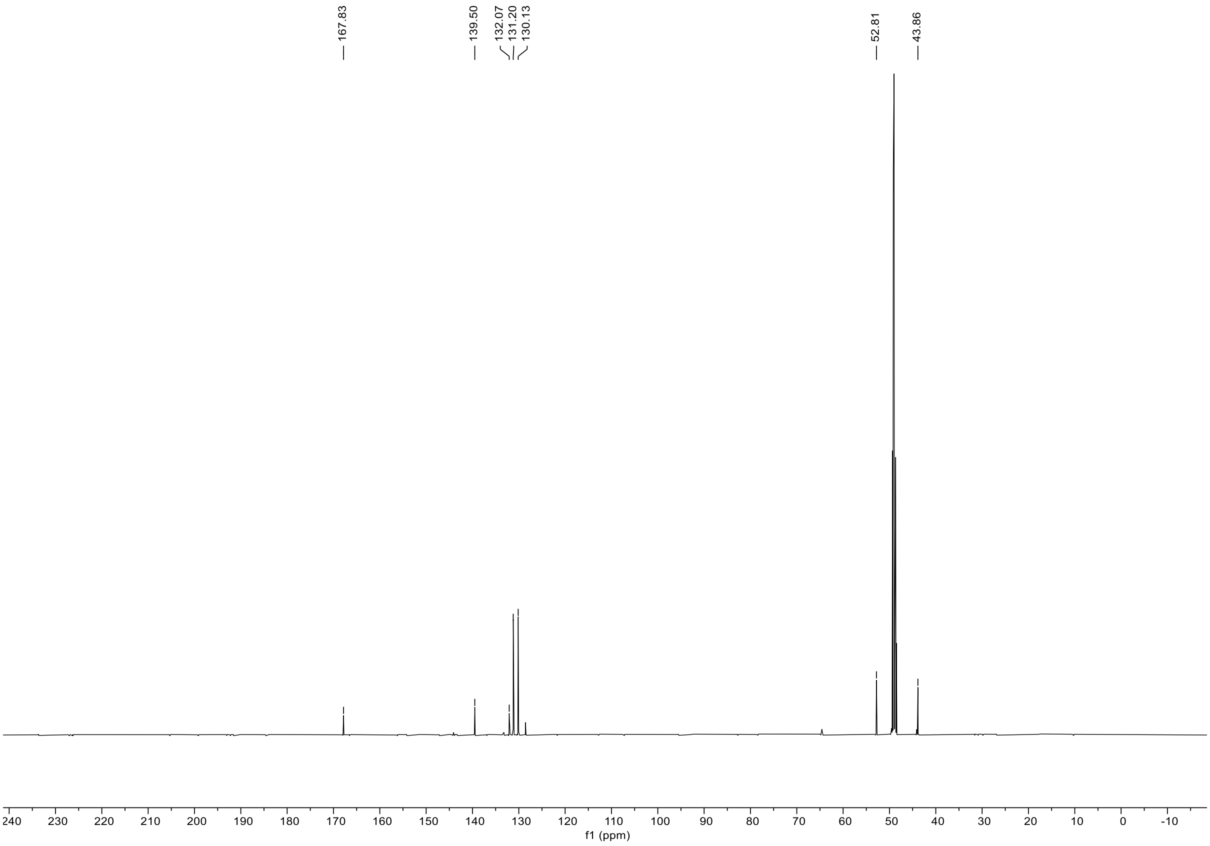

^{13}C NMR (126 MHz, methanol-*d*$_4$) of methyl 4-(aminomethyl)benzoate hydrochloride (**1q**)

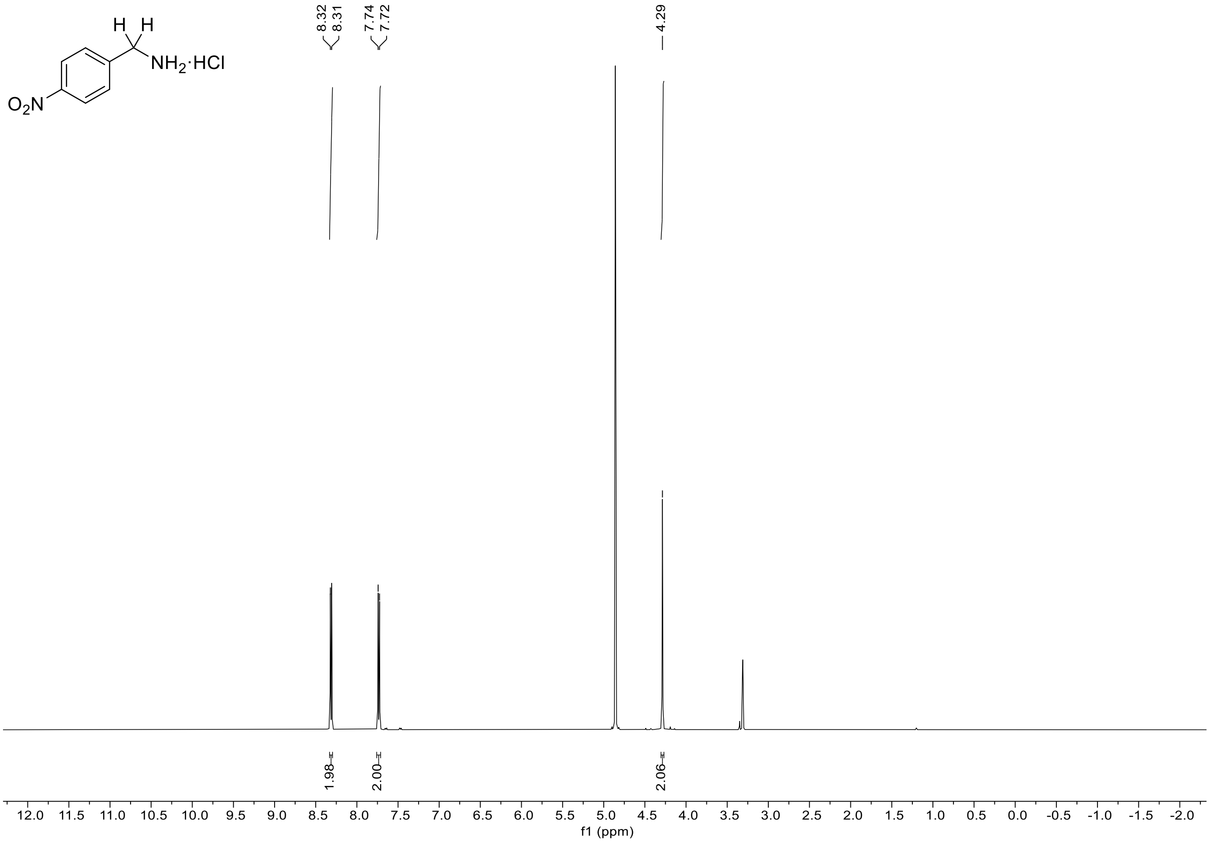

^{1}H NMR (500 MHz, methanol-d_4) of 4-nitrobenzylamine hydrochloride (**1r**)

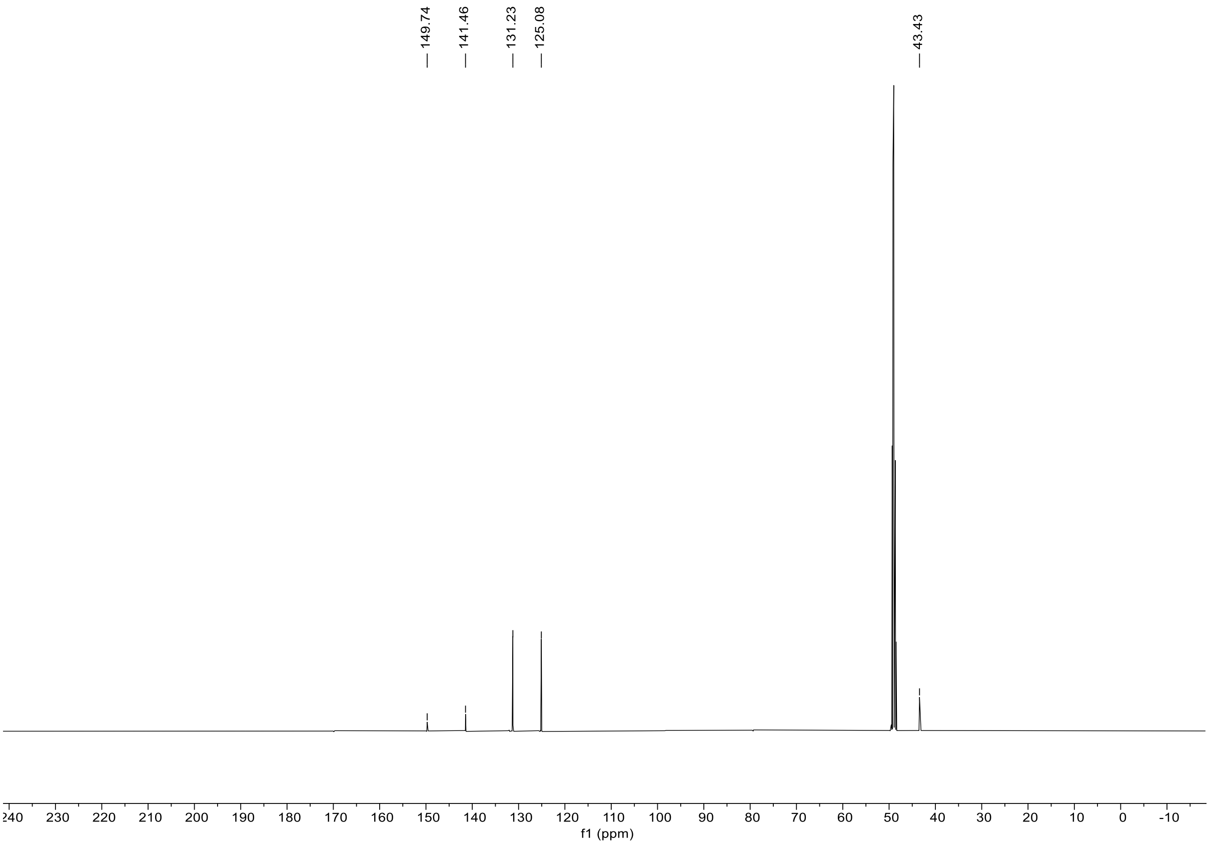

^{13}C NMR (126 MHz, methanol-d_4) of 4-nitrobenzylamine hydrochloride (**1r**)

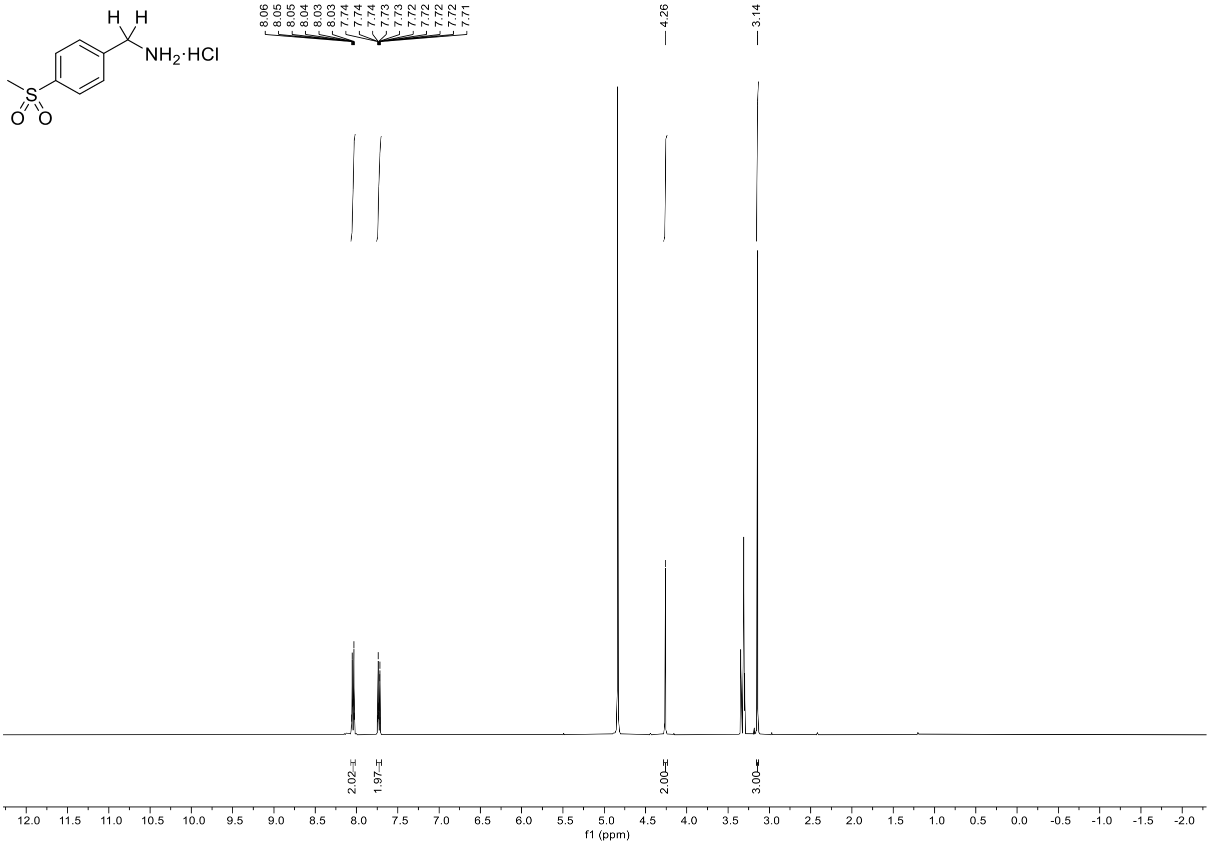

^{1}H NMR (400 MHz, methanol-*d*4) of 4-(methylsulfonyl)benzylamine hydrochloride (**1s**)

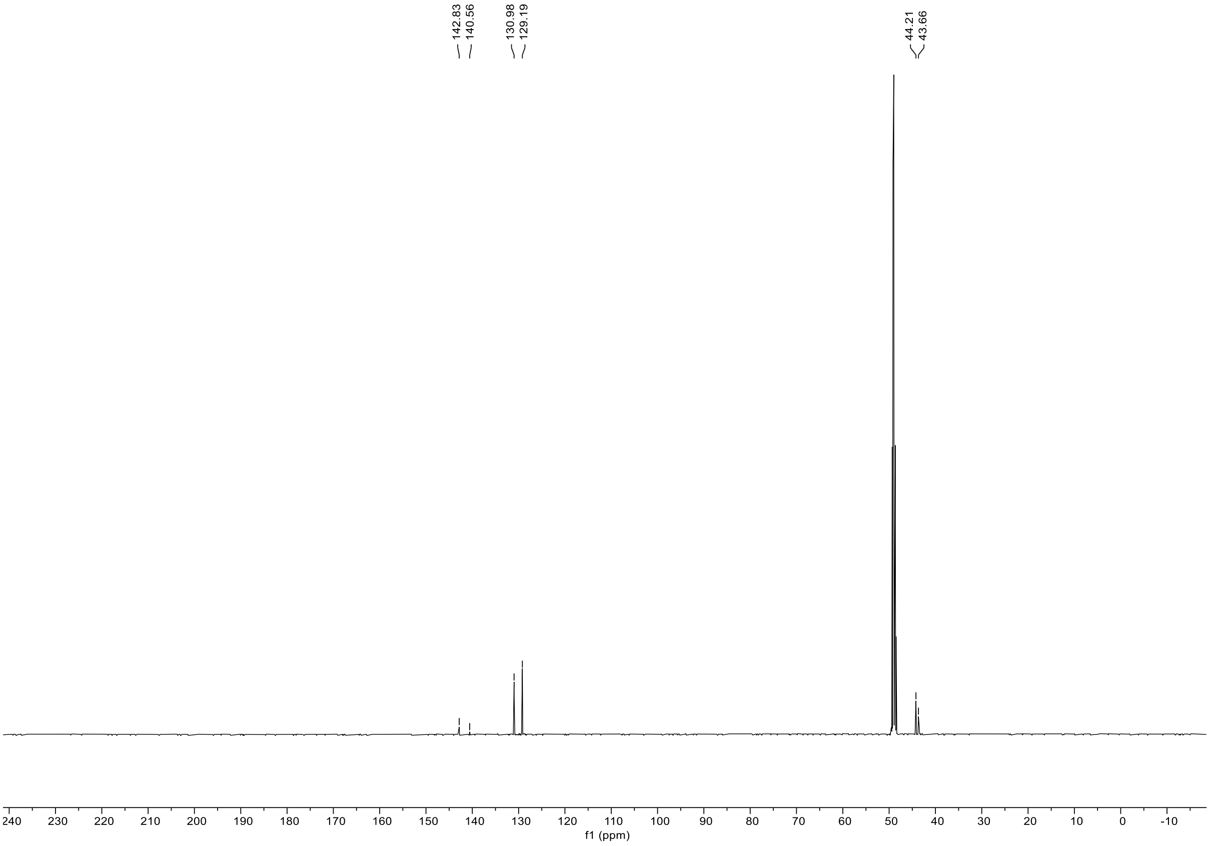

^{13}C NMR (126 MHz, methanol-*d*4) of 4-(methylsulfonyl)benzylamine hydrochloride (**1s**)

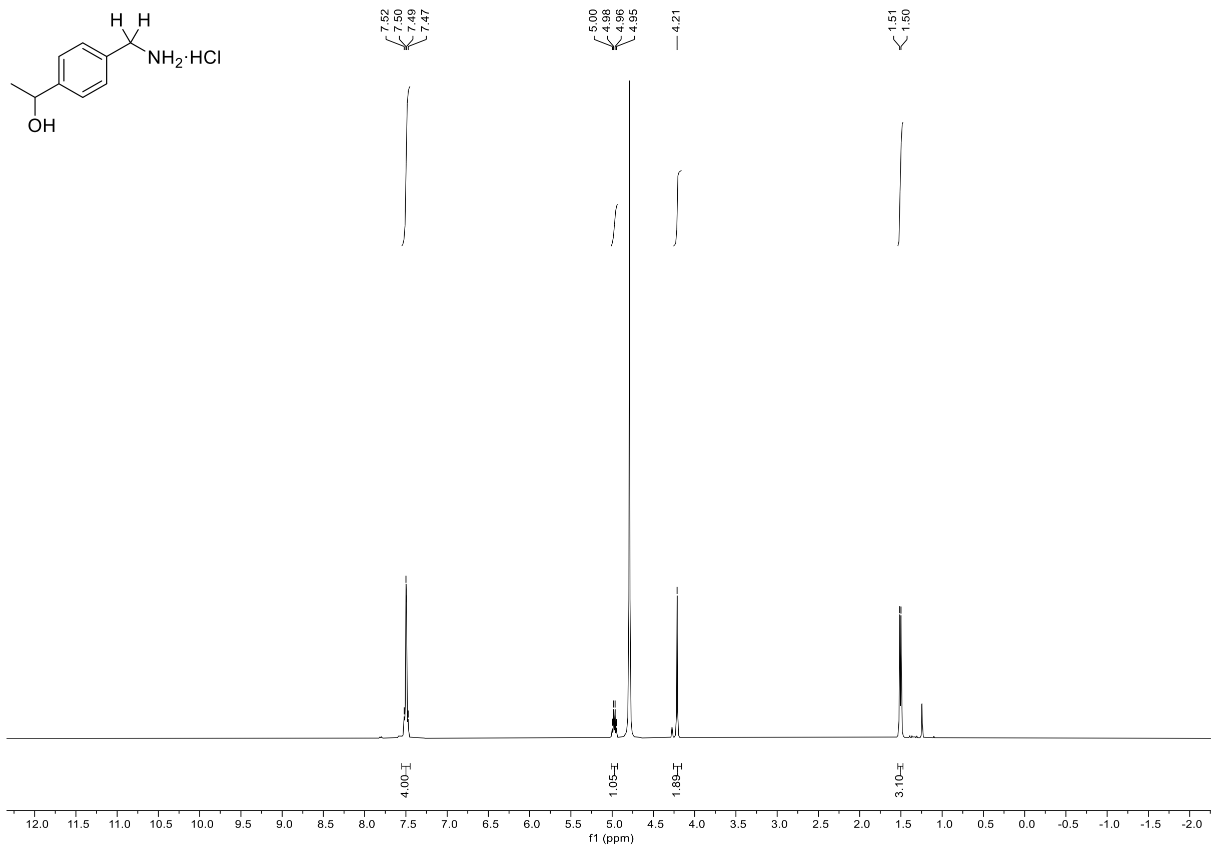

^{1}H NMR (400 MHz, methanol-d_4) of 4-(1-hydroxyethyl)benzylamine hydrochloride (**1t**)

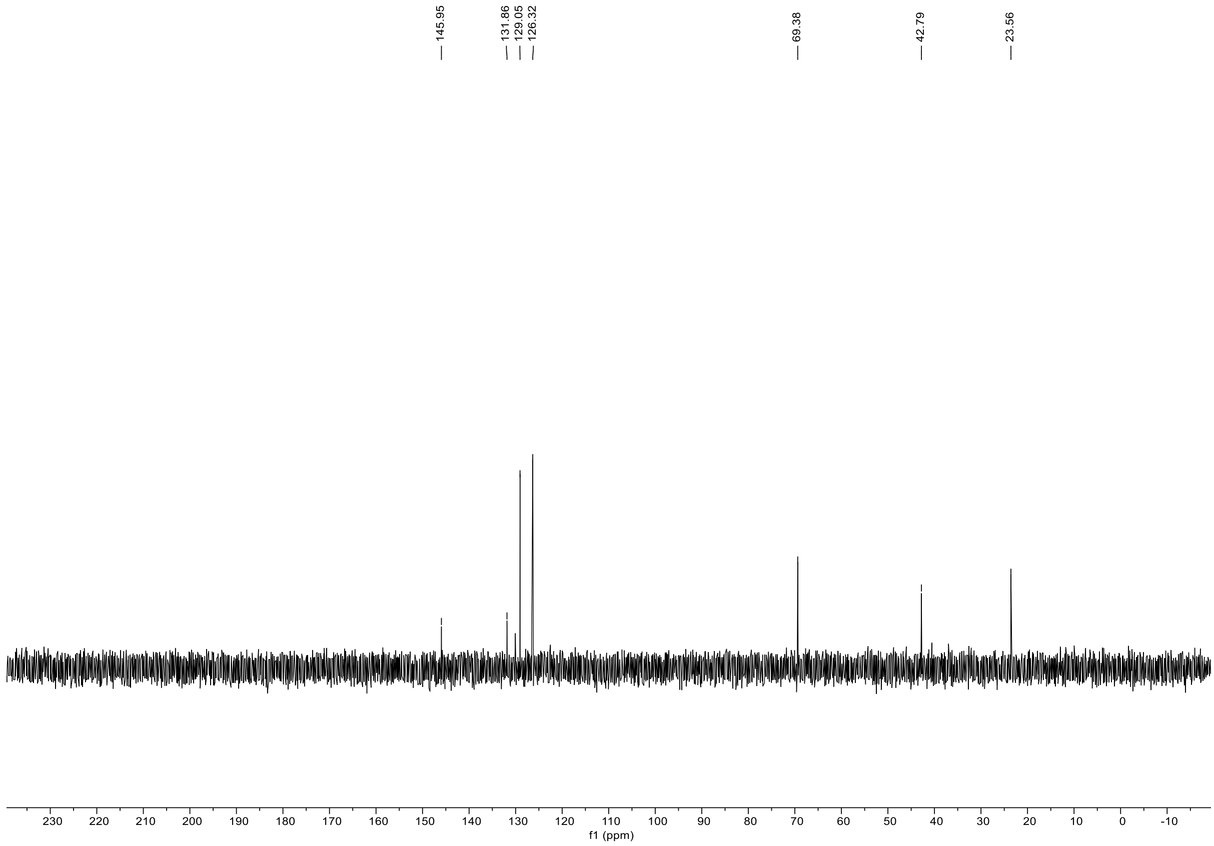

^{13}C NMR (101 MHz, methanol-d_4) of 4-(1-hydroxyethyl)benzylamine hydrochloride (**1t**)

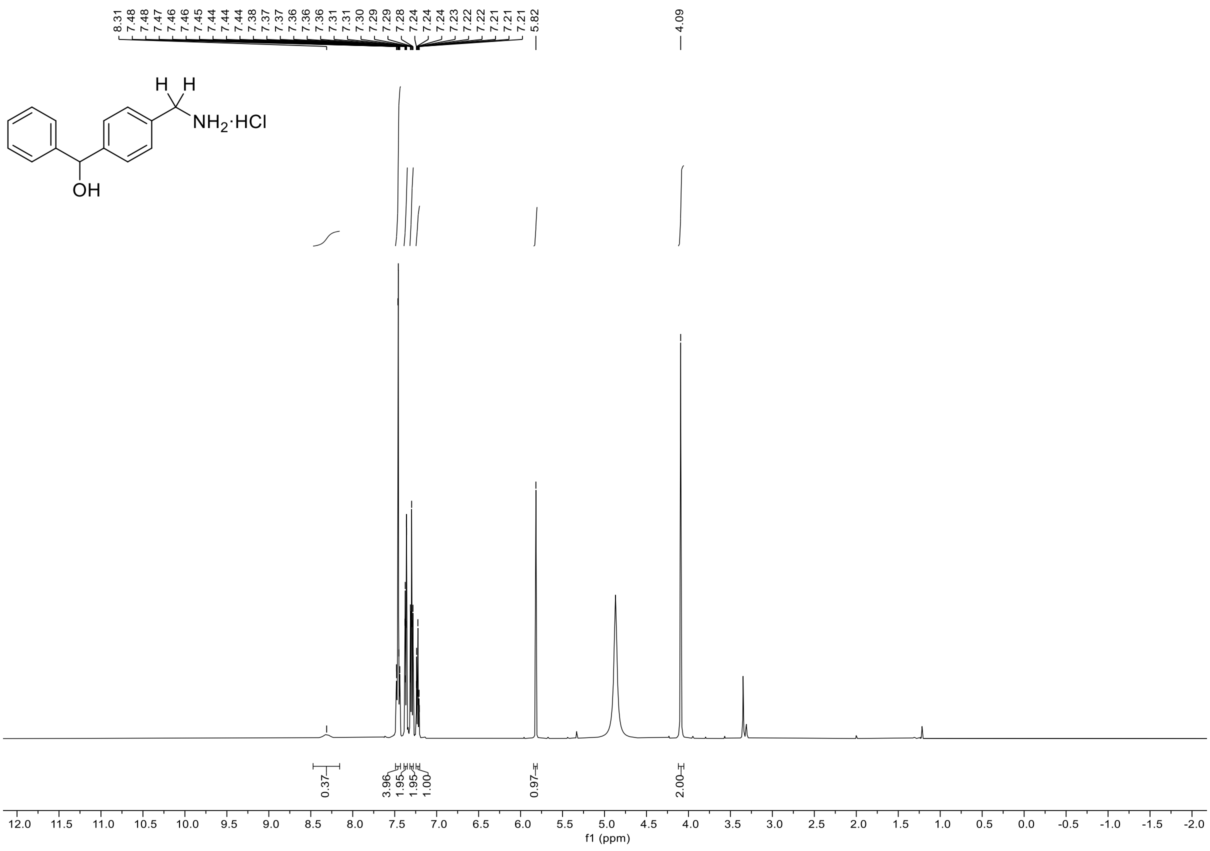

^{1}H NMR (500 MHz, methanol-d_4) of 4-(1-hydroxy-1-phenylmethyl)benzylamine hydrochloride (**1u**)

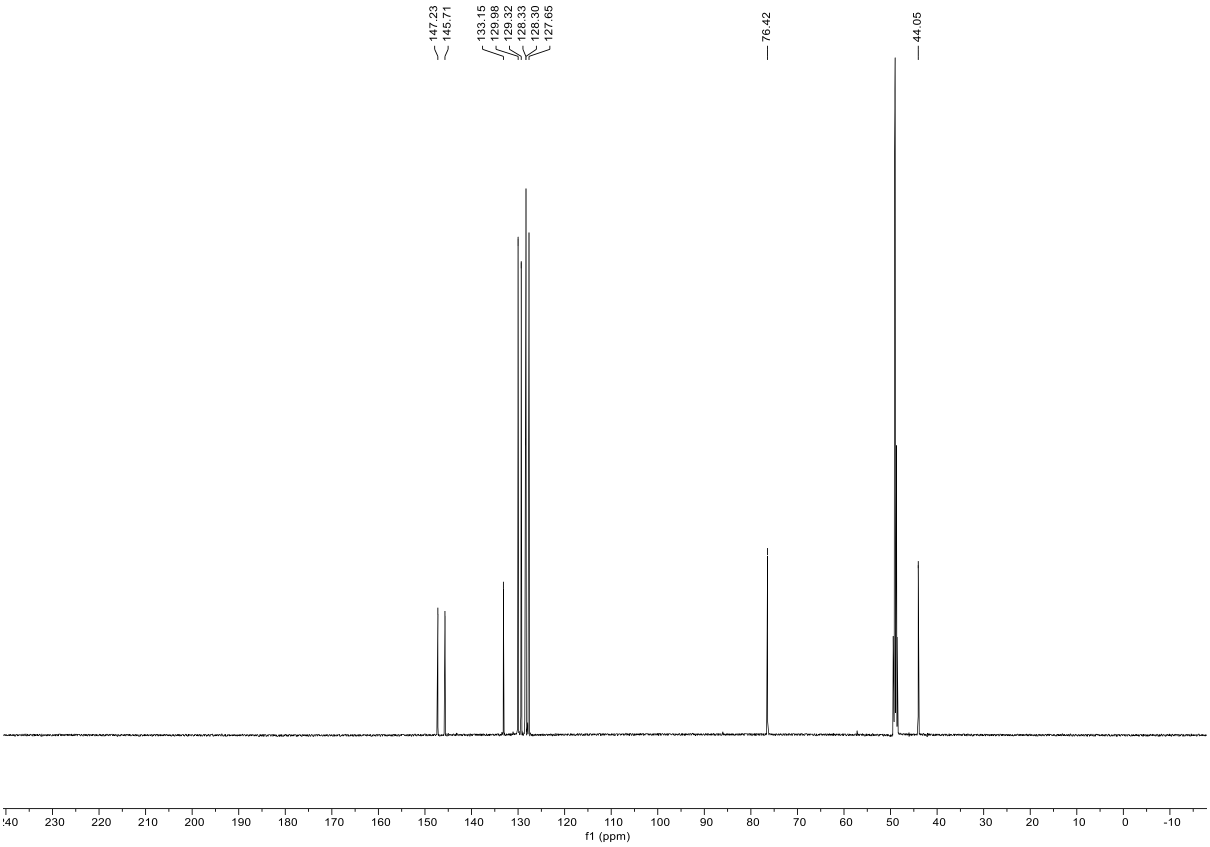

^{13}C NMR (151 MHz, methanol-d_4) of 4-(1-hydroxy-1-phenylmethyl)benzylamine hydrochloride (**1u**)

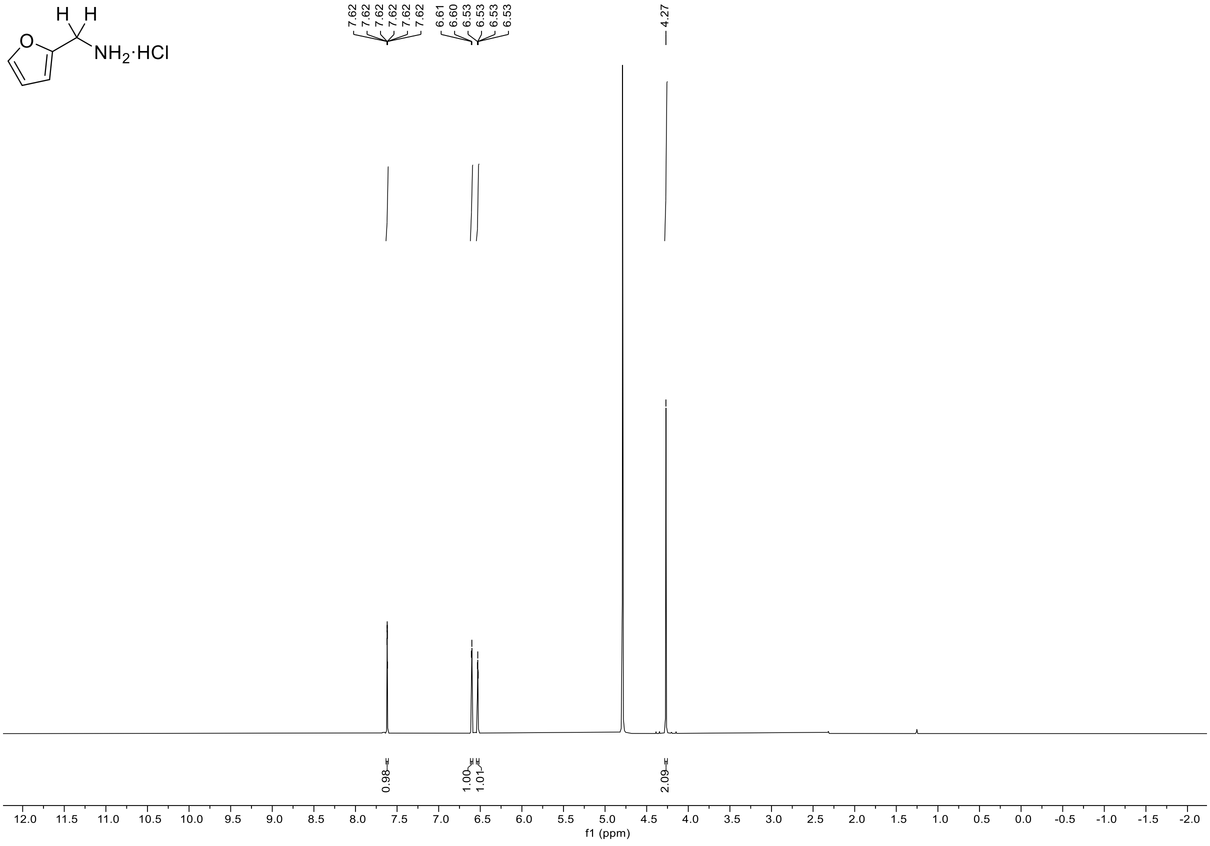

^{1}H NMR (600 MHz, D_2O) of furan-2-ylmethanamine hydrochloride (**1v**)

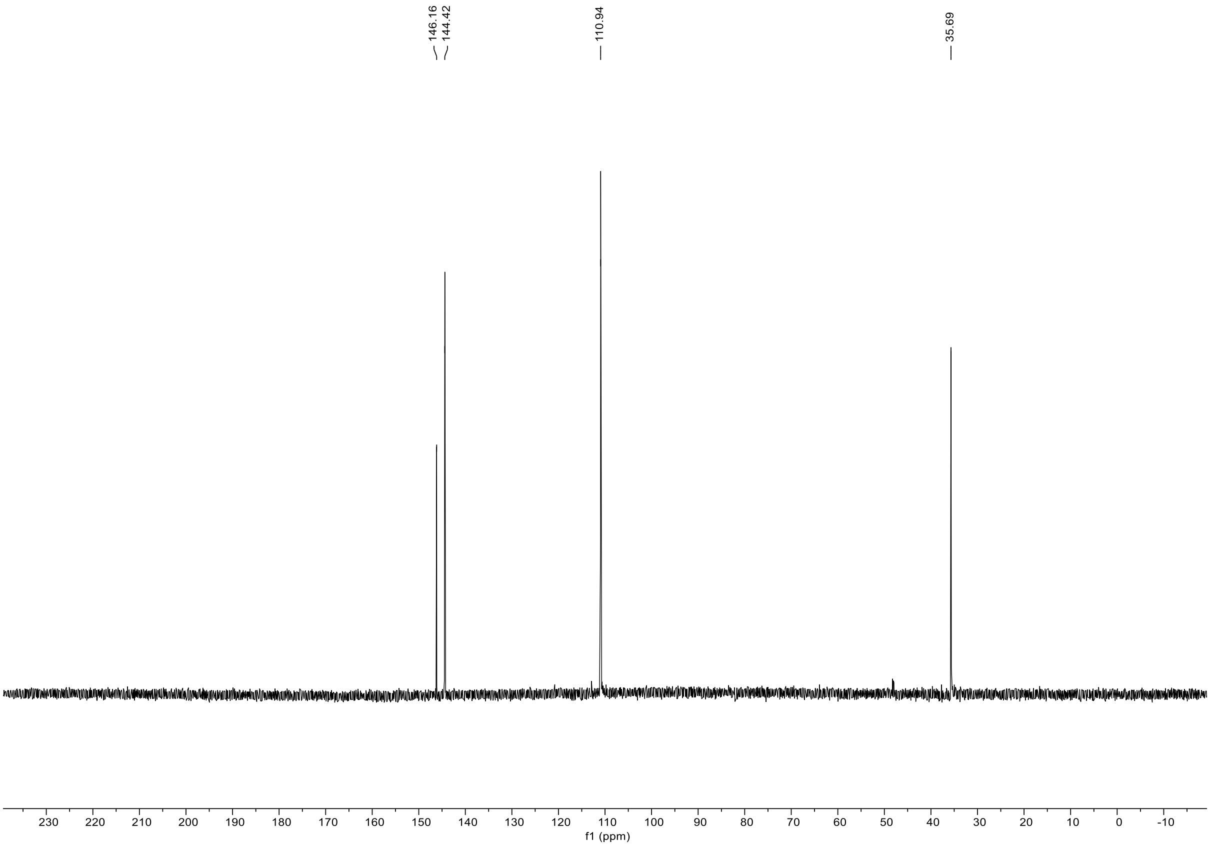

^{13}C NMR (151 MHz, D_2O) of furan-2-ylmethanamine hydrochloride (**1v**)

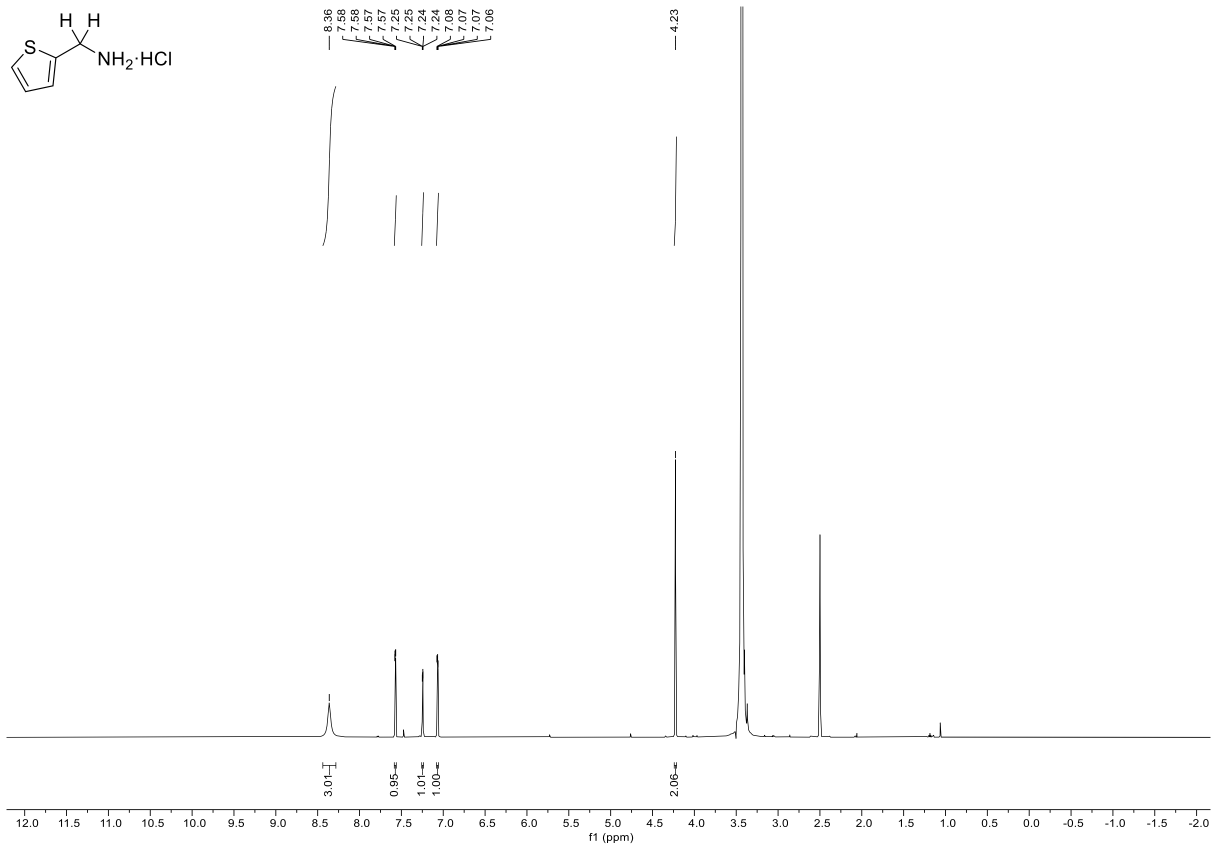

^{1}H NMR (600 MHz, DMSO-d_6) of thiophen-2-ylmethanamine hydrochloride (**1w**)

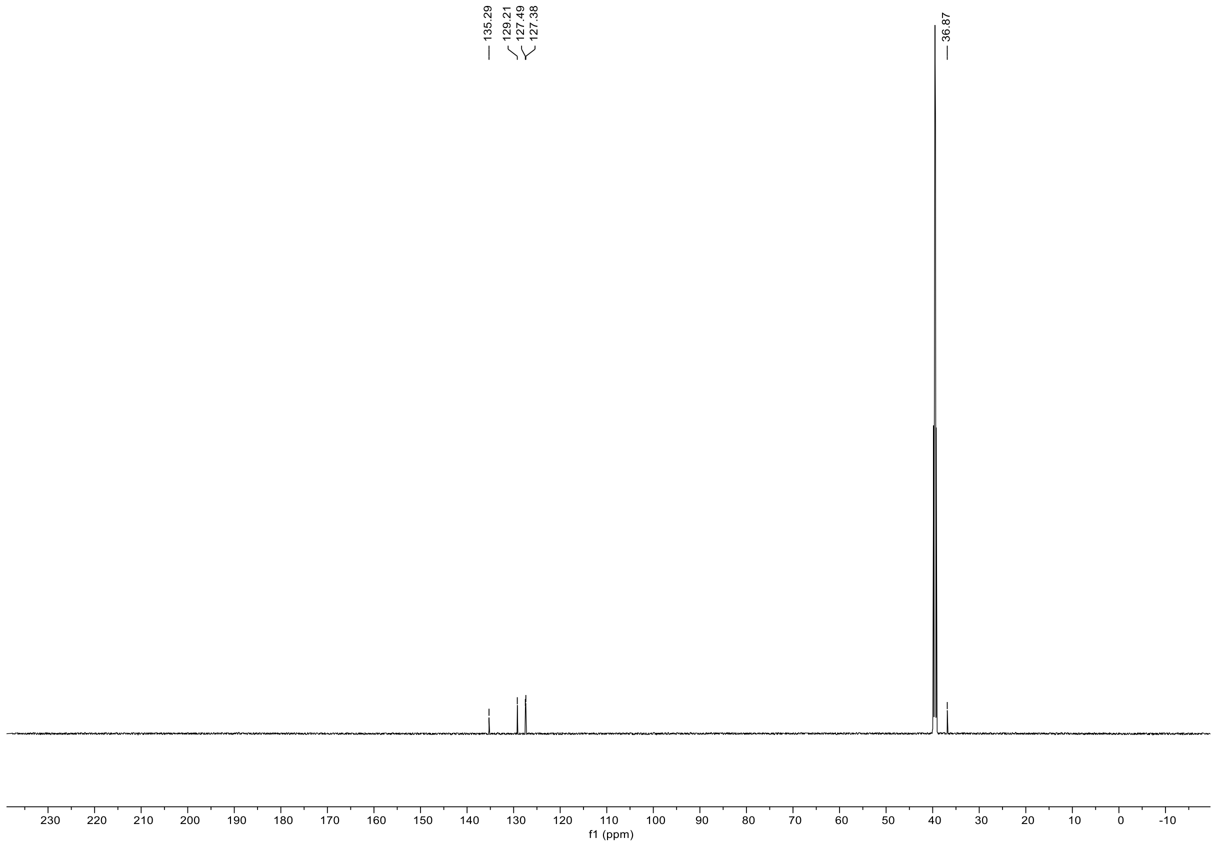

^{13}C NMR (151 MHz, DMSO-d_6) of thiophen-2-ylmethanamine hydrochloride (**1w**)

S9 References

1. S. Weber, L. F. Veiros and K. Kirchner, *Adv. Synth. Catal.*, 2019, **361**, 5412-5420.
2. S. Laval, W. Dayoub, L. Pehlivan, E. Métay, A. Favre-Reguillon, D. Delbrayelle, G. Mignani and M. Lemaire, *Tetrahedron*, 2014, **70**, 975-983.
3. M. Kitamura, T. Suga, S. Chiba and K. Narasaka, *Org. Lett.*, 2004, **6**, 4619-4621.
4. R. Adam, E. Alberico, W. Baumann, H.-J. Drexler, R. Jackstell, H. Junge and M. Beller, *Chemistry – A European Journal*, 2016, **22**, 4991-5002.
5. R. Labes, D. González-Calderón, C. Battilocchio, C. Mateos, G. R. Cumming, O. de Frutos, J. A. Rincón and S. V. Ley, *Synlett*, 2017, **28**, 2855-2858.
6. S. Elangovan, C. Topf, S. Fischer, H. Jiao, A. Spannenberg, W. Baumann, R. Ludwig, K. Junge and M. Beller, *Journal of the American Chemical Society*, 2016, **138**, 8809-8814.
7. L. Zhao, C. Hu, X. Cong, G. Deng, L. L. Liu, M. Luo and X. Zeng, *Journal of the American Chemical Society*, 2021, **143**, 1618-1629.
8. S. Wübbolt and M. Oestreich, *Synlett*, 2017, **28**, 2411-2414.
9. S. Weber, B. Stöger and K. Kirchner, *Org. Lett.*, 2018, **20**, 7212-7215.
10. D. C. Lenstra, J. J. Wolf and J. Mecinović, *The Journal of Organic Chemistry*, 2019, **84**, 6536-6545.
11. F. Yraola, S. García-Vicente, J. Fernández-Recio, F. Albericio, A. Zorzano, L. Marti and M. Royo, *J. Med. Chem.*, 2006, **49**, 6197-6208.
12. D. Timelthaler and C. Topf, *The Journal of Organic Chemistry*, 2019, **84**, 11604-11611.
13. K. Tokmic, B. J. Jackson, A. Salazar, T. J. Woods and A. R. Fout, *Journal of the American Chemical Society*, 2017, **139**, 13554-13561.
14. D. C. Lenstra, P. E. Lenting and J. Mecinović, *Green Chem.*, 2018, **20**, 4418-4422.
15. B. Paul, K. Chakrabarti and S. Kundu, *Dalton Transactions*, 2016, **45**, 11162-11171.
16. H. S. Das, S. Das, K. Dey, B. Singh, R. K. Haridasan, A. Das, J. Ahmed and S. K. Mandal, *Chem. Commun.*, 2019, **55**, 11868-11871.
17. M. J. Frisch, G. W. Trucks, H. B. Schlegel, G. E. Scuseria, M. A. Robb, J. R. Cheeseman, G. Scalmani, V. Barone, G. A. Petersson, H. Nakatsuji, X. Li, M. Caricato, A. V. Marenich, J. Bloino, B. G. Janesko, R. Gomperts, B. Mennucci, H. P. Hratchian, J. V. Ortiz, A. F. Izmaylov, J. L. Sonnenberg, D. Williams-Young, F. Ding, F. Lipparini, F. Egidi, J. Goings, B. Peng, A. Petrone, T. Henderson, D. Ranasinghe, V. G. Zakrzewski, J. Gao, N. Rega, G. Zheng, W. Liang, M. Hada, M. Ehara, K. Toyota, R. Fukuda, J. Hasegawa, M. Ishida, T. Nakajima, Y. Honda, O. Kitao, H. Nakai, T. Vreven, K. Throssell, J. A. Montgomery, Jr., J. E. Peralta, F. Ogliaro, M. J. Bearpark, J. J. Heyd, E. N. Brothers, K. N. Kudin, V. N. Staroverov, T. A. Keith, R. Kobayashi, J. Normand, K. Raghavachari, A. P. Rendell, J. C. Burant, S. S. Iyengar, J. Tomasi, M. Cossi, J. M. Millam, M. Klene, C. Adamo, R. Cammi, J. W. Ochterski, R. L. Martin, K. Morokuma, O. Farkas, J. B. Foresman, and D. J. Fox, Gaussian 16 (Revision C.01), Gaussian, Inc., Wallingford CT, 2016.
18. Y. Zhao, D. G. Truhlar, *Theor. Chem. Account*, 2008, **120,** 215–241.
19. Chemcraft - graphical software for visualization of quantum chemistry computations. https://www.chemcraftprog.com.
